# Stability of Carbocyclic Phosphinyl Radicals: Effect of Ring Size, Delocalization, and Sterics

**DOI:** 10.1021/acs.inorgchem.2c01968

**Published:** 2022-10-04

**Authors:** Anna Ott, Péter R. Nagy, Zoltán Benkő

**Affiliations:** † Department of Inorganic and Analytical Chemistry, Faculty of Chemical Technology and Biotechnology, Budapest University of Technology and Economics, Műegyetem rkp. 3, H-1111 Budapest, Hungary; ‡ Department of Physical Chemistry and Materials Science, Faculty of Chemical Technology and Biotechnology, Budapest University of Technology and Economics, Műegyetem rkp. 3, H-1111 Budapest, Hungary; § ELKH-BME Quantum Chemistry Research Group, Műegyetem rkp. 3, H-1111 Budapest, Hungary; ∥ ELKH-BME Computation Driven Chemistry Research Group, Műegyetem rkp. 3, H-1111 Budapest, Hungary

## Abstract

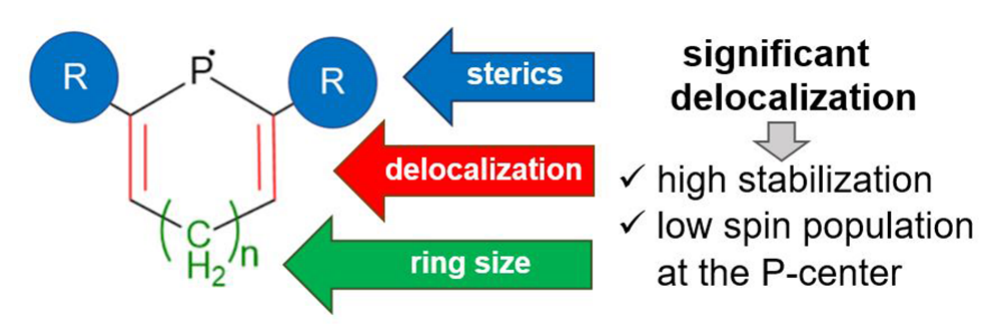

In this computational study, we report on the stability of cyclic phosphinyl radicals with an aim for a systematical assessment of stabilization effects. The radical stabilization energies (RSEs) were calculated using isodesmic reactions for a large number of carbocyclic radicals possessing different ring sizes and grades of unsaturation. In general, the RSE values range from −1.2 to −14.0 kcal·mol^–1^, and they show practically no correlation with the spin populations at the P-centers. The RSE values correlate with the reaction Gibbs free energies calculated for the dimerization of the studied simple radicals. Therefore, the more easily accessible RSE values offer a cost-effective estimation of global stability in a straightforward manner. To explore the effect of unsaturation on the RSE values, delocalization energies were determined using appropriate isodesmic reactions. Introducing unsaturations beside the P-center into the backbone of the rings leads to an additive increase in the magnitude of the delocalization energy (∼10, 20, and 30 kcal·mol^–1^, respectively, for radicals with one, two, and three C=C bonds in the conjugation). Parallelly, the spin populations at the P-centers also dwindle gradually by ∼0.1 e in the same order, indicating that the lone electron delocalizes over the π-system. Radicals containing exocyclic C=C π-bonds were also investigated, and all of these radicals have rather similar stabilities independently of the ring size, outlining the primary importance of the two exocyclic π-bonds in the conjugation. Among the radicals involved in our study, those with the best electronic stabilization are the unsaturated three-, five-, six-, and seven-membered rings containing the maximum number of conjugated vinyl fragments. The largest delocalization energy of 31.5 kcal·mol^–1^ and the lowest obtained spin population of 0.665 e were found for the fully unsaturated seven-membered radical (phosphepin derivative). Importantly, the electronic stabilization effects alone are insufficient for stabilizing the radicals in monomeric forms epitomized by the exothermic dimerization energies (−40 to −58 kcal·mol^–1^). Therefore, it is essential to apply sterically demanding bulky substituents on the α-C-atoms. Tweaking the steric congestion enabled us to propose radicals that are expected to be stable against dimerization and, consequently, may be realistic target species for synthetic investigations. The effects contributing to the stability of radicals having sterically encumbered substituents have also been explored.

## Abstract 2

To systematically evaluate the stabilization effects, the radical stabilization energies of various carbocyclic phosphinyl radicals having saturated backbones or unsaturation(s) in either endocyclic or exocyclic manner have been determined and analyzed. As the electronic stabilization is alone insufficient to hamper the possible dimerization of these species, the effect of several sterically demanding substituents has been explored for the congeners with best electronic stabilizations, thus enabling us to propose synthetically accessible candidates in the future.

## 1 Introduction

Radicals play an unquestionably important role in organic/inorganic chemistry and even in biochemistry,^1,2^ and consequently, they gain increasing interest from both synthetic and computational aspects. Following the discovery of trityl radical (Ph_3_C^•^) by Gomberg more than 120 years ago,^3^ the chemistry of radicals with main group elements has become a continuously expanding field. Although several N-containing radicals such as NO, NO_2_, and aminoxyl derivatives are textbook examples, stable or at least persistent radicals containing exclusively main group elements can still be considered curiosities because the dimerization of the radicals via the formation of a rather strong covalent bond is usually favored energetically. Being aware of the well-known diagonal rule in the periodic table of the elements exemplified by the similarity between carbon and phosphorus,^4−6^ it is not surprising that the interest continues to grow toward stable and persistent radicals of the carbon-like phosphorus. Importantly, phosphorus-containing radicals allow for many different applications. For example, P-centered radicals can be employed in polymerization reactions as initiators originating from the light-induced homolytic P–C bond dissociation of mono- or bisacyl-phosphane oxides.^7^ Furthermore, P-radicals may play a role in the extinction process of burning underlining the importance of phosphorus-based flame retardants.^8^ Due to the appealing properties of the ^31^P nucleus (100% natural abundance and 1/2 spin), electron paramagnetic resonance (EPR) spectroscopy of P-containing paramagnetic species offers promising possibilities in structural investigations,^9^ as well as in spin labeling,^10−12^ which may facilitate applications for understanding chemical and biological processes. Additionally, P-containing radicals can be important reactants in various chemical syntheses, e.g., in single-electron transfer reactions.^13−15^

If miscellaneous examples such as P-radicals coordinated to metal centers^16^ are not considered, neutral P-centered radicals can be divided into three larger groups: phosphonyl (R_2_P^•^=O), phosphinyl (R_2_P^•^), and phosphoranyl (R_4_P^•^) radicals.^13,17^ In the case of phosphonyl radicals, the O-atom significantly affects the stability due to resonance stabilization (R_2_P^•^=O ↔ R_2_P–O^•^), and the effect of the other substituents is typically surpassed by the O-center.^13^ As expected from simple considerations, these radicals have pyramidal geometries. Phosphoranyl radicals may offer greater structural diversity, as in principle all of the four ligands can be varied; however, this can be difficult to achieve synthetically. Probably, the most challenging class is that of phosphinyl radicals, in which the P-center possesses a lone pair as well as an unpaired electron, and the latter typically occupies the 3p-type orbital—leading to a so-called π**-**radical (as commonly known also for N-centered analogues).^18^ Besides neutral radicals, a plethora of P-based radical ions^19−25^ (both anions and cations), as well as stable biradicals have already been reported.^26−29^

Although the homolytic bond dissociation energy (BDE) of a P–P bond (61.2 kcal·mol^–1^ in H_2_P–PH_2_^30^) is significantly lower than that between the “diagonal congener” carbon atoms (90.2 kcal·mol^–1^ for the C–C bond in H_3_C–CH_3_^30^), it is still substantial, offering thermodynamic driving force for the dimerization of the radicals.^31^ As a result, dimerization endangers the isolation of P-radicals even in the absence of further reactants in the solution and the possibly most effective stabilization of radicals is clearly of high importance. In general, two main effects govern the stability of radicals: steric and electronic effects. The steric stabilization can be achieved by careful selection of bulky substituents, and several species have been accessed employing this methodology.^32−43^ The origin of this effect is that the flanking large ligands cause steric congestion via Pauli repulsion; thus, the crowded radical centers are less prone to dimerization. However, a large number of weak dispersion interactions may arise between the sterically demanding ligands, and this can counterproductively result in substantial stabilization of the dimeric form.^44,45^ In contrast, the electronic stabilization can be enhanced by introducing functional groups next to the P-center enabling resonance stabilization via delocalization, that is electronic communication of the lone electron with the neighboring centers.

As our purpose is to scrutinize the stability of phosphinyl radicals, in the following, a selection of the most important examples is presented; however, we note that further derivatives are also known.^33−35,46−52^

**Figure 1:**
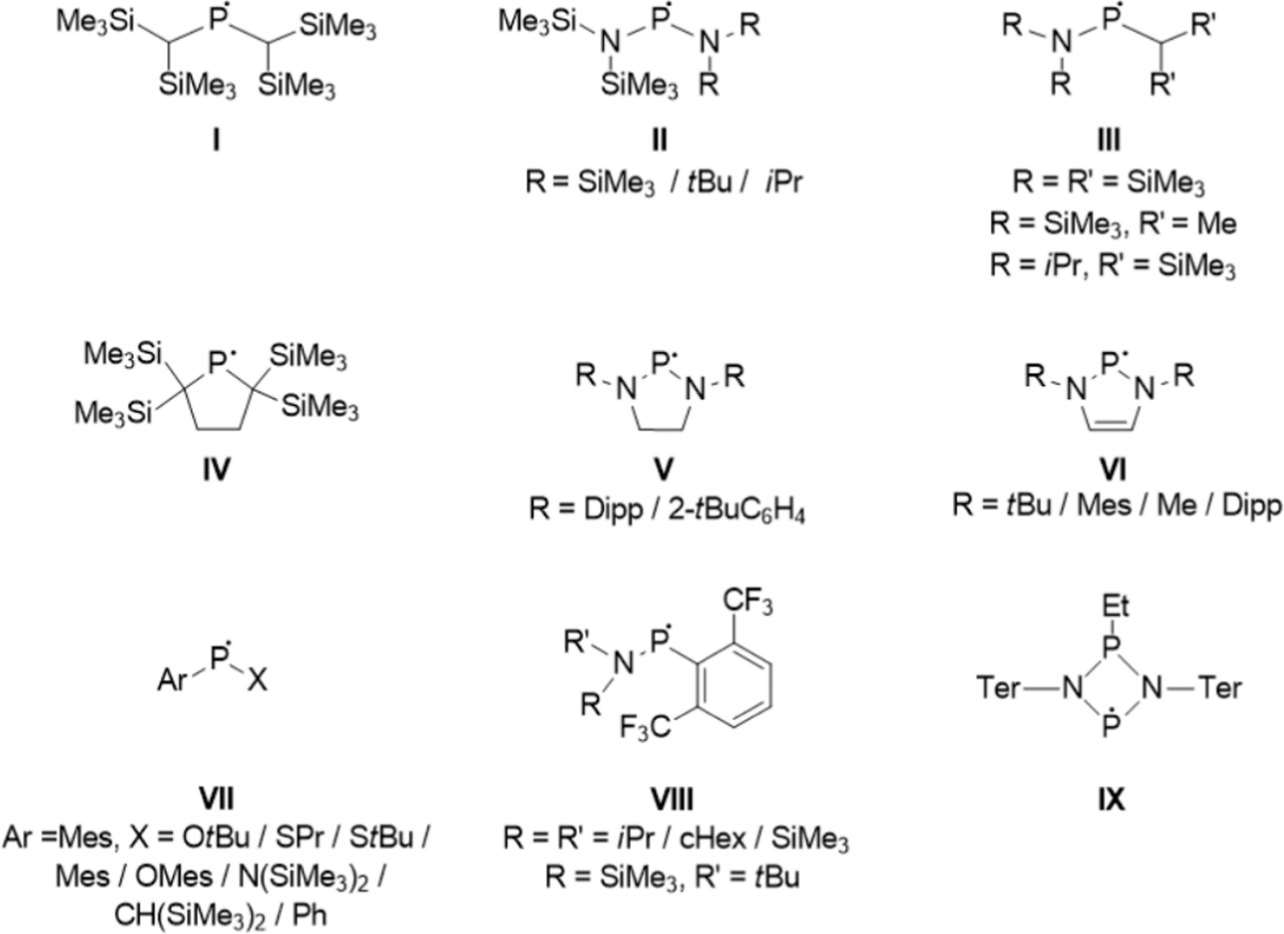
Selected examples of neutral phosphinyl radicals (Dipp = 2,6-di-iso-propylphenyl, Mes = 2,4,6-trimethylphenyl, cHex = cyclohexyl, Ter = 2,6-bis-(2,4,6-trimethylphenyl)-phenyl).

The first persistent dialkyl-phosphinyl radical (**I** in Figure 1) was reported by Lappert et al.^36^ and later on several further acyclic derivatives, for example, the symmetric diamino species **II**, and the unsymmetric, alkyl-amino species **III** were also accessed synthetically.^17,37,53^ In these radicals, the steric congestion of the ligands plays an important role in the stabilization.

The highly air-sensitive but thermally stable cyclic radical **IV** was discovered by Ishida and co-workers in 2011.^38^ Although this radical is similar to the acyclic species **I**, in radical **IV**, the two α-carbon centers are embedded into a five-membered ring and the geometrical constrain hampers the dimerization in the solid state. The cyclic versions of diamino-substituted phosphinyl radicals (**V**) were also accessed;^17,54^ however, these radicals only exhibit moderate stabilities and partially dimerize to diphosphines in equilibrium reactions.

Besides the N-heterocyclic analogues with saturated backbones, their unsaturated congeners (**VI**) have also been reported. These species are typical π-radicals, but the stabilization arises dominantly from steric factors and the dimerization equilibria are typically shifted to the side of the dimeric forms.^55,56,39^

Furthermore, a series of unsymmetrically substituted radicals that contain a stabilizing heteroatom (O, S, etc.) and an aryl substituent (**VII**) have been described. Fluorinated aryl rings accompanied by amino substituents were also investigated for the stabilization of P-centered radicals,^40^ and these species (**VIII**) can also be considered as π-radicals. Very recently, a four-membered P-radical with bulky aryl-amino substituents (**IX**) has been reported as a stable species.^43^

**Figure 2:**
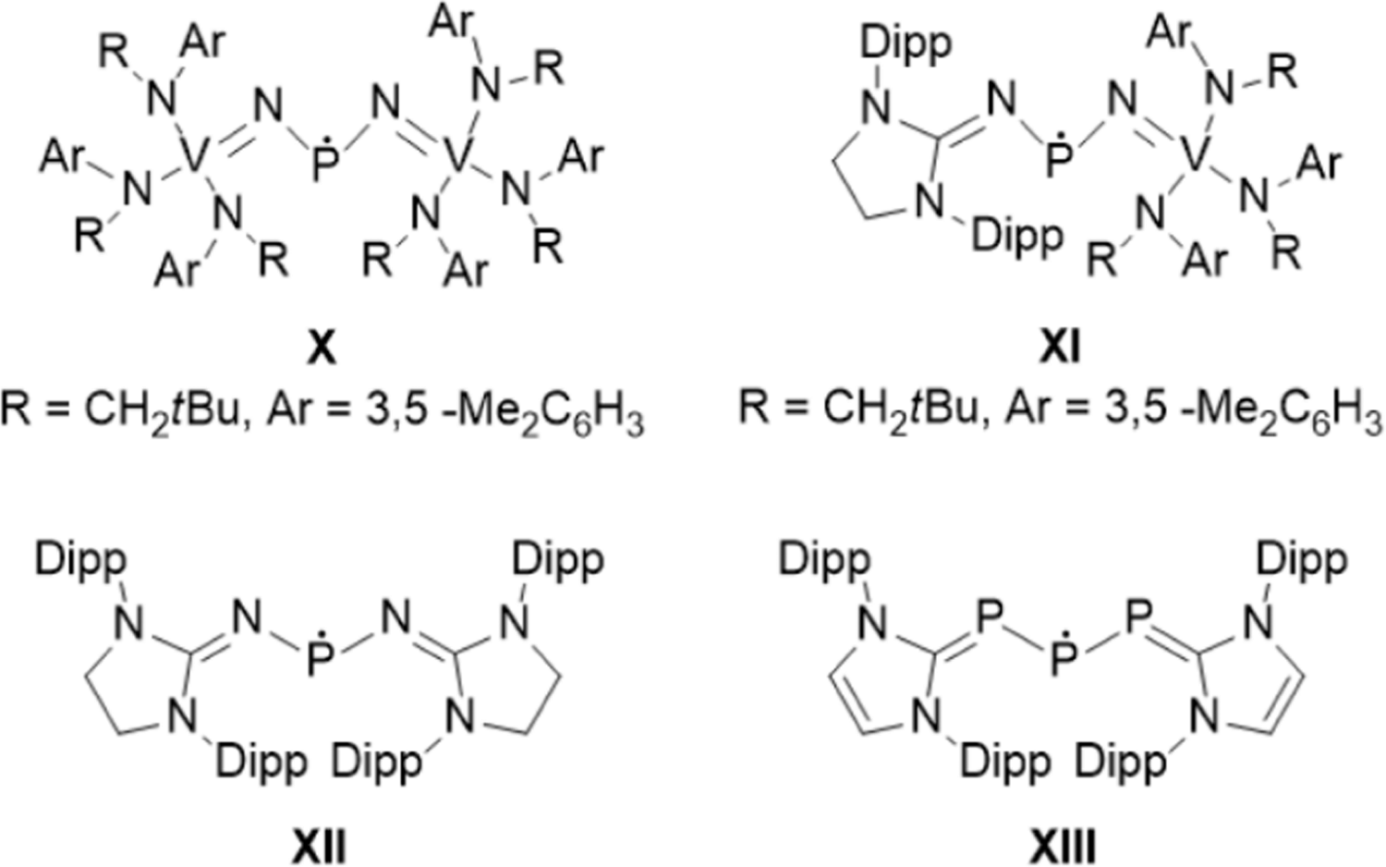
Selected examples of phosphinyl radicals with dominant π-delocalization.

Complementing the steric effects, the radicals can be further stabilized electronically; in this case, the unpaired electron at the P-center is delocalized over a conjugated π-system. Selected examples stabilized by additional π-delocalization are shown in Figure 2. Importantly, the radical bearing nitrovanadium(V)-trisanilide ligands (**X**) is stable as a monomer even in the solid state,^42^ and the stabilization is predominantly attributed to the vanadium(V)/vanadium(IV) redox couple (−P^•^–N=VR_3_′ ↔ −P=N–^•^VR_3_′). Later, in a modified version of the former species, one of the nitrovanadium(V)-trisanilide ligands was formally replaced by a cyclic guanidine moiety (**XI**).^57^ In this “mixed” compound, the atomic spin density is again located dominantly on the vanadium center, underlining the deterministic importance of the transition-metal fragment in the resonance stabilization. By introducing symmetrically the guanidine substituents leading to radical **XII**, the spin density can effectively be shifted to the phosphorus center. Isodesmic reactions have also shown that the two guanidine groups offer significantly lower stabilization than the vanadium-containing ligands.^57^ Instead of nitrogen, phosphorus can also be used for stabilization, such as in radical **XIII**,^58^ According to experimental EPR data and density functional theory (DFT) calculations, the spin population is mainly on the central P-atom.

As it can clearly be seen from the above examples, the majority of phosphinyl radicals accessed successfully so far has acyclic structure and contains at least one stabilizing heteroatom, typically in the proximity of the radical center (either N, O, S, P, etc. in the α-positions, or silyl substituents in the β-positions as in examples **I** and **IV**). This raises the question whether it is possible to obtain stable P-radicals exclusively with carbon framework, that is without introducing further special heteroelements. As the various cyclic structures may provide an additional stabilization effect by decreasing the flexibility, and in general, straightforward cyclization procedures are known in the literature for the synthesis of P-heterocycles, we envisaged a systematic investigation of saturated and unsaturated carbocyclic P-radicals. Our further aim is to get a deeper insight into the stabilization effects, especially regarding delocalization, which can help the effective design and eventually synthetic realization of new radicals.

## 2 Results and Discussion

The article is organized in the following way: First, the most important concepts used in this research are summarized. Then, it will be shown that vinyl groups offer substantial electronic stabilization, exceeding even that of the imino groups applied commonly in the experiments. In the next section, we scrutinize systematically the electronic stabilization effects in simple heterocyclic species: to present the results in a clear way, these radicals were separated into groups on the basis of their structures (saturated rings, rings with one or more endocyclic or exocyclic C=C bonds). After selecting the radicals that are the most suitable for synthetic purposes, the effect of several bulky substituents on the stability against dimer formations will be addressed.

### 2.1 Methodology

Although the clear separation of steric and electronic effects is difficult, we followed the rationale that for simple systems having only H substituents, the steric effects are negligible and thus the electronic effects can be studied separately. To quantify these effects, we applied radical stabilization energies (RSEs) and dimerization energies.

**Scheme 1:**
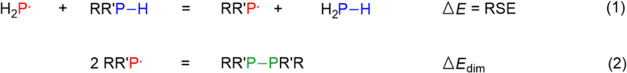
Isodesmic Reaction Providing RSE and Dimerization Reaction for Phosphinyl Radicals

A straightforward way to assess the relative stability of radicals employs the well-established concept of isodesmic H-transfer reactions,^18^ which delivers radical stabilization energies (RSEs). Generally, for phosphinyl radicals, the hypothetical isodesmic reaction eq 1 (Scheme 1) can be used. These computations offer information predominantly on the thermodynamical stabilization induced by electronic effects for a given radical (RR′P^•^), compared to the parent H_2_P^•^ radical taken as a reference. In this view, a lower RSE (negative, with a larger absolute value) predicts higher stability for the investigated RR′P^•^ species. Importantly, the calculation of RSE is cost-effective (compared to dimerization energies). Alternatively, the energy of the isodesmic reaction in eq 1 equals the difference between the bond dissociation energies (BDEs) of the parent PH_3_ and the given HPRR′ phosphine: RSE(RR′P^•^) = BDE(RR′P–H) – BDE(H_2_P–H). In this view, the higher stability of the radical leads to a reduction in the BDE of the RR′P-H bond.

The global stability of a given radical can be estimated by the reaction energy and Gibbs free energy of the dimerization (eq 2, Scheme 1) since this process can occur even in the absence of other reactants. The stabilization of the radical leads to increased dimerization (Gibbs free) energy. Unlike the RSE values, the dimerization Gibbs free energies are influenced by further factors besides the stability of the paramagnetic species. For example, the dimers may adopt different conformations, which would complicate the estimation of the stability. Furthermore, secondary interactions arising between the two (previously monomeric) fragments clearly affect the total stability of the dimer.^45^ As these interactions are of special importance for bulky groups, the dimerization (Gibbs free) energies are essential to address the stability of radicals with large substituents.

Besides the RSE and dimer formation energies, further measures were also applied in our research. To quantify the delocalization of the lone electron toward other centers, the Mulliken spin populations were determined. Further expanding this concept, various delocalization energies were also obtained to rationalize the energetic consequences of delocalization. In general, the Δ*E*_deloc_ energies were accessed using appropriate isodesmic reactions that compare the delocalized π-system with separated fragments that cannot interact with each other. As the actual reactions depend on the π-systems in question, they will be presented below.

The isodesmic reaction energies for the investigated RR′P^•^ radicals were first obtained at the ωB97X-D/6-311G** level, and the reliability of this level was tested with single-point calculations at the CCSD(T)/aug-cc-pVTZ//ωB97X-D/6-311G** level. In general, the two methods gave rather similar results, the differences between the two levels are typically in the range of 1–3 kcal·mol^–1^ (except for a few cases, where the largest deviation is 5 kcal·mol^–1^). Nevertheless, the two methods show the same tendencies. On smaller systems, the reliability of these levels of theory was further tested using the W1U composite method (for details, see Table S1 in the Supporting Information). The dimerization energies and the Gibbs free energies at 298.15 K were obtained at the ωB97X-D/6-311G** level of theory. The term “energy” in RSE, Δ*E*_dim_, or Δ*E*_deloc_ refers to electronic energy throughout this article.

### 2.2 Comparison of C- and N-Containing Prototype Systems

As it has been shown in the Section 1, the majority of P-radicals discovered previously contain heteroatoms (typically nitrogen, less commonly phosphorus, silicon, oxygen, or sulfur) to stabilize the paramagnetic species in monomeric forms. Therefore, first, we studied simple prototype systems, in which the P-center bears one or two methyl (CH_3_), amino (NH_2_), vinyl (CH=CH_2_), or imino (N=CH_2_) substituents, and the obtained RSEs and spin populations are shown in Table 1. The RSEs of the mono- and dimethyl phosphinyl radicals are −1.8 and −3.2 kcal·mol^–1^, respectively, and the spin populations of these two species are correspondingly high (≈1.00 e). Not surprisingly, one or two methyl groups attached to the P-center have little impact on the stability of the radical originating only from weak hyperconjugative interactions. Although the stabilizing effect of an amino substituent is significantly greater than that of a methyl group, a second amino group does not augment the stabilization further due to a saturation effect.^13^ The spin population values also support this observation since both ^•^PH(NH_2_) and ^•^P(NH_2_)_2_ radicals have similar spin populations around 0.84 e. Importantly, the effect of a vinyl group (RSE = −7.4 kcal·mol^–1^) is comparable to that of an NH_2_ substituent (−7.8 kcal·mol^–1^). In stark contrast to the amino groups, the vinyl ligands have an additive effect on the stability: the second π-bond further increases the stability by a substantial amount (5.9 kcal·mol^–1^). As shown in Section 1, imino groups (e.g., guanidine substituents) were proven to offer effective stabilization for synthetically accessed species (Figure 2). Surprisingly, the imino (N=CH_2_) substituents are not as stabilizing as the vinyl groups. Indeed, an imino ligand only results in a smaller stabilization with RSE = −5.4 kcal·mol^–1^ compared to a vinyl group (RSE = −7.4 kcal·mol^–1^). Similar to the vinyl groups, the effect of the imino substituent is also additive. However, according to the RSE values and spin populations, two vinyl ligands are even more effective in the stabilization (RSE = −13.3 kcal·mol^–1^) than two imino groups. Therefore, based on these model results, the most promising choice is to investigate carbon-containing systems exhibiting π-delocalization on either or both sides of the P-center.

**Table 1:** Radical Stabilization Energies (RSE) Based on Equation 1 at the ωB97X-D/6311-G** and CCSD(T)/aug-cc-pVTZ Level of Theory and Spin Populations at the P-Center of RR′P^•^ Radicals

R	R′	RSE ωB97X-D/6-311G** (kcal·mol^–1^)	RSE CCSD(T)/aug-cc-pVTZ (kcal·mol^–1^)	ρ_spin_ (e)
CH_3_	H	–1.8	–3.8	1.032
CH_3_	CH_3_	–3.2	–4.7	1.000
NH_2_	H	–7.8	–9.1	0.844
NH_2_	NH_2_	–5.1	–7.8	0.843
–CH=CH_2_	H	–7.4	–6.2	0.865
–CH=CH_2_	–CH=CH_2_	–13.3	–13.4	0.734
–N=CH_2_	H	–5.4	–6.2	0.875
–N=CH_2_	–N=CH_2_	–11.1	–11.6	0.751

**Figure 3:**
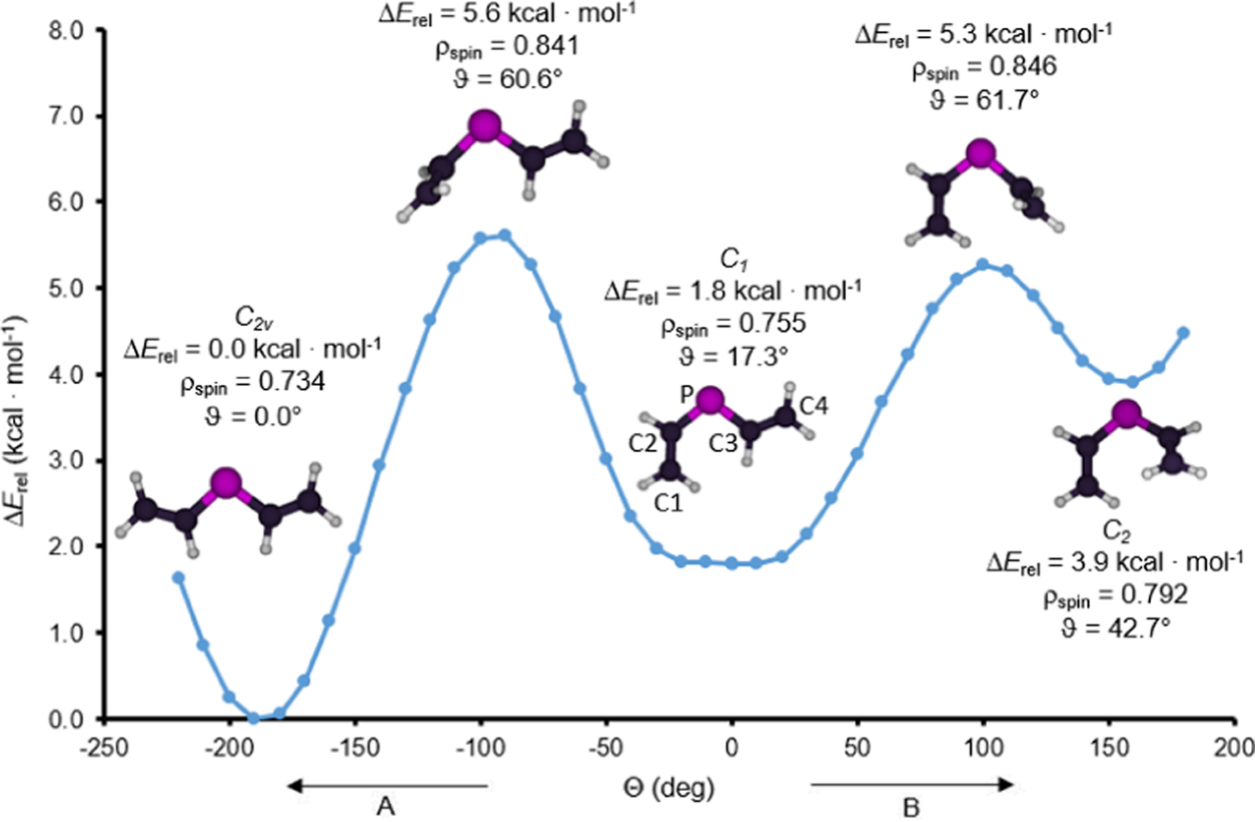
Relative energies (Δ*E*_rel_ at the ωB97X-D/6311-G** level of theory in kcal·mol^–1^ of the conformations compared to the most stable s-*trans*/s-*trans* conformation) for divinyl phosphinyl radicals plotted as the function of CPCC dihedral angles (θ in deg), starting from the asymmetric rotamer depicted in the middle (C_1_). The direction A and B show the rotation around the C2–P and P–C3 bond, respectively. The spin populations at the P-centers (ρ_spin_) and C1–C2–C3–C4 dihedral angles (ϑ in deg) are also given.

Importantly, the relative arrangement of the two vinyl groups in the divinyl phosphinyl radical should also be taken into account since this may affect the extent of delocalization via modifying the overlap of p-orbitals. To map the potential energy surface of the rotation around the P–C σ-bond, relaxed scan computations were performed: the C–P–C–C dihedral angles (θ) were separately adjusted at either side of the P-center starting from the nonsymmetric geometry (*C*_1_ in Figure 3). Altogether, three conformers were obtained (Table 2), which differ basically in the dihedral angles between the planes of the two vinyl groups (ϑ). Among them, the *C*_2*v*_ symmetric structure with two s-*trans* orientations has the lowest energy. In this rotamer, the four C-atoms and the P-center are in the same plane, and hence the p-orbitals perpendicular to this plane can overlap the most effectively. Compared to this isomer, the nonsymmetric s-*cis*/s-*trans* conformer (*C*_1_), exhibiting a dihedral angle between the two vinyl groups of ϑ = 17.3°, is 1.8 kcal·mol^–1^ less stable. In the least stable (*C*_2_ symmetric) s-*cis*/s-*cis* conformation, this torsion is even larger (ϑ = 42.7°). The larger the deviation from the planar structure is, the smaller the conjugation among the five atoms gets. This observation is also bolstered by the radical stabilization energies in Table 2 (compared to the divinyl-phosphine having the corresponding conformation), which decrease in the order *C*_2*v*_ > *C*_1_ > *C*_2_ (13.3, 10.3, and 7.7 kcal·mol^–1^, respectively). The spin populations also reflect a trend but in reversed order (0.734 e < 0.755 e < 0.792 e), indicating the highest degree of delocalization (lowest spin population) in the most stable isomer (*C*_2*v*_). Additionally, the conjugation has an effect on the bond distances as well: the larger the electronic communication is, the shorter the P–C distances and the longer the C=C bonds get.

**Table 2:** RSE, Spin Population at the P-Center, and Geometrical Parameters for Divinyl Radicals at the ωB97X-D/6-311G** level

symmetry	RSE (kcal·mol^–1^)	ρ_spin_ (e)	ϑ (deg)	*d*(P–C) (Å)	*d*(C=C) (Å)
*C*_2*v*_	–13.3	0.734	0	1.785	1.345
*C*_1_	–10.3	0.755	17.3	1.788	1.345
*C*_2_	–7.7	0.792	42.7	1.798	1.342

The structures corresponding to the transition states connecting the minima were also obtained, in which the conjugation between the 3p orbital of the P-center and one of the π-orbitals is interrupted. Consequently, the spin populations at the P-centers are larger (ρ_spin_ ≈ 0.84 e) for the transition states than for the local minima, also bolstering the reduction in conjugation.

**Scheme 2:**
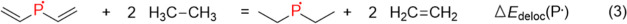
Isodesmic Reaction for Quantifying the Delocalization Energy in the Divinylphosphanyl Radical

To estimate the energetic consequences of the delocalization effects between the π-bonds and the P-center in the divinyl radical, the delocalization energy (Δ*E*_deloc_) was obtained by a suitable isodesmic reaction (eq 3) shown in Scheme 2.

The Δ*E*_deloc_ of −13.9 kcal·mol^–1^ shows significant delocalization in the whole π-system. Thus, the electronic stabilization energy arising from the conjugation between the P-center and one π-bond amounts to 7.0 kcal·mol^–1^, which is close to the rotational energy barrier (5.6 kcal·mol^–1^) obtained for the rotation of a vinyl group. This observation also shows that the delocalization energy obtained by the isodesmic reaction (eq 3, Scheme 2) provides a reasonable measure for the extent of delocalization.

### 2.3 Electronic Stabilization in Simple Carbocyclic Phosphinyl Radicals

**Figure 4:**
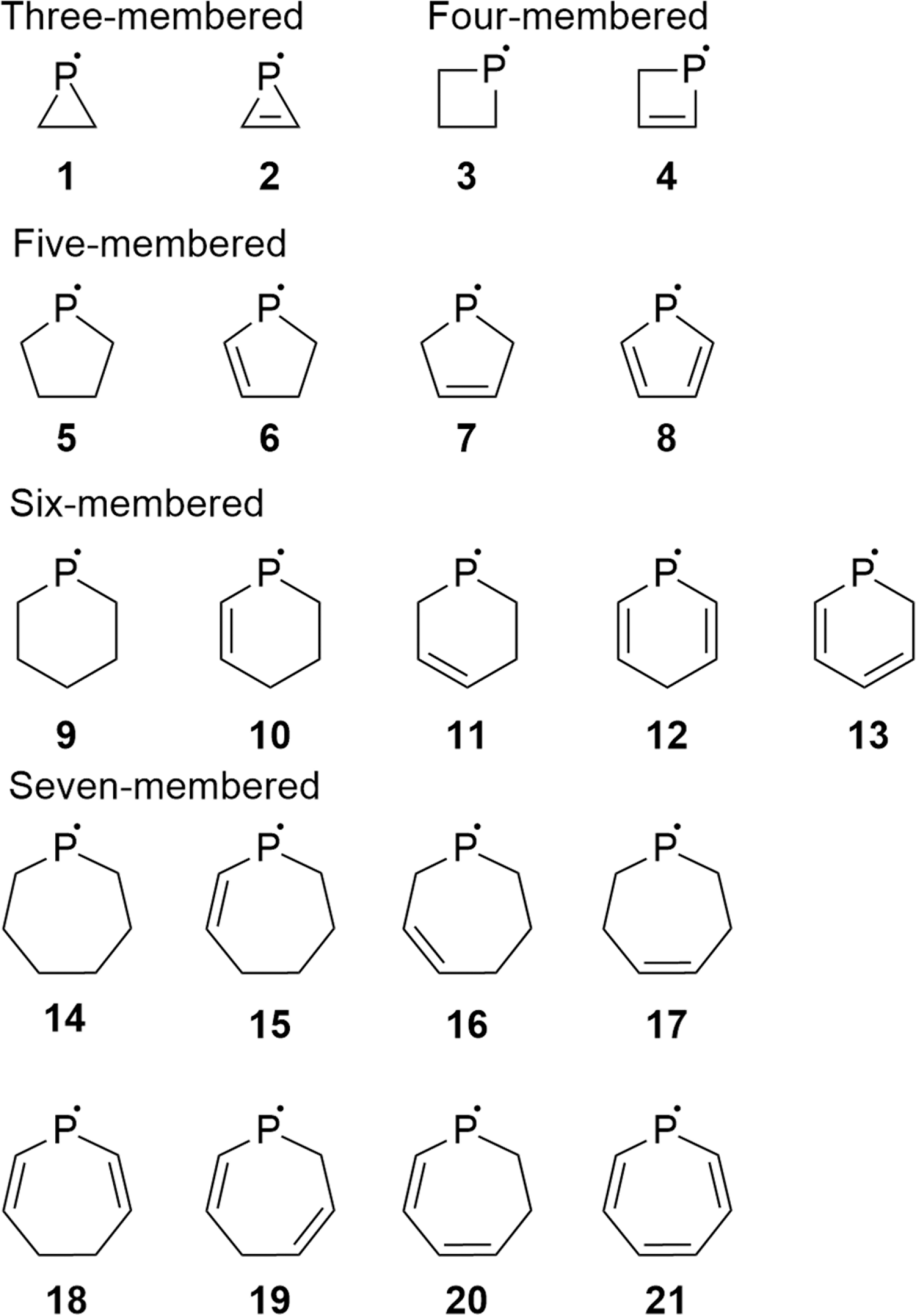
Investigated saturated rings and rings with endocyclic double bonds.

**Figure 5:**
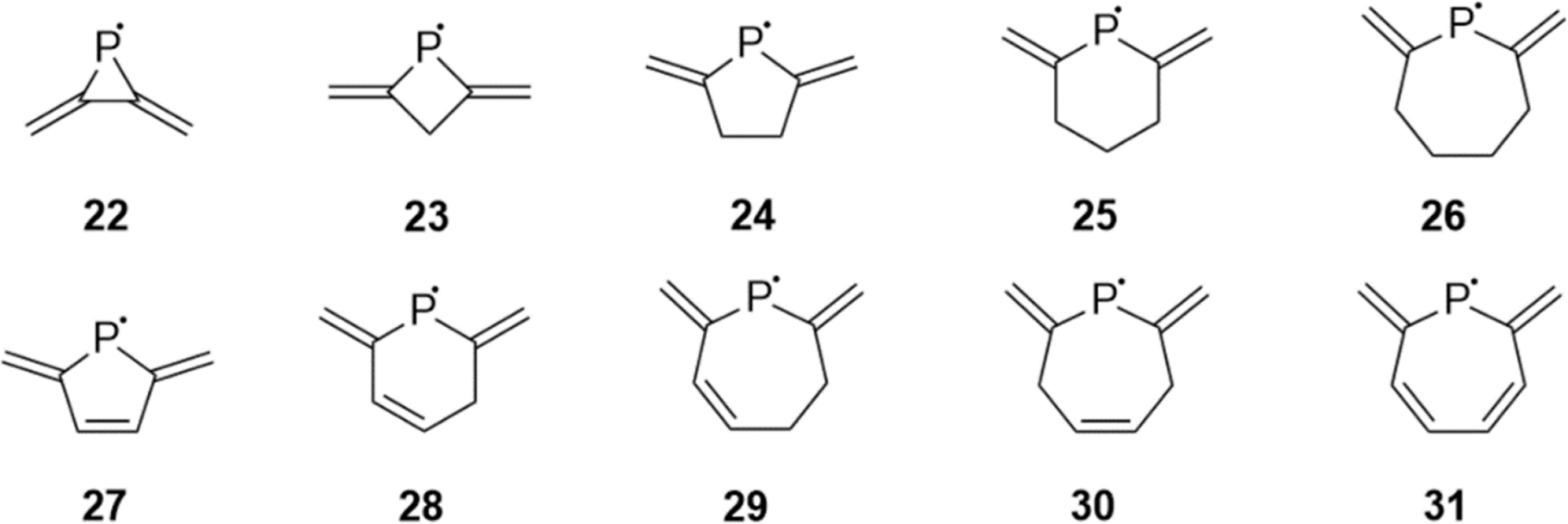
Investigated radicals with exocyclic π-bonds.

From the computations on the prototype substituents, it can be concluded that the vinyl substituents have a significant effect on the stability of the radical. Therefore, radicals containing these units are especially suitable for our investigations. Since our purpose is a systematic investigation of carbocyclic radicals, different ring sizes with saturated and variously unsaturated backbones were included as shown in Figure 4. To expand the scope of this study, we involved phosphinyl radicals with exocyclic C=C double bonds as well (Figure 5). Furthermore, the cyclic structure is of great importance to modify or constrain a given conformation,^44^ which may also influence delocalization between the P-center and the π-system.

Using the approaches presented in Section 2.1, we have obtained the RSE values. Also, we have computed the dimerization reaction energies and Gibbs free energies of these simple radicals (Table 3).

**Table 3:** RSE (at different levels of theory), Spin Population at the P-Center (ρ_spin_), Dimerization Energy (Δ*E*_dim_), and Gibbs Free Energy (Δ*G*_dim_) of the Investigated Radicals at the ωB97X-D/6-311G** Level^a^

*N*	radical	RSE ωB97X-D/6-311G** (kcal·mol^–1^)	RSE CCSD(T)/aug-cc-pVTZ (kcal·mol^–1^)	ρ_spin_ (e)	Δ*E*_dim_ (kcal·mol^–1^)	Δ*G*_dim_ (kcal·mol^–1^)
0	**1**	–4.2	–3.2	1.023	–54.3	–39.3
0	**3**	–3.7	–2.6	0.999	–55.2	–38.6
0	**5**	–4.1	–3.2	0.995	–55.0	–38.3
0	**9**	–3.9	–2.7	0.973	–56.4	–38.7
0	**14**	–3.5	–2.3	0.996	–58.5	–41.7
0	**7**	–3.7	–2.8	0.998	–57.6	–40.3
0	**11**	–1.2	–0.4	0.994	–54.7	–37.0
0	**16**	–3.2	–2.1	0.993	–57.7	–41.3
0	**17**	–3.2	–2.1	0.998	–58.0	–40.7
1	**2**	–11.8	–10.8	0.922	–49.0	–32.9
1	**4**	–8.5	–6.9	0.872	–46.8	–29.1
1	**6**	–11.0	–8.9	0.837	–48.2	–32.4
1	**10**	–8.5	–6.6	0.834	–46.0	–23.9
1	**15**	–6.6	–4.6	0.846	–49.5	–32.4
1	**19**	–8.5	–6.7	0.827	–50.2	–33.0
2	**8**	–9.3	–7.6	0.744	–45.9	–28.8
2	**12**	–14.0	–11.1	0.739	–40.3	–26.4
2	**13**	–13.6	–9.5	0.697	–48.4	–32.5
2	**18**	–11.0	–7.6	0.724	–40.0	–24.6
2	**20**	–9.2	–5.9	0.729	–44.0	–27.1
3	**21**	–11.3	–17.0	0.665	–41.4	–24.8
2	**22**	–11.8	–12.9	0.805	–43.2	–27.1
2	**23**	–13.7	–14.2	0.738	–43.6	–26.9
2	**24**	–13.2	–13.6	0.750	–43.4	–27.2
2	**25**	–11.7	–11.6	0.732	–42.6	–24.4
2	**26**	–9.4	–9.8	0.765	–44.8	–27.2
3	**27**	–12.2	–12.9	0.760	–44.4	–28.8
3	**28**	–9.7	–9.3	0.755	–44.9	–26.5
3	**29**	–8.5	–8.4	0.788	–49.2	–30.5
2	**30**	–8.2	–8.9	0.844	–50.1	–33.0
4	**31**	–8.3	–8.1	0.772	–52.5	–34.7

a The most stable conformations are taken into consideration. *N* denotes the number of the vinyl groups involved in the delocalization.

Clearly, diphosphines may adopt different conformers, and we attempted to optimize local minima starting from various initial geometries. For the symmetric radicals (including all of the saturated as well as the symmetrically unsaturated species, e.g., radicals **1**, **2**, **3**, **5**, etc.), two conformers were found: an anti and a gauche-like. However, in the case of the dimers formed from radicals having asymmetric substitution patterns (e.g., radicals **4**, **6**, **10**, etc.), basically five different conformers were located (two anti and four gauche-like, two from the latter are in meso-relation). Among these dimers, the relative energies of the different conformers vary only in a relatively narrow range (between 1 and 4 kcal·mol^–1^). Furthermore, no clear preference for either type of conformations can be observed. For clarity, we always considered the most stable conformation for calculating the dimerization energies (Table 3), and the further results are collected in the Supporting Information (SI) (Table S2 and Figure S1).

**Figure 6:**
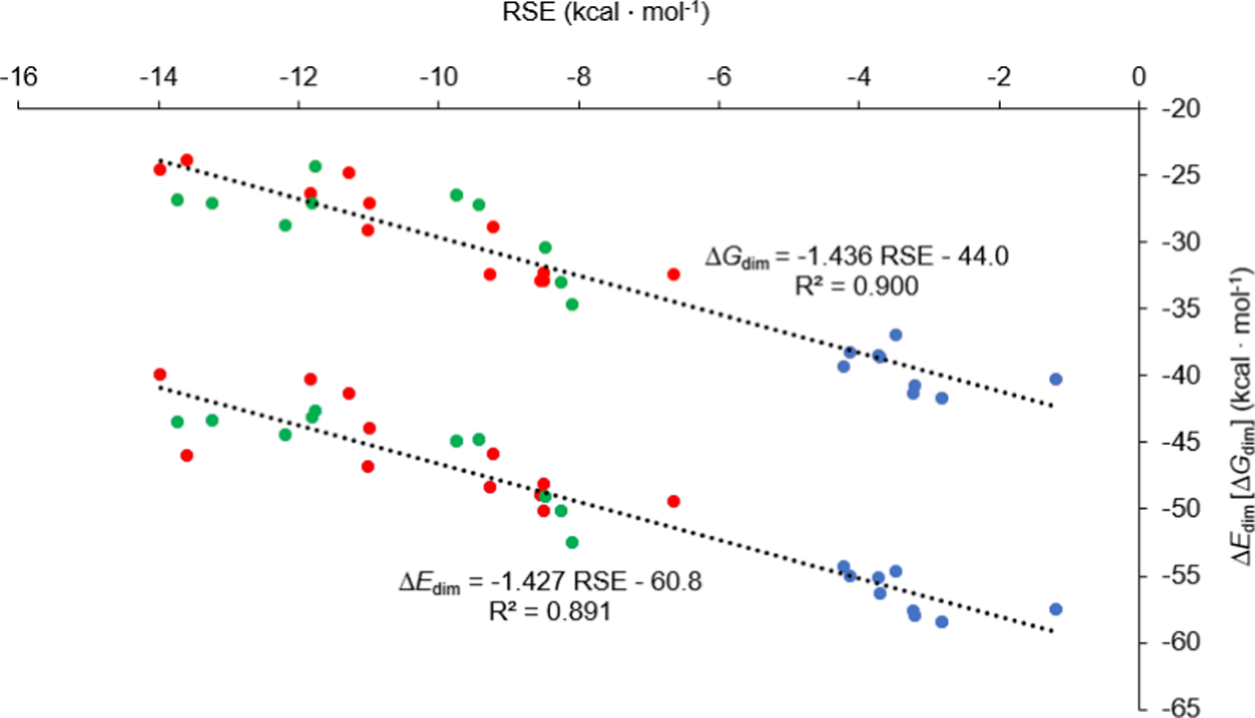
Correlation between RSEs and dimerization reaction energies or Gibbs free energies (regarding the most stable conformer of the dimers) at the ωB97X-D/6311-G** level of theory; blue: no double bond next to the P-center, red: unsaturation next to the P-center, green: exocyclic unsaturation.

**Figure 7:**
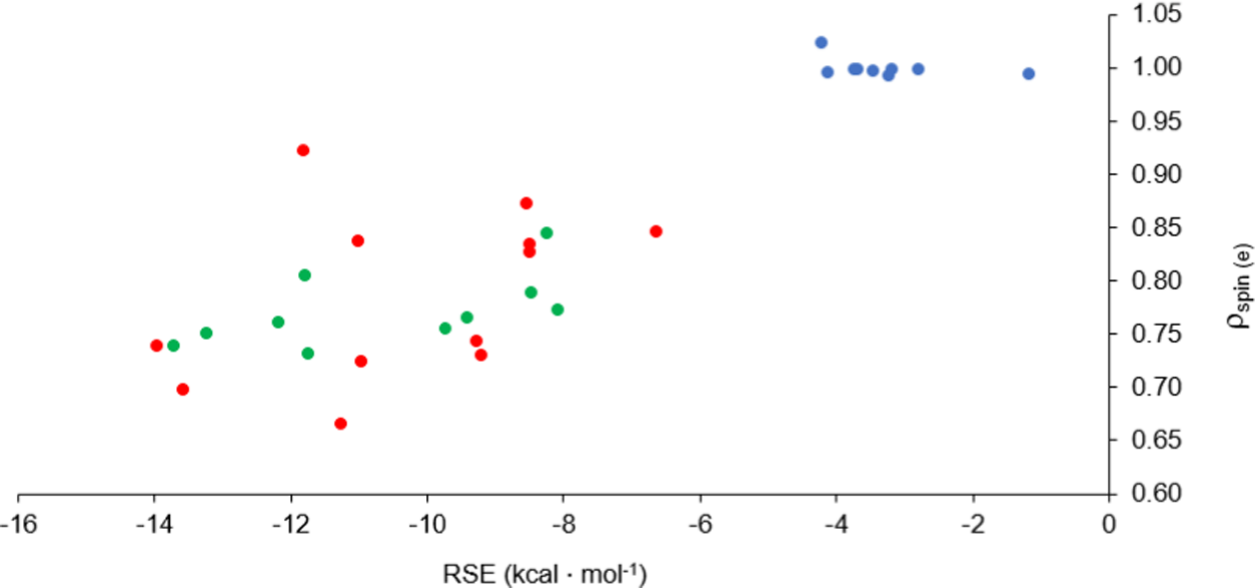
Spin populations plotted as a function of RSE at the ωB97X-D/6311-G** level of theory; blue: no double bond next to the P-center, red: unsaturation next to the P-center, green: exocyclic unsaturation.

The comparison of the RSE, dimerization (Gibbs free) energy, and spin population values leads to two important findings. First, we observe a convincing linear correlation between Δ*E*_dim_ (or Δ*G*_dim_) and the RSE values (Figure 6). Note that the bonding patterns around the tricoordinate P-centers in the dimers and in the corresponding secondary phosphines are similar because the secondary interactions only have a minor impact on the Δ*E*_dim_ of the simple radicals. As the structural changes accompanying the formation of the dimers or phosphine references are comparable, Δ*E*_dim_ and RSE values follow the same tendency. However, the RSE values provide simpler interpretations, and, in certain cases, they can be further partitioned into meaningful contributions as it will be shown below. Second, the RSE values and the spin populations show no meaningful correlation (Figure 7), only two clusters can be observed: one for the saturated ring and another (even more distributed) for the rings with unsaturation(s). In the following, the results will be discussed according to this separation.

#### 2.3.1 Saturated Rings and Rings without Unsaturation Next to the P-Center

First, we discuss the saturated systems with a number of ring atoms ranging from three to seven. The RSE values indicate that the relative stability of radicals with saturated backbones slightly increases with the ring size; however, this has an obvious limitation (the slightly more stable, but synthetically hardly achievable phosphinyl radicals with eight- or nine-membered rings are included in the SI). The P-radicals with three- and five-membered rings are slightly more stable (RSE = −4.1 and −4.2 kcal·mol^–1^, respectively) compared to those with even numbers, but no significant stabilizing interactions can be observed. Indeed, the relative stability of these radicals is similar to that of PMe_2_ (Table 1). If the ring contains unsaturation but not directly next to the P-center (**7**, **11**, **16**, **17**), the stability is practically the same as in the corresponding saturated analogues since the possible delocalization of the lone electron is inhibited by the CH_2_ groups. The dimerization reactions of saturated cyclic radicals are remarkably exothermic (Δ*E*_dim_ = −54 to −59 kcal·mol^–1^)—similar to that of the parent ^•^PH_2_-radical (−58.0 kcal·mol^–1^). The spin populations at the P-center also outline the absence of delocalization in these species (ρ_spin_ ≈ 1). Nevertheless, a carbocyclic radical without possible π-delocalization (**IV**) has been isolated as stable species, mainly due to steric effects in the α-positions (a detailed discussion of the stability of radical **IV** toward dimerization is given in Section 2.5).

#### 2.3.2 P-Radicals with One π-Bond Next to the P-Center

On the basis of the results obtained for the vinyl-substituted phosphinyl radical, the unsaturated C=C bond at either side of the P-center enables delocalization; thereby, one may expect that radicals with π-delocalization are more stable compared to the saturated analogues. The RSE values range between −6.6 and −14.0 kcal·mol^–1^. By increasing the delocalization in the rings, the relative stability of the radicals increases (the RSE values get more negative), and parallelly, the driving force for the dimerization also decreases (Δ*E*_dim_ = −40 to −48 kcal·mol^–1^). As shown above (Figure 7), the RSE and spin population values show no relationship for the family of unsaturated species, not even if the different numbers of possible π-bonds are considered separately. Therefore, a more detailed analysis of the delocalization effects is necessary for deeper understanding, and the different numbers of attached π-bonds should be separated.

First, we discuss the stability of the phosphinyl radicals with one vinyl group attached to the P-center (*N* = 1) and investigate the effect of the increasing number of methylene groups. According to the RSE values, the three- and five-membered rings (**2** and **6**, respectively) have the highest stability, while the stability shrinks with the increasing number of the CH_2_ units, e.g., for the six- and seven-membered radicals. The spin population values, however, disagree with this trend, predicting a markedly lower extent of delocalization in **2** compared to the five-, six-, and seven-membered radicals (**6**, **10**, and **15**, respectively).

On the basis of RSE values, no clear tendency can be found for the stability of these radicals. To gain a deeper understanding of the RSE values and to decipher how the introduction of unsaturation into the ring affects the stability of a given radical, we define the excess stabilization energy (ESE) as the difference between the RSE in an unsaturated radical and the corresponding saturated radical: ESE = RSE_saturated_ – RSE_unsaturated_. In this way, the change in the stabilization energy induced by a π-bond can be estimated, eliminating the effect of the ring size (Table 4).

**Table 4:** Excess Stabilization Energy (ESE, in Parentheses the Saturated Radical Used for Comparison), Delocalization Energy (Δ*E*_deloc_) of the Radicals, and the Phosphine Analogues as well as Spin Population at the P-Center (ρ_spin_) of the Radicals at the ωB97X-D/6-311G** Level^a^

unsaturated radical	ESE (kcal·mol^–1^)	*n*	Δ*E*_deloc_ (P^•^) (eq 4) (kcal·mol^–1^)	Δ*E*_deloc_ (PH) (eq 4) (kcal·mol^–1^)	ρ_spin_ (e)
**2**	7.6 (vs **1**)	0	–6.6	–14.2	0.922
**4**	4.8 (vs **3**)	1	7.1	2.3	0.872
**6**	6.9 (vs **5**)	2	10.6	3.7	0.837
**10**	4.6 (vs **9**)	3	10.3	5.7	0.834
**15**	3.1 (vs **14**)	4	10.1	6.9	0.846

a *n* is the number of CH_2_ groups in the ring according to Scheme 2.

**Scheme 3:**
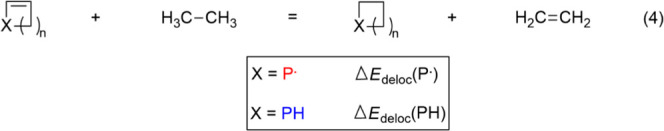
Isodesmic Reactions of the Cyclic Radicals with One Conjugating π-Bond and the Corresponding Phosphines (*n* = 0–4) for Obtaining Delocalization Energies

Contrary to the RSE or Δ*E*_dim_, the ESE values can be decomposed as ESE = Δ*E*_deloc_(P^•^) – Δ*E*_deloc_(PH) to the delocalization energies obtained separately for the radicals and the corresponding phosphine derivatives (Δ*E*_deloc_(P^•^) and Δ*E*_deloc_(PH), respectively) using the appropriate isodesmic reactions (eq 4, Scheme 3). This means that the excess stabilization energy (ESE) is governed by two main delocalization effects: the delocalization in the radical and the delocalization in the corresponding phosphine. Importantly, the energies of these reactions (Table 4) indicate that the effect of the C=C bond may be markedly different in the radicals compared to that in the corresponding phosphines. In the following, we will discuss these species in the order of increasing ring size.

In the three-membered radical **2**, the spin population at the P-center is substantial (ρ_spin_ = 0.922) outlining a significantly lower degree of delocalization compared to the five- and six-membered radicals. On the basis of the Δ*E*_deloc_(P^•^) value of −6.6 kcal·mol^–1^, the double bond has not a stabilizing but actually a destabilizing effect. In a three-membered ring, the introduction of unsaturation increases the ring strain by the distortion of the bond angles around the carbon centers. For example, the **2H** phosphirene is 17.7 kcal·mol^–1^ more strained than the saturated phosphirane **1H**.^59,60^ According to Δ*E*_deloc_, the destabilizing effect of the ring strain in radical **2** is clearly smaller (Δ*E*_deloc_ = −6.6 kcal·mol^–1^) than in the corresponding phosphine **2H** (Δ*E*_deloc_ = −14.2 kcal·mol^–1^) applied as a reference in the isodesmic reaction (eq 1, Scheme 1). This is most likely due to the delocalization of the π (C=C)-bond toward the singly occupied p-orbital at the P-center, as shown by a natural bond orbital (NBO) analysis.

The higher homologues exhibit even higher degrees of delocalization. In the four-membered radical **4**, the spin population (0.872 e) indicates a significantly smaller delocalization compared to radical**s 6**, **10**, and **15**, in correlation with the lower Δ*E*_deloc_(P^•^) value of 7.1 kcal·mol^–1^. For five-, six-, or seven-membered radicals (**6**, **10**, and **15**, respectively), the Δ*E*_deloc_(P^•^) values are around 10 kcal·mol^–1^ in nice agreement with the rather similar spin populations (0.83–0.85 e). Among these radicals, the ESE values (in contrast to the RSE) show a clear decreasing trend in the order **6** > **10** > **15**. Note that the ESE is influenced by both Δ*E*_deloc_(P^•^) and Δ*E*_deloc_(PH). Because Δ*E*_deloc_(P^•^) stays unchanged, this tendency can be explained by the changing Δ*E*_deloc_(PH) of the corresponding phosphine derivatives, which increases with the number of CH_2_ groups. According to NBO analyses on phosphines **6H**, **10H**, and **15H**, this change in the Δ*E*_deloc_(PH) is caused by the differing strength of interaction between the π*-orbitals and the lone pair of the P-atom, as well as between the π-orbitals and the P–H σ*-bond. Therefore, the varying stabilities of radicals **6**, **10**, and **15** are ruled by the differing delocalization effects in the phosphine references.

#### 2.3.3 P-Radicals with Two or Three π-Bonds in the Delocalization

In the following, the effect of more extended delocalization at the P-center will be discussed. By increasing the unsaturation, the extent of the delocalization and therefore the relative stability can be influenced. To decouple the effect of unsaturation from the ring size, we again obtained the ESE values (see Table 5).

**Table 5:** Excess Stabilization Energy (ESE, in Parentheses the Saturated Radical Used for Comparison), Delocalization Energy (Δ*E*_deloc_) of the Radicals, and the Phosphine Analogues Spin Population at the P-Center (ρ_spin_) for the Radicals at the ωB97X-D/6-311G** Level of Theory^a^

unsaturated radical	ESE (kcal·mol^–1^)	*n*	Δ*E*_deloc_(P) (Scheme 4) (kcal·mol^–1^)	Δ*E*_deloc_(PH) (Scheme 4) (kcal·mol^–1^)	ρ_spin_ (e)
**8**	5.2 (vs **5**)	0	20.7 (eq 5)	15.5 (eq 5)	0.744
**12**	10.1 (vs **9**)	1	20.1 (eq 5)	10.0 (eq 5)	0.739
**13**	9.7 (vs **9**)	1	20.2 (eq 6)	10.5 (eq 6)	0.697
**18**	8.5 (vs **14**)	2	19.1 (eq 5)	11.6 (eq 5)	0.724
**20**	5.8 (vs **14**)	2	21.1 (eq 6)	15.3 (eq 6)	0.729
**21**	7.8 (vs **14**)	3	31.5 (eq 7)	23.7 (eq 7)	0.665

a *n* is the number of CH_2_ groups in the ring according to Scheme 4.

Similar to the procedure demonstrated above, the ESE values were decomposed to delocalization energies obtained separately for the radicals and the corresponding phosphines (Δ*E*_deloc_(P^•^) and Δ*E*_deloc_(PH), respectively) using the isodesmic reactions (eqs 5–7) shown in Scheme 4, which quantify the total delocalization in the whole π-system. Again, the discussion follows the growing ring size, and the main goal is to give explanations to the different RSE trends.

**Scheme 4:**
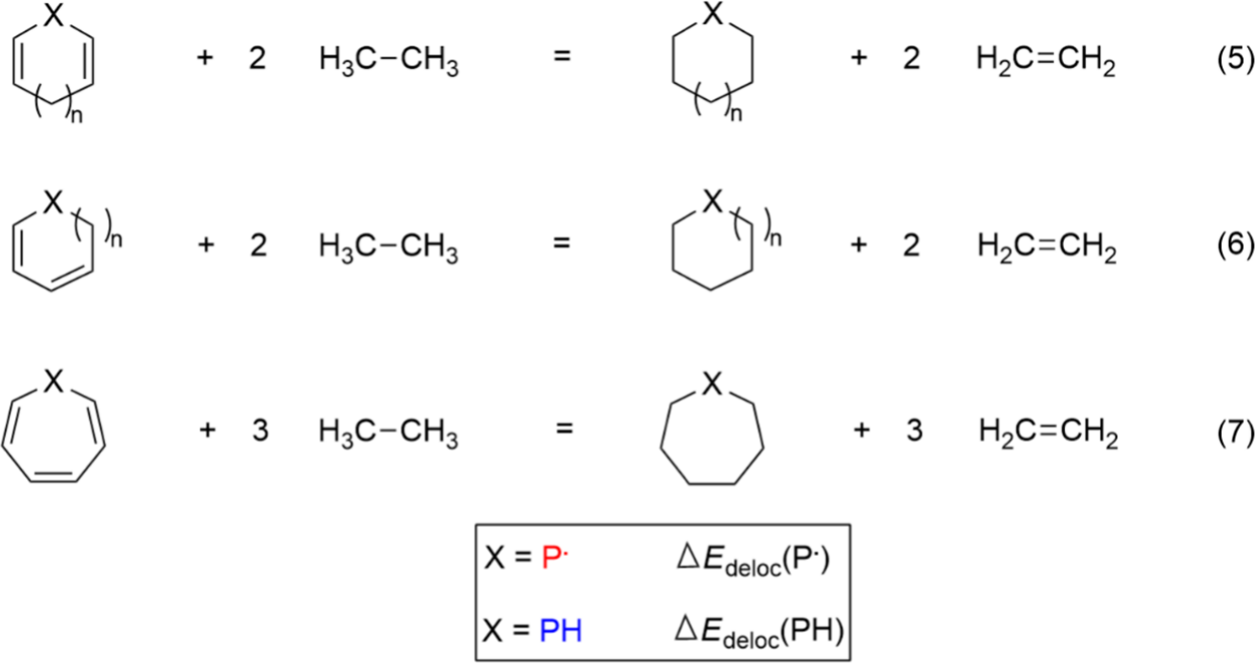
Isodesmic Reactions for Cyclic Radicals with Larger π-Conjugation and the Corresponding Phosphines (*n* = 0–2)

The RSE of the five-membered radical **8** (−9.3 kcal·mol^–1^, Table 4) indicates rather high relative stability. Although the ESE of this radical is only modest (5.2 kcal·mol^–1^), the spin population of 0.744 e predicts a rather high extent of delocalization. Indeed, the delocalization in the π-system is not only substantial for radical **8** (Δ*E*_deloc_(P^•^) = 20.7 kcal·mol^–1^) but also for the corresponding phosphine **8H** (Δ*E*_deloc_(PH) = 15.5 kcal·mol^–1^). Hence, these similar delocalization energies only lead to a moderate ESE. Note that in these species, in contrast to the larger rings, the two C=C π-bonds are also directly connected with each other, and thus, Δ*E*_deloc_ measures the total delocalization, including that between the two double bonds.

For the six-membered radicals **12** and **13**, the RSE values are practically the same (−14.0 and −13.6 kcal·mol^–1^, respectively) and show remarkable stability compared to other investigated radicals. The Δ*E*_deloc_(P^•^) values are also large, approaching 20 kcal·mol^–1^. Interestingly, they are independent of whether the P-center is between the two double bonds (−C=C–*P̀*–C=C–, in **12**) or located terminally (C=C–C=C–P, in **13**) in the 5π delocalization. The higher ESE (and thus RSE) values of **12** and **13** compared to the five-membered **8** can be attributed to the lower extent of delocalization (Δ*E*_deloc_(PH) ≈ 10 kcal·mol^–1^) in the corresponding phosphine analogues (**12H** and **13H**) compared to **8H**.

In the seven-membered radicals **18** and **20**, the extent of the delocalization is similar (Δ*E*_deloc_(P^•^) = 19.1 and 21.1 kcal·mol^–1^), and is comparable to those in **8**, **12**, and **13**, in line with the similar spin population values (around 0.7 e). However, the delocalization in the corresponding phosphines **18H** and **20H** is different (Δ*E*_deloc_(PH) = 11.6 and 15.3 kcal·mol^–1^, respectively), which is also reflected in the differing ESE and RSE values.

Remarkably, in the case of radical **21** exhibiting three conjugated double bonds in the ring, the Δ*E*_deloc_(P^•^) of 31.5 kcal·mol^–1^ is even larger than for all of the other species, owing to the more extended delocalization. Additionally, the spin population at the P-center is clearly the lowest (0.665 e), indicating the highest delocalization degree among the investigated radicals.

**Figure 8:**
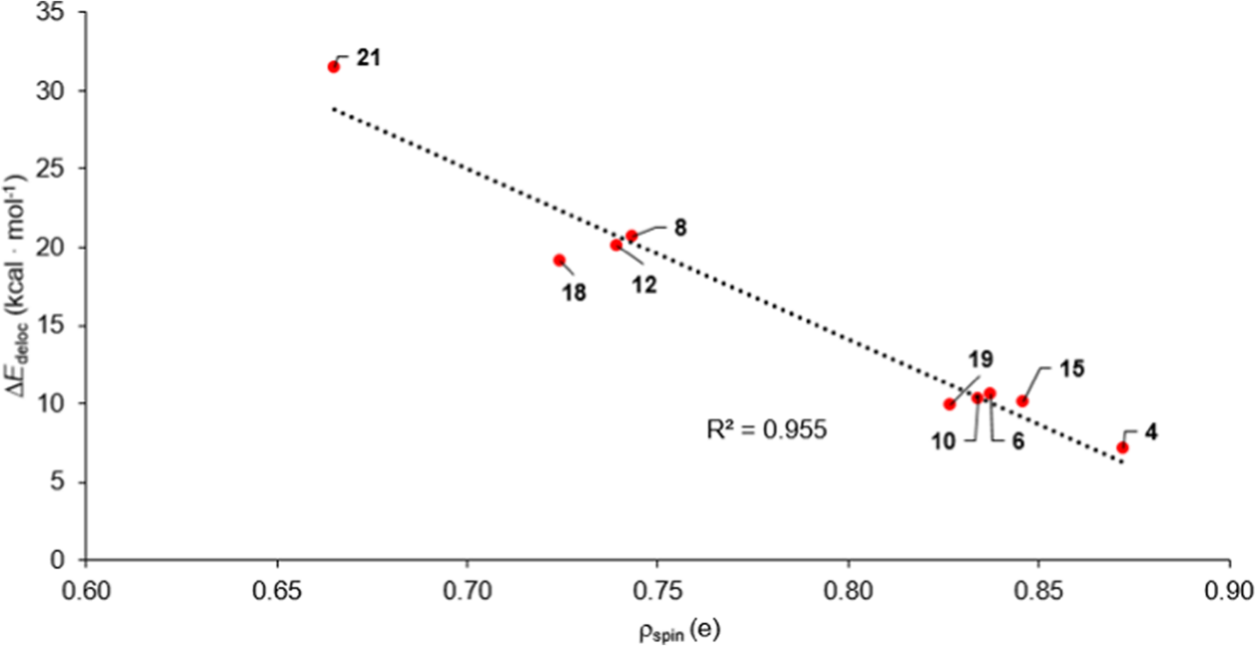
Correlation between spin population and the delocalization energy of the radicals at the ωB97X-D/6311-G** level of theory.

On the basis of the calculations on species containing one, two, and three C=C π-bonds in the conjugation, we have found a linear correlation between the delocalization energy (Δ*E*_deloc_(P^•^), Schemes 3 and 4) and the spin population at the P-center (Figure 8). Importantly, Δ*E*_deloc_(P^•^) assesses the total interaction in the whole π-system and not exclusively the communication between the P-center and the π-bonds. Hence, it is remarkable that the additional π-bonds being further from the P-center also have an impact on the delocalization of the radical center. Correspondingly, the formal addition of a further π-bond to the conjugation results in an increase of 10 kcal·mol^–1^ in Δ*E*_deloc_(P^•^) (two and three vinyl groups lead to Δ*E*_deloc_ around 20 and 30 kcal·mol^–1^, respectively). An additive but decreasing trend can be seen for ρ_spin_; expanding the π-system with a further double bond leads to a systematic depletion by 0.1 e in the spin population at the P-center. Thus, we may conclude that the spin population correlates only with the delocalization energy, but the stability of the radical in terms of RSE is influenced by further factors such as delocalization in the reference phosphine species.

#### 2.3.4 Radicals with Exocyclic π-Bonds

As the communication between the P-center and the π-bonds is not necessarily endocyclic, we have also explored the possibility of unsaturations being exocyclic with respect to the ring (Figure 5).

These species can be divided into two larger groups depending on the bridging backbone connecting the two C=C bonds: (i) those with saturated backbones and (ii) those containing additional unsaturation(s). The “exocyclic” systems with saturated backbone can be considered as radicals exhibiting two vinyl groups connected by different numbers of CH_2_ units. The radical stabilization energies (RSE) obtained by eq 1 indicate rather similar relative stability for radicals **22** to **29** in the range of 9.4–13.7 kcal·mol^–1^ (Table 3). These values are close to the RSE of divinyl phosphinyl radical (7.7–13.3 kcal·mol^–1^ in different conformations, vide supra) and that of the endocyclic radicals with two conjugated π-bonds. Furthermore, the RSE depends only moderately on the number of connecting atoms in the ring. The lowest and highest stabilization according to RSE was obtained for the seven-membered **26** and four-membered **23**, respectively, but the five-membered **24** radical also has remarkable stability.

**Scheme 5:**
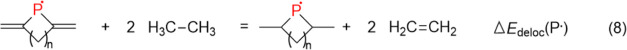
Isodesmic Reaction for the Saturated Exocyclic Systems (*n* = 0–4)

We have again obtained the delocalization energies using the isodesmic reaction (eq 8, Scheme 5) with respect to the corresponding saturated analogues. According to the Δ*E*_deloc_ values (Table 6) the extent of the delocalization in the radicals with exocyclic C=C bonds is somewhat lower (<20 kcal·mol^–1^, except for **27**) than in the analogues with endocyclic unsaturations, but it is larger than the divinyl phosphinyl radical (13.9 kcal·mol^–1^, eq 3, Scheme 2). As an exception, in the three-membered radical **22**, practically no delocalization energy can be obtained. It is known that the exocyclic C=C bond results in a significant destabilization of the three-membered heterocycles.^61^ Thus, the deminishing Δ*E*_deloc_ for radical **22** is most likely the result of the opposing effects of stabilizing π-delocalization (as supported by an NBO analysis) and destabilizing strain. Compared to radical **2**, the delocalization energy is remarkably larger for the four-, five-, six-, and seven-membered exocyclic radicals with saturated bridges (**23**–**26**). These results are further supported by the spin population values (for **22** a higher value of 0.805 e, and for **23**–**26**, similarly low values around 0.73–0.77 e, indicating effective delocalization).

**Table 6:** C=C···C=C Dihedral Angle (ϑ), Δ*E*_deloc_, and Spin Population at the P-Center for Exocyclic Systems at the ωB97X-D/6-311G** Level of Theory

radical	ϑ (deg)	Δ*E*_deloc_(P^•^) (eq 4) (kcal·mol^–1^)	ρ_spin_ (e)
**22**	0.0	–0.39	0.805
**23**	0.2	15.1	0.738
**24**	68.9	17.6	0.750
**25**	0.0	16.1	0.732
**26**	53.8	18.0	0.765
**27**	0.1	24.3	0.760
**28**	32.2	19.6	0.755
**29**	64.5	18.2	0.788
**30**	97.0	15.0	0.844
**31**	15.8	17.2	0.772

Surprisingly, by intensifying the extent of possible delocalization through the introduction of further unsaturations into the π-systems (e.g., **24** vs **27** or **25** vs **31**), the RSE values do not improve remarkably, and in most cases, they even predict lower stability compared to those with saturated backbones. The Δ*E*_deloc_ values for **27** to **31** are mostly below 20 kcal·mol^–1^, showing a saturation. The spin populations are also in the range of 0.76–0.84 e, indicating quite similar degrees of delocalization (Table 6). These observations contradict the expectations based on the endocyclic species, where the delocalization increases additively with the number of unsaturations. The most likely explanation for this discrepancy is that in the exocyclic cases, the introduction of a further π-bond next to the linear π–P^•^–π system results in a competition due to cross-conjugation instead of a possible synergistic effect induced by extended conjugation. Additionally, the conjugation is more feasible between two C=C π-bonds than between the C=C π-bond and a considerably larger 3p orbital at the P-center. Therefore, the RSEs and spin populations of the exocyclic type of radicals with unsaturated backbones (e.g., **27**) indicate even lower stabilization compared to those with saturated backbones (e.g., **24**).

As an exception, the seven-membered exocyclic radical **30** lies out from the trend of Δ*E*_deloc_ and spin populations. In this case, the double bond in the backbone of the ring distorts the geometry of the radical significantly from planar (ϑ = 97.0°). Therefore, the C–P–C=C dihedral angle is 120°, so the exocyclic C=C π-bond cannot overlap effectively with the 3p orbital of the P-center. This restricted delocalization can also be bolstered by the rather high spin population (ρ_spin_ = 0.844 e). Thus, we may conclude that in cyclic radicals, not only the number and location of the unsaturation but also the geometrical aspects are of importance.

### 2.4 From Model Species toward Feasible Systems

The correlations in Figure 6 can serve for estimation of the optimal RSE value that is needed to hamper the dimerization. If the dimerization energy is positive, and thus the formation of the P–P bond is thermodynamically disfavored, the radicals can be considered stable against dimerization. To reach Δ*E*_dim_ = 0 by extrapolation of the RSE values, at least RSE = −38.5 kcal·mol^–1^ stabilization is necessary (which would mean −28.5 kcal·mol^–1^ in the case of Δ*G*_dim_). Clearly, according to our calculations, these criteria cannot be achieved exclusively with electronic effects, and consequently, steric congestion is also essential.

The selection of phosphinyl radicals for exploring the substituent effects was based on two criteria: (a) the radical should have excellent relative stability according to the RSE value and (b) synthetic methods that are straightforward and substituent-tolerant should be available for constructing the phosphorus heterocycles that may serve as precursors to the radicals. To meet these criteria, we have chosen the radicals **2**, **8**, **12**, and **21** for further studies. Indeed, all of these radicals have remarkable RSE values, and the precursors to these radicals, namely, phosphirenes,^62^ phospholes,^63−65^ phosphinines^66,67^ (phosphabenzenes), and phosphepins,^68^ can be synthesized using well-established and effective techniques.

**Figure 9:**
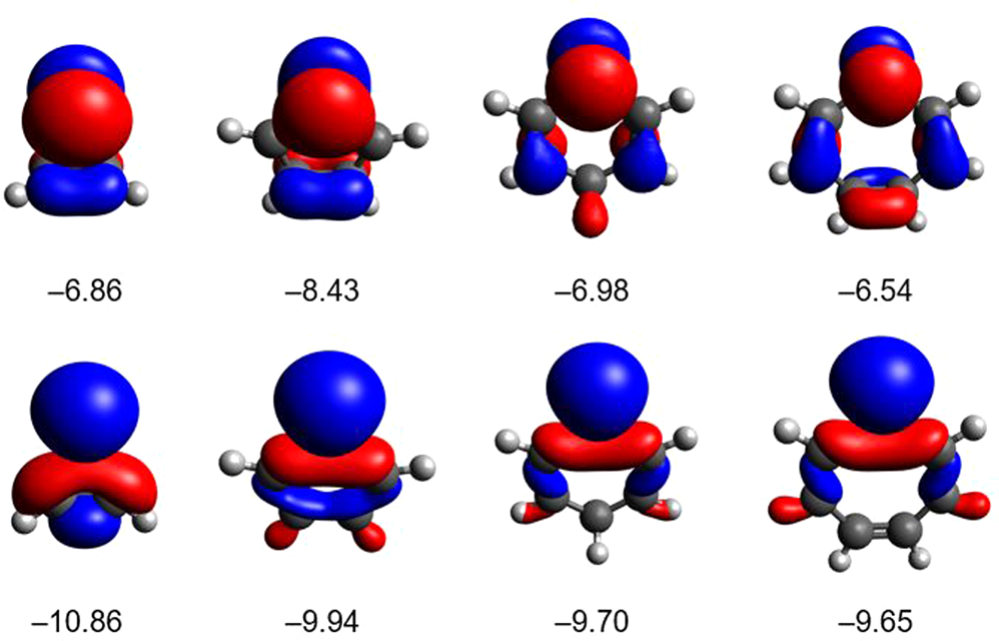
Kohn–Sham orbitals of radicals **2**, **8**, **12**, and **21** (from left to right, at the ωB97X-D/6311-G** level of theory, isovalue of 0.05). Top: singly occupied molecular orbital (SOMO) of the π-system. Bottom: in-plane lone pair at the P-center. Orbital energies are given in eV.

To gain further insights into the structures and electronic structures of these simple radicals, in the following, we briefly discuss their selected geometrical parameters (Table S3), Kohn–Sham orbitals (Figure 9), and relevant results from NBO analyses.

All of the four radicals belong to the *C*_2*v*_ point group, and the PC*_n_* cores are planar. The P–C bond distances (Table S3) indicate single bonds, but they decrease in the order **2** > **8** > **12** > **21**. The P–C bond length in the strained three-membered radical **2** (1.818 Å) significantly exceeds those in other examples (1.788 Å in **8** to 1.780 Å in **21**). The C–C bond length is the shortest in species **2**, and it shows alternations between single and double bonds in the other congeners.

The tendency describing the gradual strengthening of the P–C bonds follows the declining trend of the spin populations, again indicating the extension of conjugation of the lone electron with the π-system in the same order.

The amplifying conjugation can also be nicely perceived on the SOMO orbitals (Figure 9). All of these orbitals represent antibonding combinations of the 3p*_z_* atomic orbitals at the P-centers (as major contributors) with the π-systems of the carbon backbones. As a consequence of the antibonding characters, the energy levels belonging to these SOMOs are destabilized compared to that in the parent PH_2_ radical (ε_SOMO_ = −8.97 eV).

In contrast to the π*-type SOMO orbitals, the energies of the in-plane lone pairs at the P-centers show little variation and are rather similar to that of the PH_2_ species (ε = −10.70 eV).

According to NBO analyses, as expected, the natural orbitals corresponding to these in-plane pairs exhibit dominant s-characters. However, the s-contribution is larger for the strained species **2** (83.1%) than for the remaining cases (66.3, 65.0, and 63.8% in **8**, **12**, and **21**, respectively). The increased s-character of the lone pair in radical **2** is in accordance with similar observations on three-membered rings containing pnictogens.^60^

### 2.5 Steric Effects

**Figure 10:**
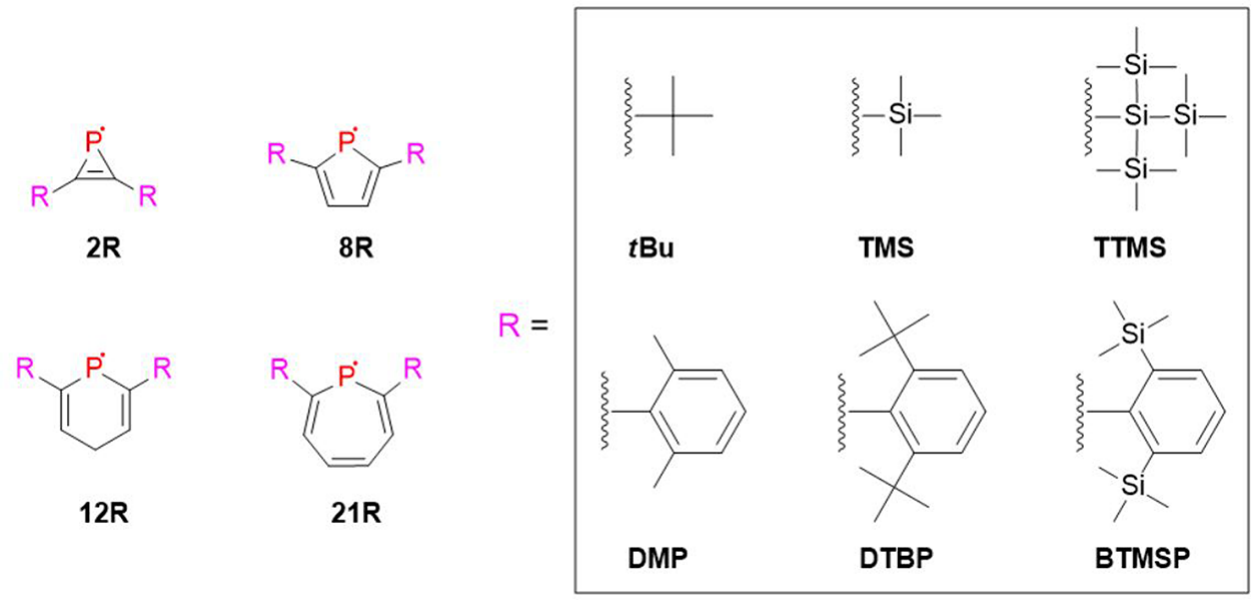
Utilized sterically demanding substituents [R = *t*Bu: *tert*-butyl; TMS: trimethylsilyl; TTMS = tris(trimethylsilyl)silyl; DMP = 2,6-dimethyl-phenyl; DTBP = 2,6-di-*tert*-butylphenyl; BTMSP = 2,6-bis(trimethylsilyl)-phenyl] on selected radicals (**2R**, **8R**, **12R**, and **21R**).

Since our target is to find synthetically accessible species, besides maximizing the electronic interactions, further stabilization via bulky substituents must also be considered. As discussed in the previous section, we selected four heterocyclic species, that is, radicals **2**, **8**, **12**, and **21**, at their α-C-atoms we introduced sterically encumbered substituents that are commonly employed in synthetic studies: without claiming completeness, the *tert*-butyl, trimethylsilyl, supersilyl [Si(SiMe_3_)_3_], 2,6-dimethyl-phenyl, 2,6-di-*tert*-butylphenyl, and 2,6-bis(trimethylsilyl)-phenyl groups were selected (Figure 10). The calculated dimerization energies and Gibbs free energies (Table 7) show remarkable variation in the ring size and substituents.

**Table 7:** Energies and Gibbs Free Energies (in Italics) of the Dimerization Reactions with Different Substituents at the ωB97X-D/6-311G** Level^a^

substituents (R)	**2R**	**8R**	**12R**	**21R**
*t*Bu	–44.6/*–25.1*	–53.6/*–35.1*	–43.0/*–22.7*	–63.5/*–42.7*
TMS	–45.9/*–32.4*	–58.4/*–36.2*	–47.0/*–24.2*	–60.0/*–39.7*
TTMS	–29.7/*–20.6*	–49.2/*–14.2*	–43.6/*–6.6*	–20.5/*11.4*
DMP	–49.4/*–32.4*	–62.0/*–35.8*	–45.2/*–21.2*	–48.2/*–21.8*
DTBP	–25.5/*–9.7*	–29.7/*–1.0*	10.5 [9.0]/*35.7*	8.0 [9.0]/*37.5*
BTMSP	–39.3/*–19.7*	–28.4/*–13.2*	0.8 [−3.3]/*29.4*	1.6 [0.7]/*31.2*

a *t*Bu: *tert*-butyl; TMS: trimethylsilyl; TTMS = tris(trimethylsilyl)silyl; DMP = 2,6-dimethyl-phenyl; DTBP = 2,6-di-*tert*-butylphenyl; BTMSP = 2,6-bis(trimethylsilyl)-phenyl, LNO-CCSD(T)/CBS dimerization energies in brackets.

Prior to discussing the obtained results, important arguments on the Gibbs free energy need to be clarified. To eliminate the thermodynamic force for the dimer formation, Δ*G*_dim_ > 0 is in principle a sufficient criterion. For association/dissociation reactions, however, the calculation of Gibbs free energies is less reliable. This is partially due to the approximations applied for calculating the entropy terms. Furthermore, the gas-phase calculations tend to overestimate the entropy factor. Indeed, our computations refer to isolated molecules in vacuum, while for the reactions conducted in solutions, the solvent molecules have an impact on the molecular motions, and thereby, the entropy is reduced compared to the calculated values.^55^ Because of these uncertainties, and as the dimerization (electronic) energy (Δ*E*_dim_) gives a stricter criterion than the Δ*G*_dim_, we will quest for radicals with positive dimerization energy (Δ*E*_dim_ > 0).

Using the sterically less demanding *tert*-butyl, trimethylsilyl, dimethyl-phenyl, and supersilyl groups, all of the investigated radicals are expected to dimerize since the dimerization reactions are significantly exothermic, but these dimerization energies are already less substantial than those of the corresponding radicals with H substituents. In stark contrast to the less demanding substituents, using the even more sterically congesting di-*tert*-butylphenyl and bis(trimethylsilyl)-phenyl groups at the α-C-atoms of the six- and seven-membered unsaturated cyclic radicals (**12**, **21**), positive dimerization energies were obtained leading to significantly endergonic dimerization (Δ*G*_dim_ > 30 kcal·mol^–1^). The dimerization energies of these four radicals were further studied using high-level LNO-CCSD(T) calculations [extrapolated to the complete basis set (CBS) limit], which show nice agreement with the DFT results (for details, see Section 4). As an additional benchmark to our calculations, the dimerization energy of radical **IV** in Figure 1 was also reckoned, and the Δ*E*_dim_ value of 11.5 kcal·mol^–1^ is in accordance with the experimentally observed remarkable stability of this species. Altogether, we may conclude that radicals **12DTBP**, **12BTMSP**, **21DTBP**, and **21BTMSP** can be promising synthetic targets.

According to Table 8, the P–P bond distances in the dimers corresponding to the investigated radicals are elongated compared to that in H_2_P–PH_2_ (2.245 Å) and are similar to those reported previously for amino substituted diphosphines.^55^ On the basis of our model calculations, the expansion of the P–P bond in H_2_P–PH_2_ to the largest distance found in the dimer of **21BTMSP** of 2.378 Å only requires 2.1 kcal·mol^–1^ energy. Therefore, these P–P bond lengths do not reflect the intrinsic strength of the P–P bond and are influenced by minor effects.

**Table 8:** Contributions to Δ*E*_dim_: Δ*E*_prep_, Δ*E*_int_, Δ*E*_disp_ (kcal·mol^–1^), and P–P Bond Distances (Å) in the Dimers of **12DTBP**, **12BTMSP**, **21DTBP**, **21BTMSP**, and **IV** at the ωB97X-D/6-311G** Level of Theory

radical	Δ*E*_dim_ (kcal·mol^–1^)	Δ*E*_prep_ (kcal·mol^–1^)	Δ*E*_int_ (kcal·mol^–1^)	Δ*E*_disp_ (kcal·mol^–1^)	P–P distance (Å)
**12DTBP**	10.5	69.5	–59.0	–56.8	2.307
**12BTMSP**	0.8	67.3	–66.5	–56.8	2.362
**21DTBP**	8.0	70.9	–62.9	–61.7	2.359
**21BTMSP**	1.6	67.6	–66.0	–58.2	2.378
**IV**	11.5	47.9	–26.4	–44.8	2.285

To shed light on the interactions that affect the dimerization of radicals **12R** and **21R** (R = DTBP, BTMSP) and **IV**, the dimerization energies were decomposed to meaningful terms as Δ*E*_dim_ = Δ*E*_prep_ + Δ*E*_int_. The preparation energy (Δ*E*_prep_, also called deformation energy) accounts for the invested energy that is needed to transform the geometries of the monomeric radicals to adapt those in the dimers. The interaction energy (Δ*E*_int_) is the energy released upon the formation of a dimer from the monomers having distorted geometries. The magnitude of London dispersion forces contributing to the interaction energy was estimated by dispersion terms (Δ*E*_disp_).^55^

The preparation energies for the radicals **12R** and **21R** are rather similar and are significantly larger than that calculated for species **IV**. This difference can be explained by the differing rigidity of the two types of radicals. Indeed, the six- and seven-membered rings having extended π-conjugations undergo more substantial reorganization compared to radical **IV** with a flexible saturated methylene backbone (Figures S2 and S3). Additionally, this increase in the preparation energy of approximately 20 kcal·mol^–1^ matches the range of extra stabilization that heightens the dimerization energies of radicals **12** or **21** compared to that of **5** (ΔΔ*E*_dim_ = 14.7 and 13.6 kcal·mol^–1^, respectively, Table 3). These results led us to conclude that the interruption of the delocalized π-system and the significant geometrical and conformational reorganization play a notable role in stabilizing the radicals **12** and **21**.

The interaction energies also show a distinction between the radical **IV** and the species with π-conjugations: the latter group has more substantial Δ*E*_int_ values. On the basis of Δ*E*_disp_, the major contribution to the interaction energy stems from dispersion forces. The lower (more substantial) Δ*E*_int_ and Δ*E*_disp_ values corresponding to species **12R** and **21R** can be explained by the presence of additional aryl rings that may build up more extended networks of weak interactions, stabilizing further the dimeric forms.

## 3 Conclusions

A large portion of stable or persistent phosphinyl radicals obtained experimentally so far is acyclic and typically contains at least one but often more heteroatoms (typically N, P, S, Si, or even transition metals). Carbocyclic P-radicals are much less explored although the C=C double bonds may offer substantial electronic stabilization. To better understand the stabilizing effects and their dependency on the ring size, we carried out systematic investigations on saturated and unsaturated rings (Figure 4). We have computed the reaction energies of hypothetical isodesmic H-transfer reactions to measure the radical stabilization energy (RSE) as well as we have obtained the dimerization energies of the radicals. Importantly, in the absence of sterically demanding substituents, the RSE values correlate with the dimerization (Gibbs free) energies. Thus, the more easily obtainable RSE values can be used to predict conveniently the stability trends.

If the P-radical center is between two sp^3^ C-atoms (radicals with saturated backbones), the relative stabilization based on the RSE values is minor such as in the case of dimethyl phosphanyl radical and is attributable to negligible hyperconjugation effects. However, substantial electronic stabilization approaching RSE = 17 kcal·mol^–1^ can be achieved by introducing C=C units next to the P-center into the ring leading to effective conjugation. Aiming for a comprehensive survey, we have also investigated radicals containing different numbers of unsaturations. The radical stabilization energies (RSEs) show no correlation with the spin populations (which aim to account for the delocalization of the lone electron with the π-system). The reason for this discrepancy is that the RSE depends on the delocalization effects in both the radical and the corresponding phosphine reference. To clarify this, the extent of delocalization was estimated as delocalization energy (Δ*E*_deloc_) separately for the radicals and their phosphine analogues. Unlike the RSE values, the Δ*E*_deloc_ values of the radicals correlate with the spin populations, allowing for a deeper understanding of the delocalization effects influencing the RSE values. Importantly, an additivity phenomenon was also observed: the more π-bonds interact with the P-center in the conjugation, the larger the achievable delocalization energy. Furthermore, the location of the P-center in a more extended conjugated π-system (e.g., central or terminal) has a negligible effect on the energetic consequences of the delocalization.

As the position of the double bond is not necessarily endocyclic, P-radicals with two exocyclic C=C bonds were also investigated. The calculated RSE values of these species are similar to those observed for the divinyl phosphinyl radicals and for the analogous radicals with endocyclic C=C bonds. However, by extending the delocalization, no significant improvement can be realized. In certain cases, where both endo- and exocyclic types of π-bonds are present, even slight destabilization was observed, due to cross-conjugation, in line with the trend of the spin populations.

According to the correlation between RSEs and the computed dimerization reaction energies, phosphinyl radicals stabilized only with electronic effects cannot be considered thermodynamically stable (highly exothermic and exergonic dimerization, Δ*E*_dim_ and Δ*G*_dim_ ≪ 0). However, by selecting appropriate sterically demanding substituents on the α-C-atoms, the dimerization can be hampered. Indeed, we have found promising carbocyclic phosphinyl radicals that are expected to be realized synthetically in the future as stable compounds. Furthermore, we have shown that the rigid structures of the P-radicals exhibiting distinct delocalizations play an important role in prohibiting the formation of dimers.

It is important to emphasize that from a large number of species, only a handful seem to be suitable for synthetic studies. This reflects clearly how challenging is to stabilize P-centered radicals, which is partially due to the substantial strength of the P–P bond. Additionally, if bulky substituents are employed to protect the reactive P-center, substantial dispersion interaction arises between the fragments, acting counterproductively to stabilize the dimeric form. We have shown, however, that systematic computational investigations are vital for screening a large group of candidates prior to expensive experiments.

Finally, we have presented that stable carbocyclic phosphinyl radicals with conjugated π-systems can be targeted realistically even without any further heteroatoms. It should be mentioned that well-developed synthetic methodologies are available in the literature for obtaining the precursors for our candidates. For example, phospholes, phosphinines, and phosphepins can be synthesized with various substituents in a straightforward manner, and these P-heterocycles seem to be appropriate starting materials to generate unprecedented stable radicals.

## 4 Computational Details

For the DFT and exact CCSD(T) computations, the Gaussian 09 suite of programs^69^ was used. For geometry optimizations, the ωB97X-D method was applied with the combination of the 6-311G** basis set. The unrestricted and restricted formalism was used for open-shell and closed-shell species, respectively. Harmonic vibrational analysis was obtained at the same level, and in the case of minima, all eigenvalues of the Hessian matrix were positive. Coupled-cluster (CCSD(T)/aug-cc-pVTZ) single-point energy calculations were carried out using the ωB97X-D/6-311G** optimized geometries. The spin populations were calculated using both Mulliken and natural population analyses, the latter using the NBO version 3.1.^70^ The energies are given in kcal·mol^–1^ in all cases. Optimized geometries for all investigated radicals and the corresponding phosphinyl analogues are provided in the Supporting Information.

The computation of accurate dimerization energies for the largest dimers of up to 162 atoms is highly challenging due to the large system size and the competition of large and opposite signed effects. Namely, for **12** and **21** with DTBP and BTMSP substituents in Table 8, the sum of mean-field repulsion and attractive electron correlation effects of about 80–90 kcal·mol^–1^ yield the final dimerization energies. Since such extensive dispersion contributions may pose a challenge for density functional approaches, we also estimated the corresponding CCSD(T) dimerization energies via the state-of-the-art local natural orbital (LNO) approach^71,72^ as implemented in the 2022 release of the MRCC quantum chemistry program suite.^73,74^ The development version of the MRCC was utilized for open-shell LNO-CCSD(T) computations.^75^ Extensive optimization of an efficient computational protocol on the example of **12** with DTBP substituents involved a systematically converging series of LNO-CCSD(T)/(aug)-cc-pVXZ (X = D, T, Q) results with loose, normal, and tight LNO approximation settings,^71^ as well as CBS extrapolation and counterpoise (CP) corrections. Our best converged result relying on CP correction, aug-cc-pV(T,Q)Z CBS extrapolation, and normal-tight extrapolation toward the local approximation free CCSD(T) limit has a remaining uncertainty estimate^71^ of about 1–2 kcal·mol^–1^ and differs by only 0.9 kcal·mol^–1^ from the protocol used in Table 8 utilizing LNO-CCSD(T)/cc-pV(D,T)Z CBS and normal-tight LNO extrapolation, and differs by only 0.9 kcal·mol^–1^ from the protocol used in Table 8 utilizing LNO/cc-pV(D,T)Z CBS and normal-tight LNO extrapolation.

## Supporting Information Available

Dimer conformations and energies of the dimers, and optimized geometries of the radicals and phosphine analogues including total energies (PDF)

## Dedication

This article is dedicated to Prof. Dietrich Gudat on the occasion of his 65th birthday.
