# Supplementary material for: Stability of Carbocyclic Phosphinyl Radicals: Effect of Ring Size, Delocalization, and Sterics
Source: Inorg Chem. 2022 Oct 4;61(41):16266–81. doi: 10.1021/acs.inorgchem.2c01968 (PMC9583709; doi:10.1021/acs.inorgchem.2c01968)
Supplement: Supplementary file 1 — ic2c01968_si_001.pdf [file ic2c01968_si_001.pdf]

Supporting Information

Stability of carbocyclic phosphinyl radicals: effect of ring size, delocalization, and sterics

Anna Ott [a], Péter R. Nagy [b,c], Zoltán Benkő[a,d]*

[a] Department of Inorganic and Analytical Chemistry, Faculty of Chemical Technology and Biotechnology, Budapest University of Technology and Economics, Műegyetem rkp. 3., H-1111 Budapest, Hungary, E-mail: benko.zoltan@vbk.bme.hu

[b] Department of Physical Chemistry and Materials Science, Faculty of Chemical Technology and Biotechnology, Budapest University of Technology and Economics, Műegyetem rkp. 3., H-1111 Budapest, Hungary

[c] ELKH-BME Quantum Chemistry Research Group, Műegyetem rkp. 3., H-1111 Budapest, Hungary

[d] ELKH-BME Computation Driven Chemistry Research Group, Műegyetem rkp. 3., H-1111 Budapest, Hungary

Table S1: Energies of smaller systems and the corresponding RSEs (kcal · mol^{-1}) at different levels of theory

Radical and phosphine analogue	ωB97X-D/6-311G**		CCSD(T)/aug-cc-pVTZ		W1U	
	Energy	RSE (kcal · mol^{-1})	Energy	RSE (kcal · mol^{-1})	Energy	RSE (kcal · mol^{-1})
**1**	-420. 539671	-4.2	-419.9570004	-3.1	-421.1929637	-3.0
**1H**	-419.9058792		-419.3255974		-420.5685293	
**3**	-459.85441	-3.7	-459.2020254	-2.6	-460.4891335	-2.6
**3H**	-459.21985		-458.5697134		-459.8639819	
**5**	-499.1934761	-4.1	-498.471919	-3.2	-499.8100188	-3.2
**5H**	-498.5595352		-497.8406757		-499.1859552	
**9**	-538.5031546	-2.8	-537.7113822	-1.9	-539.1009471	-1.9
**9H**	-537.8671297		-537.0780712		-538.4748065	
**2**	-419.2716825	-11.8	-418.6965538	-10.8	-419.9565168	-10.3
**2H**	-418.6500158		-418.0774298		-419.3436589	
**4**	-458.6127294	-8.5	-457.9689151	-6.9	-459.2794038	-6.6
**4H**	-457.9858333		-457.3435979		-458.660739	
**6**	-497.9540224	-11.0	-497.2411066	-8.9	-498.6024817	-9.0
**6H**	-497.3310652		-496.6189386		-497.987632	
**7**	-497.9581566	-3.7	-497.2450089	-2.8	-498.6061942	-2.9
**7H**	-497.3235374		-496.6130238		-497.9815189	
**8**	-496.7274806	-9.3	-496.0227936	-7.6	-497.407612	-7.5
**8H**	-496.1017646		-495.3985074		-496.7902844	

Figure S1: Anti (D1) and gauche-like (D2) conformations of the dimers; sym and asym denote the symmetrically and asymmetrically substituted dimers

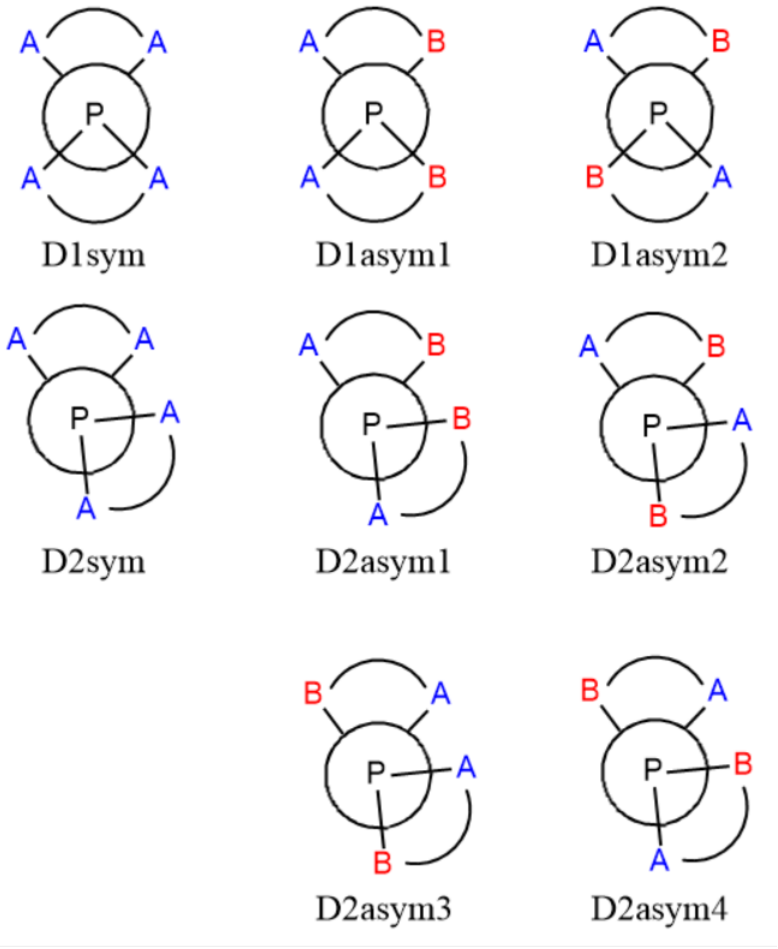

Table S2.: Different conformations and formation energies of the dimers with their smallest C – P – P – C dihedral angles

	Anti				Gauche					
Dimer	Dimerisation energy (kcal · mol^{-1})	Dihedral angle (°)	Dimerisation energy (kcal · mol^{-1})	Dihedral angle (°)	Dimerisation energy (kcal · mol^{-1})	Dihedral angle (°)	Dimerisation energy (kcal · mol^{-1})	Dihedral angle (°)	Dimerisation energy (kcal · mol^{-1})	Dihedral angle (°)
**{1}$_2$**	-51.8	132.2	-	-	-54.3	265.1	-	-	-	-
**{3}$_2$**	-55.2	126.7	-	-	-54.5	149.9	-	-	-	-
**{5}$_2$**	-53.4	99.4	-	-	-55.0	154.9	-	-	-	-
**{9}$_2$**	-56.1	81.6	-	-	-56.5	176.7	-	-	-	-
**{14}$_2$**	-51.2	86.2	-	-	-54.7	177.1	-	-	-	-
**{4}$_2$**	-40.3	138.0	-46.8	132.2	-39.3	135.3	-48.1	138.7	-49.0	138.5
**{6}$_2$**	-47.5	114.9	-44.5	89.9	-48.9	152.3	-46.2	152.6	-46.5	167.6
**{7}$_2$**	-45.3	87.2	-	-	-46.8	170.1	-	-	-	-
**{10}$_2$**	-55.0	110.7	-46.2	97.5	-56.4	171.0	-46.2	175.1	-47.6	173.7
**{11}$_2$**	-48.2	116.4	-56.8	86.6	-48.0	174.4	-57.6	154.1	-57.3	166.3
**{13}$_2$**	-41.7	103.5	-38.7	95.3	-46.0	163.1	-37.7	178.4	-41.2	172.3
**{15}$_2$**	-53.6	83.6	-47.8	82.1	-54.1	167.8	-46.1	151.0	-49.5	174.4
**{16}$_2$**	-47.4	90.0	-57.6	88.2	-47.1	179.8	-57.6	179.7	-53.8	168.7
**{17}$_2$**	-57.7	91.3	-	-	-55.8	172.7	-	-	-	-
**{19}$_2$**	-46.5	173.8	-44.5	97.0	-28.0	174.3	-50.2	160.0	-50.0	177.4
**{20}$_2$**	-44.0	175.8	-44.5	83.3	-43.2	152.0	-43.9	177.1	-45.9	174.0
**{2}$_2$**	-58.0	138.1	-	-	-56.9	281.2	-	-	-	-
**{8}$_2$**	-48.4	89.2	-	-	-46.8	159.3	-	-	-	-
**{12}$_2$**	-40.0	90.5	-	-	-36.3	166.4	-	-	-	-
**{18}$_2$**	-44.0	176.1	-	-	-44.0	176.1	-	-	-	-
**{21}$_2$**	-39.0	132.8	-	-	-41.4	167.6	-	-	-	-

*Table S3: P – C and C – C bond distances in species **2**, **8**, **12** and **21** in A at the ωB97X-D/6311-G** level of theory. The numbering of the C atoms is continuous starting from the P center. The s-character of the lone pair at the P-center is given in %.*

	**2**	**8**	**12**	**21**
P-C1	1.819	1.788	1.786	1.780
C1-C2	1.303	1.354	1.345	1.357
C2-C3		1.466	1.495	1.448
C3-C4				1.350
s% in lone pair	83.8%	66.3%	65.0%	63.8%

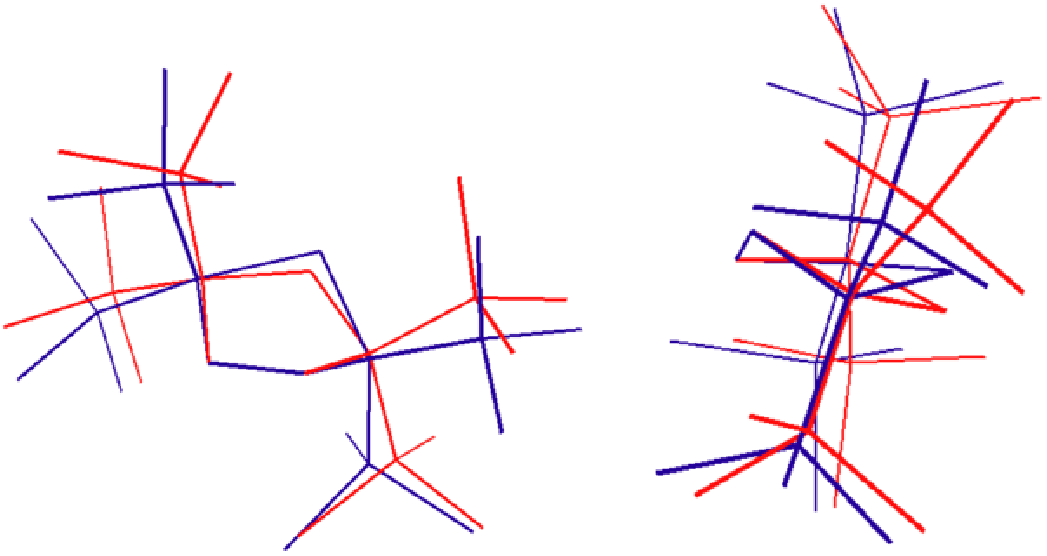

Figure S2: Two different views of the overlapping structures of radical ***IV****: red: equilibrium geometry, blue: adapted structure in the dimer at the ωB97X-D/6311-G** level of theory.*

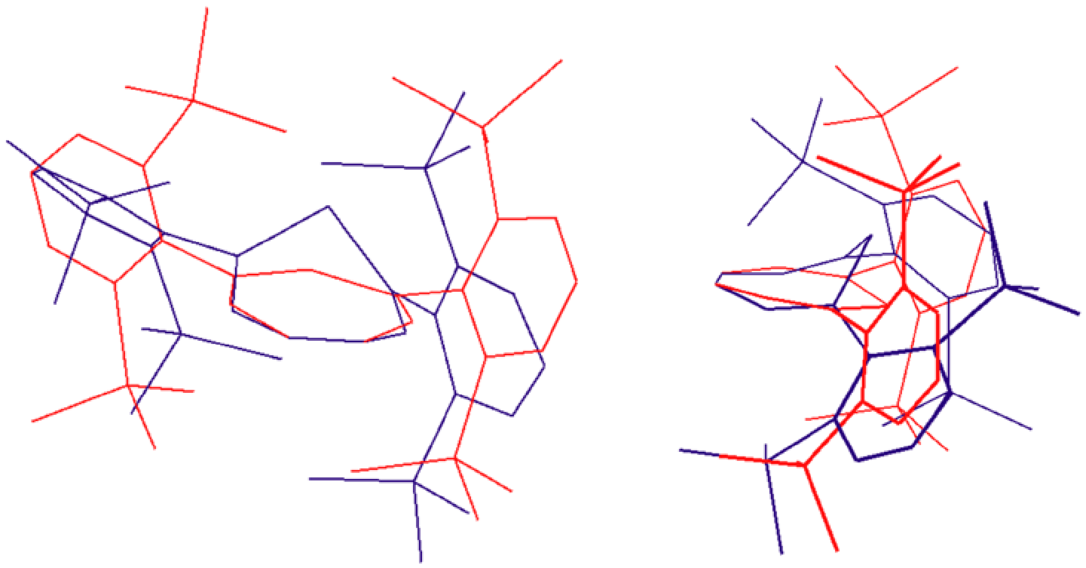

*Figure S3: Two different views of the overlapping structures of radicals 21DTBP: red: equilibrium geometry, blue: adapted structure in the dimer at the ωB97X-D/6311-G** level of theory.*

Total energies and optimized geometries in Cartesian coordinates

1
E = -419.905879243

P -0.91456500 -0.00000900 -0.00000400

C 0.78120400 -0.74646600 -0.00001600

C 0.78116500 0.74646800 0.00001900

H 1.08589500 1.25706200 -0.90840900

H 1.08620000 1.25735600 0.90815700

H 1.08587300 -1.25700600 0.90845700

H 1.08629200 -1.25728900 -0.90816900

1H
E = -420.539671032

P 0.88292100 -0.00006400 -0.10335400

C -0.82279100 -0.74498800 0.01344700

C -0.82300600 0.74491700 0.01303800

H -1.08332500 1.25488200 0.93239600

H -1.16505300 1.24895000 -0.88396800

H -1.16333400 -1.24798500 -0.88474100

H -1.08528100 -1.25569200 0.93177100

H 1.12795900 0.00122900 1.29594700

2
E = -418.650015765

C -0.83403200 0.65158000 0.00003300

C -0.83403200 -0.65158000 0.00003200

P 0.86399700 -0.00000000 -0.00000700

H -1.47578600 -1.51882100 -0.00014100

H -1.47578600 1.51882100 -0.00014300

2H
E = -419.271682541

P 0.83241200 -0.00000400 -0.10233200

C -0.88866300 -0.64540500 0.01515600

C -0.88861800 0.64544200 0.01509800

H -1.50694700 1.52826100 0.03222100

H -1.50690200 -1.52838400 0.03204400

H 1.19135200 -0.00004000 1.28919700

3
E = -459.219850036

P 1.13889800 0.00000100 0.07423300

C -0.32549700 1.15204700 -0.13695600

C -1.30652400 -0.00000500 0.17218600

C -0.32549300 -1.15204400 -0.13695600

H -1.57074800 -0.00000800 1.23219400

H -2.23533000 -0.00000300 -0.40500000

H -0.40299600 -1.48902900 -1.17579700

H -0.36314500 -2.02975900 0.51063400

H -0.36314200 2.02976400 0.51063400

H -0.40303100 1.48902200 -1.1757700

3H
E = -459.854409956

P 1.11862100 0.00003500 -0.01264700

C -0.36782700 1.15026800 -0.16321900

C -1.32641200 -0.00007000 0.20688600

C -0.36772300 -1.15024900 -0.16319800

H -1.51033700 -0.00002600 1.28483100

H -2.29475900 -0.00017100 -0.30287600

H -0.47757900 -1.45341100 -1.20722600

H -0.38704700 -2.04108000 0.46423800

H -0.38747500 2.04097200 0.46435000

H -0.47761900 1.45342000 -1.20725300

H 1.12726600 0.00007500 1.41082200

4
E = -457.985833275

P 1.08279100 -0.01825600 0.00000600

C -0.29920800 1.12099800 -0.00001300

C -1.27307500 0.18243600 -0.00000100

C -0.47926700 -1.10081000 -0.00000800

H -0.60157800 -1.72241800 -0.89029800

H -2.35404400 0.30118000 0.00005700

H -0.37533200 2.20174600 -0.00000500

H -0.60161100 -1.72241300 0.89028400

4H
E = -458.612729372

P -1.04912600 -0.03196400 0.11397300

C 0.42724400 -1.11358700 -0.03746200

C 1.31523000 -0.11701300 -0.00091100

C 0.45193800 1.11986600 -0.01986600

H -1.47767100 0.00334700 -1.24489900

H 0.49556300 1.68272900 -0.95468300

H 2.40035700 -0.16116600 0.05331900

H 0.57213200 -2.18663600 -0.03432800

H 0.58003500 1.80559100 0.82042700

5
E = -498.559535162

C -0.07240800 -1.33445800 0.12955200

C 1.27366700 -0.71613600 -0.26814500

C 1.27375600 0.71601300 0.26820800

C -0.07214000 1.33441200 -0.12963800

P -1.37325200 0.00010400 0.00002600

H -0.04356300 -1.67749000 1.17120500

H -0.34356600 -2.20085800 -0.47891700

H 2.11686400 -1.29760200 0.11455200

H 1.36048500 -0.69485700 -1.36020600

H 1.36074900 0.69462500 1.36027200

H 2.11709000 1.29728500 -0.11448700

H -0.34348300 2.20090500 0.47863800

H -0.04304400 1.67745000 -1.17131700

5H
E = -499.193476100

P 1.34060600 0.02573000 -0.03828100

C 0.06054900 -1.32116300 -0.24138900

C -1.24951000 -0.75990700 0.31625400

C -1.34882500 0.68045400 -0.19005100

C 0.01429200 1.36374500 0.03440400

H -0.04113100 -1.53975600 -1.30868800

H 0.38462000 -2.23939400 0.25215900

H -2.11476400 -1.35636200 0.01151000

H -1.21390800 -0.76702200 1.41159900

H -1.57334600 0.66262500 -1.26212900

H -2.15789200 1.23045300 0.29759900

H 0.20974400 2.12765100 -0.71949400

H 0.05655800 1.85157900 1.01013500

H 1.48199600 -0.13450500 1.36621600

6
E = -497.331065230

P -1.33316300 0.06603100 -0.03801500

C -0.18363600 -1.28655400 0.03303300

C 1.12083300 -0.93783600 0.02818800

C 1.41358500 0.52955800 -0.06847800

H 1.87815800 0.74112800 -1.03984500

H 1.92846400 -1.66326400 0.05258900

H -0.50780900 -2.32140000 0.05970500

H 2.14299000 0.83867100 0.68746300

C 0.07923600 1.28839600 0.07818800

H -0.03750700 2.07521600 -0.67042300

H 0.01305100 1.77780400 1.05515500

6H

E = -497.954022440

C -0.07243300 1.31636500 0.07196400

C 1.19016500 0.89977700 0.04701100

C 1.41634700 -0.58161200 -0.10329400

C 0.06827300 -1.29366700 0.14759100

P -1.28969700 -0.02952900 -0.16440300

H -1.87233200 -0.01526300 1.13245400

H 2.19162600 -0.94065000 0.58064700

H 2.03571300 1.58055300 0.08535000

H -0.35554700 2.36151800 0.12259900

H 1.78105400 -0.79024600 -1.11633600

H 0.00817800 -1.63641800 1.18150000

H -0.05734100 -2.16173700 -0.49979700

7

E = -497.323537384

C 0.01388100 1.34396500 0.00008900

C -1.32714500 0.66369900 -0.00010400

C -1.32729800 -0.66353100 -0.00008700

C 0.01365600 -1.34397400 0.00008700

P 1.31157600 -0.00006800 -0.00001800

H -2.24009000 1.25092100 0.00011300

H 0.14234500 1.99192000 -0.87558000

H -2.24028400 -1.25064100 0.00005100

H 0.14221900 -1.99198500 -0.87550800

H 0.14169700 -1.99221700 0.87559800

H 0.14191100 1.99207200 0.87568800

7H

E = -497.958156641

C -0.01985200 1.34615900 -0.13572900

C -1.34602000 0.66389100 0.07537700

C -1.34583500 -0.66411400 0.07551300

C -0.01956600 -1.34607800 -0.13579500

P 1.27909600 0.00010700 -0.00026400

H -2.25416100 1.24895600 0.18178700

H 0.02607700 1.81496000 -1.12449800

H -2.25377600 -1.24945600 0.18218300

H 0.02639200 -1.81475400 -1.12468200

H 0.17077300 -2.13482200 0.59650000

H 0.17046300 2.13446800 0.59698700

H 1.31542700 -0.00011700 1.41948900

8

E = -496.101764598

C 0.00056700 1.26936000 -0.00001800

C -1.24389300 0.73473300 -0.00014500

C -1.24669300 -0.73105800 0.00008500

C -0.00460600 -1.26995400 0.00011100

P 1.25828200 -0.00167500 -0.00002100

H -2.15998500 1.31493900 0.00024300

H 0.21309200 2.32996000 0.00034500

H -2.16527300 -1.30725100 -0.00020900

H 0.20568500 -2.33100400 -0.00025800

8H

E = -496.727480583

C -0.04906300 1.28060900 0.04280100

C -1.27744700 0.72891600 0.00257300

C -1.27740800 -0.72854500 0.00272400

C -0.04933200 -1.28065800 0.04305800

P 1.21713700 -0.00019000 -0.11791800

H -2.19672400 1.30353900 -0.02547500

H 0.15376100 2.34315300 0.03821300

H -2.19669000 -1.30324900 -0.02577700

H 0.15226600 -2.34345100 0.03786600

H 1.74983100 0.00092900 1.1970020

9
E = -537.874087132

C -0.44404000 1.38934000 0.31342400

P -1.56350800 0.00051500 -0.20034600

C -0.44512300 -1.38908300 0.31353100

C 0.97641300 -1.27725200 -0.25896900

C 1.69692400 -0.00072700 0.17877000

C 0.97764900 1.27667700 -0.25875100

H 1.79765500 -0.00048100 1.27192800

H 2.71256800 -0.00088500 -0.22836800

H 1.55688200 -2.15159000 0.05370600

H 0.93075000 -1.30904400 -1.35416600

H -0.90956300 -2.32958900 0.00650100

H -0.39509300 -1.40207400 1.41072900

H 1.55868900 2.15042400 0.05454800

H 0.93207600 1.30916100 -1.35389100

H -0.90784400 2.32988400 0.00560500

H -0.39444800 1.40274800 1.41056700

9H
E = -538.508403408

C -0.41118000 1.39189300 0.34226000

P -1.47657100 0.00014200 -0.29159500

C -0.41146200 -1.39213200 0.34211700

C 0.99990200 -1.28198000 -0.24794600

C 1.72459600 -0.00007500 0.17575400

C 1.00009000 1.28176500 -0.24797000

H 1.84137400 0.00011200 1.26726400

H 2.73422900 -0.00005500 -0.24577100

H 1.58906300 -2.15061700 0.06271500

H 0.94207400 -1.31989500 -1.34279100

H -0.87139000 -2.34115000 0.05553600

H -0.35660400 -1.36603200 1.43538100

H 1.58913000 2.15054300 0.06280000

H 0.94207400 1.32028300 -1.34277500

H -0.87095900 2.34078000 0.05499800

H -0.35617500 1.36651500 1.43551900

H -2.44593300 0.00055800 0.74575300

10
E = -536.645182251

P 1.57116100 -0.00474100 -0.03239800

C 0.46365700 1.37970100 -0.07723600

C -0.88406600 1.35560900 0.04920800

C -1.71381500 0.11169700 0.17029700

C -1.02581100 -1.13752100 -0.37821800

C 0.34313500 -1.37137500 0.26276700

H -0.90723900 -1.03320600 -1.46211400

H -2.66887100 0.26701300 -0.34154200

H 0.94335300 2.34852300 -0.19625900

H -1.66633800 -2.00859900 -0.20964300

H 0.79170100 -2.29784700 -0.10563800

H 0.23107200 -1.49192700 1.34805200

H -1.42201600 2.30096300 0.06278400

H -1.96767700 -0.04247500 1.22941500

10H
E = -537.272130654

P 1.54495700 0.04468200 0.03983300

C 0.35492500 1.43078500 -0.06450700

C -0.96832300 1.32789500 0.05561200

C -1.75230800 0.04582200 0.13375000

C -0.99472700 -1.17787100 -0.37928800

C 0.35545500 -1.35489500 0.31514500

H 1.73967900 -0.14675600 -1.35650700

H -0.83661300 -1.07583800 -1.45843700

H -2.67907000 0.16805100 -0.43633400

H 0.79815500 2.42078300 -0.13172800

H -1.60905000 -2.07131200 -0.22947000

H 0.85174900 -2.27198800 -0.01332300

H 0.21217800 -1.44820400 1.39684800

H -1.55515400 2.24397200 0.09365000

11
E = -536.633731131

C 0.37908400 1.36044200 0.37987400

C -0.98078200 1.26397800 -0.25003000

C -1.67552300 0.12973800 -0.21472500

C -1.08500900 -1.10368800 0.40981400

C 0.30266300 -1.41528400 -0.18505700

P 1.53529800 -0.02287200 -0.12691200

H -2.65940300 0.07252300 -0.66825900

H -1.38343200 2.14469600 -0.74041600

H 0.86754700 2.31024400 0.15607800

H -1.74520100 -1.96331500 0.27194800

H -0.97936700 -0.96163100 1.49315500

H 0.75181700 -2.27905000 0.31598500

H 0.18673300 -1.69764300 -1.23812000

H 0.28922800 1.30613500 1.47405200

11H
E = -537.272362593

C 0.54791400 1.40230100 -0.22208200

C -0.94485000 1.35141800 -0.04115500

C -1.68530900 0.25933400 0.12635400

C -1.19308000 -1.16318500 0.14400300

C 0.20453200 -1.36025300 -0.44198100

P 1.47674500 -0.15766300 0.18048300

H -2.75509900 0.38728800 0.27007700

H -1.44946300 2.31519000 -0.04929600

H 0.79310800 1.67593800 -1.25532100

H -1.90730500 -1.78629300 -0.40673900

H -1.21548600 -1.53202800 1.17761500

H 0.56312100 -2.37475200 -0.25141900

H 0.17746900 -1.23745200 -1.53009200

H 0.96926400 2.20081300 0.39712000

H 1.09797700 -0.22145400 1.54997800

12
E = -535.415385812

P 1.55206700 -0.00008500 -0.00000600

C 0.39489600 1.35966400 0.00002600

C -0.94758300 1.27028500 -0.00003600

C -1.73663300 0.00020300 -0.00003100

C -0.94789100 -1.27008400 -0.00004400

C 0.39450500 -1.35995700 0.00006000

H -2.41734200 0.00027700 -0.86565700

H 0.84037300 2.35114100 -0.00012100

H -1.53553900 -2.18547000 0.00014200

H 0.83983200 -2.35148100 -0.00011300

H -1.53491600 2.18585600 0.00024500

12H
E = -536.033610747

P 1.49846100 -0.00006700 0.18110600

C 0.34372600 1.38064200 -0.10488700

C -0.98114300 1.27221900 -0.06712800

C -1.75961100 0.00007200 0.12225300

C -0.98125600 -1.27220300 -0.06701500

C 0.34360300 -1.38066700 -0.10495700

H 2.13083900 0.00026000 -1.09324500

H -2.60988900 -0.00003500 -0.57081500

H 0.79392100 2.35981700 -0.24086800

H -1.58058300 -2.17262700 -0.18128500

H 0.79378100 -2.35984200 -0.24104000

H -1.58038600 2.17272300 -0.18133100

H -2.21650500 0.00032400 1.12239700

13
E = -535.415572468

P -1.51044800 0.01999300 0.00028200

C -0.32487800 -1.42150600 -0.00047800

C 1.14666400 -1.14529600 0.00027900

C 1.71086800 0.07547700 0.00019000

C 0.96734700 1.30673600 -0.00027900

C -0.40621300 1.36616800 -0.00011100

H 2.79360900 0.14754500 0.00055800

H -0.57658300 -2.04969600 -0.86692100

H 1.53163900 2.23412900 -0.00053700

H -0.87138700 2.34867700 -0.00010100

H 1.79404200 -2.01856700 0.00073100

H -0.57732500 -2.05145500 0.86442900

13H
E = -536.034422552

P 1.39226000 -0.03154000 -0.31435800

C 0.35143400 1.34287500 0.39252500

C -1.07191200 1.20035300 -0.09476900

C -1.69339500 0.02121200 -0.17820000

C -1.04161700 -1.25614000 0.13847600

C 0.28658900 -1.40701200 0.16031300

H 2.30554400 -0.15794500 0.76392600

H -2.73490900 -0.01125900 -0.48080300

H 0.77178500 2.30027100 0.08100700

H -1.68446000 -2.10994100 0.33260300

H 0.72700400 -2.38029700 0.35169000

H -1.61614100 2.10904300 -0.33165000

H 0.36067800 1.31549400 1.48851700

14
E = -577.178108782

C 0.79336900 1.18598600 0.70418000

C -0.52597700 1.72363200 0.10443800

C -1.33696700 0.72194800 -0.72092400

C -1.81261200 -0.53821900 0.00706800

C -0.71438500 -1.40857600 0.64067100

C 0.55654300 -1.54055700 -0.20257800

P 1.70343300 -0.06350400 -0.32504100

H -2.36595900 -1.14676400 -0.71709300

H -2.21572200 1.23727900 -1.12222100

H -1.12683600 -2.40828000 0.81280400

H -1.14556900 2.11900800 0.91733800

H 0.61336200 0.74599100 1.69011900

H -0.74427700 0.42866500 -1.59641900

H -2.53406200 -0.26138200 0.78400200

H -0.45738800 -1.02664300 1.63260900

H 1.17062800 -2.36257600 0.18272300

H 0.30040000 -1.82836200 -1.23044800

H -0.28991300 2.57307700 -0.54383700

H 1.48401100 2.01726700 0.86890400

14H

E = -577.813092384

C 0.64531500 1.22781600 0.71703200

C -0.63844300 1.71721300 0.03410800

C -1.43344200 0.63032800 -0.69603300

C -1.79855900 -0.61962300 0.11420500

C -0.62375800 -1.49009000 0.61217800

C 0.59247600 -1.49756500 -0.31813300

P 1.65526200 0.05686700 -0.32882200

H -2.43643900 -1.23958900 -0.52511100

H 2.53190000 -0.32411600 0.72119200

H -2.36038000 1.07624200 -1.07101100

H -0.98612000 -2.51705800 0.72614200

H -1.27515700 2.19131200 0.78965600

H 0.40969800 0.76293400 1.67641500

H -0.87769600 0.32758200 -1.59190200

H -2.41834600 -0.33216100 0.97101800

H -0.31415700 -1.17950900 1.61368800

H 1.25792000 -2.32442400 -0.05667000

H 0.27073200 -1.67924800 -1.34788500

H -0.38709300 2.49666800 -0.69240300

H 1.29466500 2.07989100 0.939061000

15

E = -575.948883233

P 1.78254200 0.13536600 0.11570300

C 0.88119300 -1.32993600 -0.34038400

C -0.41961500 -1.65662900 -0.16188300

C -1.42266000 -0.83730500 0.59888000

C -1.88070700 0.44979400 -0.10759600

C -0.73730900 1.33754800 -0.61214300

H -2.49466400 1.01391700 0.60323300

H -2.30196300 -1.44802000 0.81833400

H -1.13459200 2.34099200 -0.79438600

H -0.76193200 -2.60924700 -0.56051100

H 1.51161000 -2.07321200 -0.82654500

H -0.98819800 -0.57187600 1.56899200

C 0.45515100 1.42715200 0.35287100

H -2.53301500 0.19657000 -0.94931700

H -0.38735700 0.96826400 -1.58087900

H 0.98088500 2.37623000 0.21165100

H 0.11476800 1.43215600 1.39541400

15H

E = -576.578788709

P -1.70569500 0.04271400 -0.27066000

C -0.76495700 -1.35881600 0.43537000

C 0.51400700 -1.64574600 0.19911900

C 1.41845800 -0.79269500 -0.65117800

C 1.87608100 0.49797700 0.05024300

C 0.73545700 1.32546000 0.65981600

H 2.41548400 1.10763200 -0.68354900

H -2.35083300 0.37756200 0.94837800

H 2.29981900 -1.36444600 -0.95262600

H 1.12270200 2.32054900 0.90174600

H 0.94535800 -2.53134800 0.65982200

H -1.35247100 -2.02590600 1.06164000

H 0.89812400 -0.53223900 -1.58066800

C -0.47728600 1.45994900 -0.26711700

H 2.59510300 0.25297700 0.83886400

H 0.42154900 0.87811000 1.60732100

H -1.06913900 2.33999700 0.00054200

H -0.15082300 1.61963700 -1.29909200

16
E = -575.942600440

P -1.65605200 -0.03359600 -0.40240900

C -0.73655100 -1.25498300 0.65739100

C 0.57723500 -1.66394400 0.03010000

C 1.58736000 -0.88146000 -0.34598500

C 1.74581100 0.61137500 -0.23710300

C 0.71298100 1.38402000 0.58604500

H 1.78327800 1.03081800 -1.25250300

H 2.43964200 -1.37853700 -0.80486000

H 1.10136600 2.39564200 0.74652200

H 0.68476800 -2.72823700 -0.16220400

H -1.37715100 -2.13340200 0.75726300

C -0.66724300 1.51203100 -0.06136300

H 2.73930300 0.80261000 0.18583200

H 0.62876600 0.93719400 1.58103900

H -1.30119600 2.15320200 0.56427700

H -0.57689300 2.03691900 -1.02013300

H -0.59865700 -0.85450500 1.66638600

16H
E = -576.577962106

P -1.71547600 0.06282300 -0.25618300

C -0.71707800 -1.24189300 0.63745500

C 0.49273900 -1.64927300 -0.15990300

C 1.58376600 -0.91689000 -0.37560200

C 1.90229500 0.48651900 0.07412000

C 0.74762400 1.38624500 0.53858000

H 2.39360400 0.98493800 -0.77128100

H 2.37434600 -1.38010800 -0.96228100

H 1.14879500 2.39996600 0.63990900

H 0.46205300 -2.64021300 -0.60433900

H -1.37383700 -2.10150000 0.78540600

C -0.43187100 1.40880100 -0.43962700

H 2.66691800 0.43997900 0.86032800

H 0.41006100 1.10195900 1.53922000

H -0.98191800 2.35104600 -0.36087100

H -0.05728800 1.36110100 -1.46644400

H -2.34802900 0.58879300 0.90384300

H -0.42741000 -0.88937100 1.62911900

17
E = -575.941813006

C -0.59506600 -1.31323800 0.53985000

C 0.61039100 -1.56854900 -0.38012200

C 1.78383100 -0.64269100 -0.17317700

C 1.78385100 0.64260500 0.17332400

C 0.61046600 1.56859800 0.37995900

C -0.59497900 1.31315900 -0.53997700

P -1.78621000 0.00006200 0.00009100

H 2.75654100 -1.10899100 -0.31133100

H 0.96428000 2.58963500 0.20531700

H 0.29689800 -1.54868400 -1.43222400

H -1.18366700 -2.22976700 0.64160400

H 2.75656800 1.10879800 0.31178800

H 0.29693700 1.54903800 1.43205800

H -1.18351300 2.22970800 -0.64192400

H -0.23294700 1.07024200 -1.54676800

H -0.23302600 -1.07055300 1.54669700

H 0.96410600 -2.58966100 -0.20572500

17H

E = -576.577232598

P -1.72603000 -0.09143900 -0.13839100

C -0.51679300 -1.30539100 0.59213600

C 0.68426900 -1.58147600 -0.32211300

C 1.82165500 -0.59656100 -0.20551000

C 1.79075400 0.69109500 0.13320800

C 0.59395200 1.56890200 0.41978300

H 2.80173900 -1.02040200 -0.41271800

H 0.91831500 2.60935600 0.31884100

H 0.36021500 -1.65404300 -1.36981400

H -1.07089500 -2.23368700 0.75524300

C -0.60158700 1.34062900 -0.51523300

H 2.75113700 1.19757300 0.19203000

H 0.28205600 1.46025600 1.46613100

H -1.24691300 2.22474300 -0.52782900

H -0.23993500 1.21385400 -1.53976800

H -0.15680400 -0.96911400 1.56760100

H -2.22789600 0.41051200 1.09319000

H 1.08593900 -2.57064000 -0.08067000

18

E = -574.717819545

P 1.75249800 -0.00035700 -0.00016900

C 0.70726500 -1.43214100 -0.17895500

C -0.60810200 -1.65169000 0.02295100

C -1.65361600 -0.64887700 0.41195000

C -1.65328200 0.64993400 -0.41213800

C -0.60680500 1.65195100 -0.02290100

H -2.63544100 -1.12001600 0.31704500

H -0.95676200 2.67615400 0.09034900

H -0.95871000 -2.67574300 -0.08973300

H 1.30217500 -2.30948100 -0.43009700

C 0.70835000 1.43130700 0.17938400

H -1.53519200 0.39799000 -1.47356200

H 1.30360900 2.30842000 0.43055400

H -1.53537300 -0.39678200 1.47332800

H -2.63463500 1.12190400 -0.31709600

18H

E = -574.717819545

P 1.75249800 -0.00035700 -0.00016900

C 0.70726500 -1.43214100 -0.17895500

C -0.60810200 -1.65169000 0.02295100

C -1.65361600 -0.64887700 0.41195000

C -1.65328200 0.64993400 -0.41213800

C -0.60680500 1.65195100 -0.02290100

H -2.63544100 -1.12001600 0.31704500

H -0.95676200 2.67615400 0.09034900

H -0.95871000 -2.67574300 -0.08973300

H 1.30217500 -2.30948100 -0.43009700

C 0.70835000 1.43130700 0.17938400

H -1.53519200 0.39799000 -1.47356200

H 1.30360900 2.30842000 0.43055400

H -1.53537300 -0.39678200 1.47332800

H -2.63463500 1.12190400 -0.31709600

19

E = -574.713080139

P -1.72218900 0.07060800 -0.10037100

C -0.63049300 -1.29945400 0.54194400

C 0.55500700 -1.56715000 -0.34091800

C 1.60818100 -0.75904200 -0.39752300

C 1.70949000 0.50979500 0.41579400

C 0.72293700 1.58059100 0.01778500

H 2.42843600 -0.99202200 -1.06872900

H 1.14322900 2.57684700 -0.10034400

H 0.51849100 -2.44694600 -0.97620500

H -1.26019300 -2.18622800 0.63455700

C -0.60631900 1.44673500 -0.20946800

H 1.56480600 0.27547300 1.48097700

H -1.12314300 2.35284800 -0.52281100

H 2.71800700 0.91952600 0.33739900

H -0.30961900 -1.02746300 1.55503100

19H

E = -575.340050543

P -1.63789500 0.04246500 -0.28864100

C -0.65121300 -1.22973900 0.64982500

C 0.52584100 -1.62033700 -0.19512800

C 1.58780800 -0.84448800 -0.38897800

C 1.81776800 0.50411700 0.25005200

C 0.77124400 1.58262100 0.07780900

H 2.37836100 -1.20788500 -1.03859800

H 1.18337200 2.58813800 0.14605500

H 0.47230800 -2.57323400 -0.71322400

H -1.29571800 -2.08757400 0.85003000

C -0.54608200 1.49597500 -0.10614400

H 1.98184200 0.36461200 1.32942600

H -1.08611500 2.43553300 -0.20834000

H 2.76175300 0.90755200 -0.12527300

H -2.52741500 0.41820400 0.75617700

H -0.33215800 -0.81120700 1.60873900

20

E = -574.721000053

P -1.74630100 0.00881700 -0.00816700

C -0.59182800 -1.37792600 0.41025500

C 0.67916600 -1.48315500 -0.45130100

C 1.79310200 -0.58978100 0.00385000

C 1.73532600 0.74225100 0.19847000

C 0.63230800 1.64682300 -0.00095100

H 2.75259000 -1.06883500 0.17795300

H 0.92522400 2.69426600 -0.03412800

H 0.43448100 -1.25456400 -1.49679000

H -1.18449900 -2.29214200 0.31081400

C -0.71552200 1.40802900 -0.15874200

H 2.66304300 1.22728400 0.49090400

H -1.30635600 2.30073100 -0.36235400

H -0.31856500 -1.29887300 1.46841300

H 1.03327900 -2.51757400 -0.44179600

20H

E = -575.346814509

P -1.51731800 -0.20045800 -0.43270900

C -0.46149900 -1.33043200 0.60625100

C 0.91349400 -1.56004600 -0.01058700

C 1.80277200 -0.36950900 -0.22533000

C 1.59699300 0.94839500 -0.09187500

C 0.40566600 1.73589600 0.23860600

H 2.81209400 -0.65200700 -0.51889900

H 0.62707000 2.75458800 0.54969500

H 0.80725500 -2.07784700 -0.97183200

H -0.98058200 -2.28925500 0.68933300

C -0.88695200 1.39947700 0.14679500

H 2.47350100 1.56796800 -0.26835200

H -1.63107500 2.15112700 0.39529200

H -0.36494300 -0.91194200 1.61037300

H -2.67180400 -0.21854500 0.39152500

H 1.46541400 -2.25989100 0.63033800

21

E = -573.492246822

C -0.67033900 -1.42105000 0.00022200

C 0.67077100 -1.62526000 -0.00027500

C 1.76332900 -0.67559400 -0.00002300

C 1.76390700 0.67456900 0.00018600

C 0.67184200 1.62501900 0.00017400

C -0.66941900 1.42156600 -0.00028500

P -1.74233500 0.00037200 0.00006700

H 2.74566200 -1.14021400 -0.00031800

H 0.99435600 -2.66350700 -0.00052500

H -1.26475500 -2.33492900 0.00040300

H -1.26323600 2.33582200 -0.00089400

H 0.99599400 2.66309700 -0.00001600

H 2.74645900 1.13865100 0.00035100

21H

E = -574.114770045

C -0.65173800 -1.37421000 0.38889100

C 0.67226400 -1.56240300 0.33060800

C 1.64854500 -0.67386700 -0.28745100

C 1.64816000 0.67478100 -0.28779500

C 0.67101800 1.56274300 0.33045100

C -0.65288100 1.37405600 0.38905900

P -1.46842000 -0.00044600 -0.49821800

H 2.52596100 -1.16300300 -0.70315900

H 1.08533700 -2.45979700 0.78670900

H -1.26879300 -2.08391300 0.93234200

H -1.26990100 2.08320400 0.93323700

H 1.08395300 2.46023400 0.78656600

H 2.52518200 1.16454700 -0.70349900

H -2.66764500 -0.00118200 0.25850500

22

E = -496.043466510

C 0.71423700 0.26305000 0.00000700

C -0.71426300 0.26300800 -0.00002300

P 0.00004000 -1.38256700 0.00000400

C -1.81170400 1.01910800 -0.00003100

C 1.81165100 1.01918100 0.00003500

H -2.79977900 0.57382100 0.00013800

H -1.74278100 2.10230500 0.00000900

H 2.79973700 0.57391900 -0.00019300

H 1.74270300 2.10237700 0.00005000

22H

E = -496.665157261

C -0.70855100 0.32524100 -0.00153200

C 0.70852100 0.32530900 -0.00140800

P -0.00002400 -1.37475200 -0.09434200

H -0.00020300 -1.61817300 1.30900600

C 1.80391400 1.06661000 0.00656700

C -1.80383400 1.06672100 0.00642000

H 2.79002500 0.61718800 0.00083500

H 1.74143400 2.15070600 0.02166300

H -2.79002800 0.61747000 0.00125300

H -1.74116800 2.15079600 0.02208400

23

E = -535.382656326

C -1.10982200 -0.18351500 -0.00003100

C -0.00018300 -1.22571800 -0.00026800

C 1.10966300 -0.18380600 -0.00023000

P 0.00028200 1.24237900 -0.00004900

H -0.00025900 -1.86628500 0.88784300

H -0.00030000 -1.86521800 -0.88914600

C 2.44024500 -0.31978200 0.00032500

C -2.44043400 -0.31945100 0.00028700

H 3.09670900 0.54398900 0.00038200

H 2.90921600 -1.29950100 0.00049800

H -3.09701700 0.54418500 -0.00018800

H -2.90938100 -1.29921600 0.00085600

23H

E = -536.001282495

C -1.12471100 -0.22585000 0.01477200

C -0.00014400 -1.23610300 0.18984500

C 1.12464300 -0.22605900 0.01466900

P 0.00007000 1.25140100 -0.03170500

H 0.00008600 -1.66257800 1.19995200

H -0.00046300 -2.06014900 -0.53100100

H 0.00041400 1.49822700 1.37097000

C 2.42576000 -0.38361700 -0.16137600

C -2.42573100 -0.38350000 -0.16152700

H 3.08682800 0.46306400 -0.31251400

H 2.87939200 -1.37102100 -0.15917000

H -3.08690700 0.46318600 -0.31197000

H -2.87930000 -1.37095600 -0.15899400

24

E = -574.722373004

C -1.28697500 -0.00873600 0.04805300

C -0.71520200 1.37786000 0.27503900

C 0.71514800 1.37796600 -0.27476600

C 1.28699600 -0.00865000 -0.04821300

P -0.00000700 -1.26527700 0.00017100

H -1.33851900 2.15080200 -0.18122000

H -0.69199600 1.56695600 1.35440300

H 0.69185000 1.56722800 -1.35407600

H 1.33845300 2.15092500 0.18153400

C 2.59580900 -0.27759000 0.08721100

C -2.59573800 -0.27755600 -0.08777400

H 2.95747500 -1.28993100 0.23310700

H 3.34144000 0.51164300 0.05763800

H -2.95749300 -1.28995800 -0.23292400

H -3.34133400 0.51173000 -0.05833400

24H

E = -575.341792125

C -1.31295000 0.00827700 0.05600800

C -0.72189400 1.35004700 0.41734000

C 0.66060600 1.40605800 -0.24689500

C 1.30372600 0.03989200 -0.09740000

P 0.00895500 -1.27758100 0.03794400

H -1.36735000 2.17620700 0.10809200

H -0.60227800 1.40494700 1.50626500

H 0.53317700 1.61783600 -1.31527100

H 1.29607700 2.19073200 0.17068000

H 0.09299900 -1.42179600 1.45080900

C 2.61113800 -0.18978300 -0.08238700

C -2.57438800 -0.21723800 -0.29148400

H 3.01943100 -1.19204000 -0.00413500

H 3.32698300 0.62540600 -0.14382700

H -2.92103300 -1.20408700 -0.57940100

H -3.30975100 0.58298900 -0.29347400

25

E = -614.035464454

C 1.40991000 -0.24129100 0.05241500

C 1.26413700 1.25501300 0.22559300

C -0.00001000 1.82928200 -0.41147100

C -1.26411700 1.25499500 0.22568500

C -1.40993500 -0.24130100 0.05250000

P 0.00000100 -1.35446600 0.00268200

H -0.00005800 1.62195300 -1.48744300

H -0.00003500 2.91659800 -0.29431700

H -2.15134500 1.74733800 -0.18353500

H -1.24639200 1.47942600 1.29995000

H 2.15134300 1.74733200 -0.18368000

H 1.24649200 1.47949500 1.29986800

C -2.62769100 -0.80461700 -0.07193100

C 2.62769400 -0.80462500 -0.07192700

H -3.53205100 -0.20226600 -0.07049000

H -2.75272600 -1.87766200 -0.17785800

H 3.53208400 -0.20230300 -0.07013100

H 2.75273500 -1.87766900 -0.17777500

25H

E = -614.657244951

C 1.38156000 -0.23994200 -0.02179300

C 1.28083400 1.23566300 0.28147700

C 0.00017300 1.86209700 -0.28957300

C -1.28064400 1.23582200 0.28131900

C -1.38154400 -0.23980300 -0.02191400

P -0.00008900 -1.23792700 0.67040500

H 0.00023400 1.74627400 -1.37864800

H 0.00023800 2.93515100 -0.07668100

H -2.15827900 1.74783700 -0.12341800

H -1.29384800 1.38592500 1.36905000

H 2.15858300 1.74756500 -0.12317100

H 1.29393500 1.38575300 1.36920500

H -0.00011300 -2.28158200 -0.28646200

C -2.40087500 -0.77103700 -0.69023700

C 2.40073700 -0.77130200 -0.69025000

H -3.22675300 -0.15267700 -1.03182800

H -2.45683600 -1.83058600 -0.91773800

H 3.22625100 -0.15291200 -1.03270100

H 2.45648400 -1.83083200 -0.91784600

26

E = -653.340767728

C -2.37219500 -1.40451800 -0.30784100

C -1.29685000 -0.72528900 0.13000900

C -1.45523700 0.58108400 0.87081300

C -1.19254500 1.81133700 -0.00657700

C 0.13232300 1.76178700 -0.77298200

C 1.31789500 1.28923000 0.07832300

C 1.55278500 -0.21414500 0.09511500

C 2.81311500 -0.68046300 0.20677600

P 0.30846700 -1.50091500 -0.14871500

H -2.01494800 1.92704900 -0.71979800

H 0.34330500 2.76079200 -1.16457000

H -2.46796200 0.64327600 1.28060500

H 1.19166100 1.64527200 1.10812200

H 0.03499900 1.10835000 -1.64594900

H -1.20556700 2.69721800 0.63846100

H -0.77557000 0.59139900 1.72891200

H 2.23781600 1.75442400 -0.28690300

H -2.27817100 -2.34403900 -0.84356100

H -3.37950200 -1.03592600 -0.13386500

H 3.03083900 -1.74370300 0.22136000

H 3.66034300 -0.00452800 0.28610300

26H

E = -653.966264798

C 1.43825900 -0.44244700 0.02727700

P 0.00685900 -1.45266400 -0.54216800

C -1.39094500 -0.46617500 0.17247200

C -1.06566500 0.66060600 1.12085800

C -0.58778200 1.93234200 0.38151100

C 0.28825000 1.68787100 -0.85120900

C 1.59624900 0.92686900 -0.60373400

H -1.47058600 2.48982400 0.05226700

H 0.09495000 -2.41553600 0.49528700

H 0.53550300 2.65495900 -1.29928000

H -1.95143900 0.90840600 1.71352300

H 2.26185000 1.52594200 0.02706900

H -0.30251900 1.15580300 -1.60586700

H -0.05691900 2.57498300 1.09234900

H -0.29366900 0.33107700 1.82127100

C -2.63299900 -0.75775000 -0.20592200

H 2.10231500 0.80400900 -1.56877100

C 2.33245000 -0.91607600 0.89317800

H -2.84673100 -1.53460600 -0.93343500

H -3.48581300 -0.22926200 0.21243800

H 2.24001100 -1.89815100 1.34436700

H 3.20326200 -0.32891900 1.17470400

27

E = -573.496315193

C -1.27395900 0.05100600 0.00006700

P -0.00034800 -1.23185300 0.00009200

C 1.27392500 0.05021000 -0.00009800

C 0.66947000 1.38945700 0.00001100

C -0.66889400 1.38986600 -0.00002500

H -1.28084600 2.28524500 0.00008600

C -2.59985000 -0.18658200 -0.00012600

H 1.28207800 2.28440400 0.00020900

C 2.59993200 -0.18687400 -0.00010700

H 2.99913000 -1.19509400 -0.00044000

H 3.31763900 0.62801000 0.00052100

H -2.99839200 -1.19506600 -0.00004900

H -3.31814700 0.62779800 -0.00003600

27H

E = -574.117401392

C 1.30712600 0.08164900 -0.01276400

P 0.00013400 -1.22836300 -0.02139300

C -1.30710700 0.08137200 -0.01296700

C -0.67020700 1.39893800 0.05103700

C 0.66998300 1.39908600 0.05109300

H 1.26945800 2.30338600 0.06532600

C 2.62012700 -0.13532600 -0.09500800

H -1.26993400 2.30308500 0.06514800

C -2.62016400 -0.13544600 -0.09499000

H 0.00004700 -1.43545100 1.38426000

H -3.03472800 -1.13388000 -0.17713900

H -3.32554300 0.69026700 -0.07870900

H 3.03446800 -1.13383400 -0.17749700

H 3.32567700 0.69024000 -0.07890500

28

E = -612.801593277

C -1.41383200 -0.16296400 0.01868100

P -0.04207800 -1.32491700 -0.06656000

C 1.37606400 -0.22648000 -0.01810600

C 1.27134900 1.26221000 -0.27555700

C -0.03843900 1.90805600 0.07043200

C -1.20754100 1.28115100 0.18624700

H -0.01399800 2.98667600 0.19445700

H 1.45988400 1.43751200 -1.34433300

H 2.08068700 1.77536000 0.25374600

C 2.57809900 -0.78801500 0.21431000

H -2.09760500 1.86518600 0.40777000

C -2.66414300 -0.67081700 -0.03336700

H 2.68777100 -1.85696100 0.36586900

H 3.48475300 -0.19078100 0.25851600

H -2.84316900 -1.73383900 -0.15530900

H -3.53650400 -0.02823600 0.04185000

28H

E = -613.426584762

C 1.40231300 -0.08671100 -0.09588900

P 0.10513700 -1.26575100 0.47962200

C -1.36044900 -0.28762000 -0.04408900

C -1.38709500 1.17750900 0.30562900

C -0.09330700 1.90445600 0.04700500

C 1.10542900 1.34911800 -0.14109400

H -0.16822500 2.98757100 0.01047200

H -1.63824700 1.28903000 1.37047200

H -2.19210500 1.67389600 -0.24736800

C -2.32953900 -0.88298100 -0.73312800

H 1.95129200 2.00158200 -0.34738200

C 2.60152200 -0.56588300 -0.43709400

H 0.10173200 -0.81126100 1.82905800

H -2.28864200 -1.94069400 -0.97140200

H -3.19899000 -0.33122700 -1.08226600

H 2.81784300 -1.62849700 -0.41935700

H 3.40504200 0.09853600 -0.74459800

29

E = -652.105786561

C -2.54630500 -1.14513200 -0.23329800

C -1.37938300 -0.56200500 0.10584600

C -1.36368100 0.77828100 0.79573200

C -0.89473400 1.92495800 -0.10441400

C 0.58160600 2.01642600 -0.35562100

C 1.53980200 1.09335100 -0.25518100

C 1.49140100 -0.35426100 0.04076800

C 2.64392900 -0.96244400 0.38915200

P 0.10909100 -1.49763800 -0.24482300

H -1.42738200 1.87843400 -1.06286700

H 0.91885000 3.01170000 -0.63674800

H -2.37665600 0.99988500 1.14548100

H 2.55407400 1.44518500 -0.43638400

H -2.58086800 -2.10741400 -0.73447300

H -3.49912200 -0.67225700 -0.01244200

H 2.69446400 -2.03059400 0.57251300

H 3.56785500 -0.40183000 0.50114000

H -0.72144500 0.73642100 1.68159000

H -1.20194700 2.87000700 0.35663000

29H

E = -652.732781656

C 1.36465400 -0.64508700 0.10007100

P -0.18248400 -1.26782300 -0.69032900

C -1.48562800 -0.28516800 0.16165600

C -1.18162900 1.08869000 0.70360700

C -0.40253100 2.01000300 -0.24952400

C 1.09693400 1.85159200 -0.23908700

C 1.82431900 0.74843000 -0.04940200

H -0.78869200 1.89732700 -1.27071300

H 1.65146000 2.77807500 -0.36578100

H -2.12619800 1.56916600 0.97733200

C -2.69174900 -0.83935000 0.27121100

H 2.90479600 0.87160000 -0.00047500

C 2.12853300 -1.54453100 0.72858300

H -2.89287800 -1.84559500 -0.08123700

H -3.52239600 -0.30077000 0.72085600

H 1.83252700 -2.58437600 0.81390400

H 3.07562500 -1.26182000 1.18144500

H -0.60394300 0.98143800 1.62933600

H -0.61828100 3.04530900 0.02933400

H -0.09216300 -0.44048100 -1.84174900

30

E = -652.100119222

C 1.31052700 -0.63274000 0.03859000

C 1.75285000 0.66958000 -0.59393300

C 0.84125900 1.86381900 -0.49200900

C -0.31451300 1.98566500 0.15235400

C -1.02364500 0.91767800 0.95709100

C -1.45617500 -0.25954400 0.10881200

P -0.24515900 -1.42879600 -0.52070300

H 1.20394000 2.73406700 -1.03437700

H -1.90223200 1.35400000 1.43850300

H 1.92487300 0.48219100 -1.66289700

C 2.08865500 -1.27966800 0.90708000

H -0.82447200 2.94239600 0.08493400

H -0.36704900 0.56020400 1.75658100

C -2.75485500 -0.48553100 -0.17188500

H 2.73387900 0.94561700 -0.18647600

H 3.03602700 -0.85940600 1.23657100

H 1.81139400 -2.24403800 1.32048000

H -3.53688500 0.16739200 0.20461300

H -3.06671000 -1.32604400 -0.78397900

30H

E = -652.727479104

C 1.34972100 -0.54341500 -0.02821900

C 1.52986700 0.69090500 -0.88161300

C 0.71309000 1.89684900 -0.48354900

C -0.35145300 1.97480300 0.30989100

C -1.07366800 0.85144400 1.02984900

C -1.44991700 -0.28927200 0.11001600

P -0.16616000 -1.53082500 -0.34643300

H 1.06157000 2.82455300 -0.93181900

H -1.98277300 1.25702700 1.48175100

H 1.30295600 0.45279700 -1.93025900

C 2.27838800 -0.92456900 0.84577500

H -0.78410600 2.96143700 0.45128600

H -0.45089200 0.47764200 1.85002000

C -2.67286600 -0.42766000 -0.39576000

H -0.15080300 -2.22645300 0.89098000

H 2.58685000 0.97835600 -0.86603900

H 3.20552800 -0.36844400 0.95697100

H 2.15687900 -1.80102800 1.47365100

H -3.47584000 0.25206400 -0.12239000

H -2.91594200 -1.22008700 -1.09599000

31

E = -650.873467408

C 1.48054700 -0.40698800 0.00775500

P 0.00000600 -1.43319500 0.00022900

C -1.48053800 -0.40703200 -0.00781600

C -1.62273300 1.04467600 0.14039400

C -0.72078900 2.03459600 0.10008900

C 0.72077900 2.03461800 -0.10007800

C 1.62274600 1.04471500 -0.14035800

H -1.13428800 3.03259000 0.21818000

H 1.13424400 3.03263300 -0.21810500

H -2.65523300 1.35628500 0.28619100

C -2.61938400 -1.13384600 -0.11484900

H 2.65525600 1.35632500 -0.28609300

C 2.61937000 -1.13388200 0.11441400

H -2.60154600 -2.21329800 -0.22170000

H -3.59466000 -0.65717100 -0.10036100

H 2.60148600 -2.21332600 0.22131400

H 3.59467200 -0.65726100 0.09983200

31H

E= -651.501065063

C 1.42605500 -0.42599400 0.09359800

P -0.00002400 -1.10782000 -0.84180700

C -1.42599900 -0.42583000 0.09372700

C -1.63691800 1.02351000 0.06545800

C -0.73075200 2.01116800 -0.03110600

C 0.73096600 2.01110100 -0.03142200

C 1.63712300 1.02333100 0.06510800

H -1.14884700 3.01400000 -0.04513900

H -0.00005100 -2.39181800 -0.24481900

H 1.14911700 3.01391400 -0.04550900

H -2.67750000 1.32931800 0.14774500

C -2.34865300 -1.22775400 0.63669600

H 2.67776300 1.32902900 0.14707600

C 2.34828100 -1.22799500 0.63722500

H -2.22815500 -2.30446800 0.67852600

H -3.25854700 -0.81840400 1.06706500

H 2.22761300 -2.30468900 0.67910200

H 3.25835700 -0.81880400 1.067368

2tBu

E= -733.179038

C	0.152413	0.274518	-0.070942
C	0.056672	0.108199	1.449303
C	1.466625	-0.010755	2.050196
C	-0.710562	-1.120421	1.820178
P	-1.282266	-2.047449	3.284943
C	-1.380198	-2.186726	1.467590
C	-1.939186	-3.069949	0.398407
C	-3.463260	-3.140329	0.586655
C	-1.612820	-2.536144	-1.000346
C	-1.335302	-4.472598	0.574741
C	-0.661254	1.321639	2.061952
H	2.044098	0.891247	1.827858
H	1.419397	-0.132345	3.135108
H	1.997913	-0.870974	1.634115
H	-0.103427	2.236074	1.840244
H	-1.670168	1.426331	1.653910
H	-0.741958	1.220391	3.146980
H	0.717751	1.179707	-0.311432
H	0.662948	-0.575418	-0.529588
H	-0.838762	0.364677	-0.521313
H	-2.033660	-3.201332	-1.760101
H	-2.035088	-1.539968	-1.151065
H	-0.533504	-2.480265	-1.159557
H	-1.752733	-5.154856	-0.171444
H	-0.249283	-4.449386	0.450684
H	-1.555750	-4.870203	1.568403
H	-3.900583	-3.810548	-0.159047
H	-3.716658	-3.516756	1.580709
H	-3.917782	-2.152750	0.470629

{2tBu}$_2$

E= -1466.429161

C	-2.244538	0.647548	-0.231298
C	-2.244436	-0.647544	-0.231315
P	-0.653243	0.000133	-0.923113
P	0.653253	0.000097	0.923105
C	2.244517	0.647580	0.231289
C	2.244472	-0.647514	0.231311
C	-2.922480	1.954632	0.025224
C	-2.922187	-1.954733	0.025153
C	2.922275	-1.954679	-0.025157
C	2.922394	1.954701	-0.025221
C	-4.299064	-1.742130	0.663666
C	-3.073735	-2.682551	-1.319852
C	-2.035904	-2.788621	0.963247
C	-4.299286	1.741812	0.663814
C	-2.036291	2.788663	0.963283
C	-3.074210	2.682429	-1.319774
C	4.299160	-1.742039	-0.663638
C	3.073814	-2.682507	1.319843
C	2.036036	-2.788584	-0.963276
C	4.299191	1.741979	-0.663863
C	2.036132	2.788715	-0.963224
C	3.074131	2.682464	1.319795
H	-2.499873	-3.761837	1.149281
H	-1.051739	-2.957445	0.518772
H	-1.891435	-2.283096	1.921555
H	-3.528347	-3.665771	-1.166149
H	-3.709091	-2.110974	-2.001611
H	-2.100639	-2.821760	-1.797753
H	-4.782489	-2.707476	0.841402

H	-4.211035	-1.223301	1.621276
H	-4.947841	-1.152493	0.011883
H	-4.782809	2.707088	0.841662
H	-4.948044	1.152147	0.012036
H	-4.211118	1.222921	1.621375
H	-2.500423	3.761794	1.149351
H	-1.891688	2.283152	1.921578
H	-1.052177	2.957674	0.518770
H	-3.529014	3.665557	-1.166055
H	-2.101153	2.821840	-1.797696
H	-3.709464	2.110730	-2.001524
H	2.500035	-3.761787	-1.149306
H	1.051866	-2.957438	-0.518825
H	1.891575	-2.283058	-1.921585
H	3.528462	-3.665710	1.166140
H	3.709135	-2.110917	2.001624
H	2.100711	-2.821753	1.797721
H	4.782610	-2.707374	-0.841373
H	4.211142	-1.223204	-1.621245
H	4.947911	-1.152396	-0.011836
H	4.782651	2.707290	-0.841700
H	4.948007	1.152334	-0.012126
H	4.211020	1.223113	-1.621437
H	3.528884	3.665619	1.166093
H	2.101081	2.821814	1.797750
H	3.709432	2.110773	2.001509
H	2.500202	3.761880	-1.149272
H	1.891533	2.283229	-1.921533
H	1.052021	2.957652	-0.518677

8tBu

E= -810.614409

C	0.188185	0.133997	-0.019773
C	-0.049678	0.127787	1.500788
C	1.265397	0.473140	2.209248
C	-0.556444	-1.238465	1.928607
C	0.019693	-2.089915	2.809256
C	-0.710057	-3.340128	3.017790
C	-1.850916	-3.456177	2.298622
C	-2.822684	-4.623238	2.292988
C	-4.212143	-4.136327	2.741255
P	-2.082159	-1.970925	1.294931
C	-2.924643	-5.199207	0.869405
C	-2.355994	-5.731962	3.243531
C	-1.099277	1.197289	1.851778
H	-0.341836	-4.097305	3.701611
H	0.947676	-1.887823	3.333198
H	-3.612350	-6.050632	0.851377
H	-3.297347	-4.451690	0.163149
H	-1.946806	-5.539089	0.517256
H	-4.922311	-4.969243	2.752426
H	-4.167807	-3.708415	3.746555
H	-4.603253	-3.370885	2.064786
H	-3.067619	-6.562207	3.226413
H	-1.378056	-6.123253	2.948935
H	-2.287852	-5.370531	4.273555
H	1.614761	1.458396	1.888017
H	1.136809	0.501103	3.295037
H	2.047841	-0.253309	1.972070
H	0.563730	1.110211	-0.342536
H	0.921266	-0.626990	-0.301226
H	-0.736369	-0.068003	-0.568323

H	-0.742609	2.190724	1.561840
H	-2.043620	1.014448	1.330915
H	-1.301891	1.204480	2.926308

{8tBu}$_2$

E= -1621.314310

C	2.302778	-0.786761	-0.539623
P	0.562953	-0.545494	-0.991464
P	-0.564023	-0.597787	0.911218
C	-0.851036	1.054035	1.613703
C	-2.182341	1.284546	1.565809
C	-2.981440	0.274169	0.898432
C	-2.299968	-0.780154	0.399005
C	3.001452	0.227723	-1.098246
C	2.209986	1.267987	-1.719400
C	0.867088	1.110751	-1.664980
H	2.687059	2.106410	-2.215950
C	-0.157889	2.069078	-2.257765
H	4.084702	0.268967	-1.117148
C	2.890178	-1.995461	0.176523
H	-4.060950	0.365740	0.851207
C	-2.904441	-1.994672	-0.296736
H	-2.652215	2.141526	2.035876
C	0.167249	1.839236	2.426203
C	-0.343895	3.258240	2.711818
C	0.380567	1.111190	3.768321
C	1.514126	1.937662	1.700492
H	0.418835	3.818957	3.259285
H	-0.559785	3.797303	1.785539
H	-1.248201	3.250188	3.326136
H	2.233455	2.477393	2.324110
H	1.932684	0.951300	1.491165
H	1.419207	2.464540	0.750970
H	1.079237	1.674533	4.395204
H	-0.564316	1.006756	4.308188
H	0.795068	0.111697	3.612447
C	-4.332367	-2.241790	0.214400
C	-2.960206	-1.762143	-1.817265
C	-2.063621	-3.250856	-0.021458
H	-4.739871	-3.145716	-0.247919
H	-4.345311	-2.374356	1.299440
H	-5.002825	-1.416635	-0.039757
H	-2.535169	-4.124267	-0.481332
H	-1.061907	-3.156442	-0.448786
H	-1.964612	-3.438172	1.051108
H	-3.425735	-2.622544	-2.308558
H	-3.547298	-0.871058	-2.054496
H	-1.959449	-1.632954	-2.234627
C	2.032529	-3.246315	-0.068633
C	4.316358	-2.275169	-0.320451
C	2.943822	-1.715651	1.690673
C	-1.501183	1.369732	-2.488978
C	-0.373364	3.238314	-1.281164
C	0.342820	2.623909	-3.601227
H	-2.228411	2.080909	-2.892403
H	-1.402387	0.546167	-3.201333
H	-1.909436	0.972681	-1.556869
H	-0.414075	3.284194	-4.035783
H	1.258821	3.208471	-3.484433
H	0.542235	1.814780	-4.308650
H	-1.086653	3.955278	-1.700849
H	-0.772106	2.875797	-0.331881
H	0.566013	3.763828	-1.087036

H	3.387059	-2.568319	2.215007
H	3.550861	-0.830696	1.900687
H	1.943295	-1.552034	2.098639
H	2.494352	-4.116566	0.406962
H	1.033618	-3.130389	0.359366
H	1.928329	-3.452489	-1.137253
H	4.709634	-3.171891	0.167366
H	4.332324	-2.436774	-1.401543
H	4.996746	-1.452505	-0.085128

12tBu

E= -849.921080

C	-0.045272	0.240851	-0.125547
C	0.079549	0.001122	1.383826
C	1.574515	-0.143045	1.723472
C	-0.672360	-1.261271	1.845892
C	-1.354542	-2.042172	0.989690
C	-2.116388	-3.281282	1.320159
C	-2.142395	-3.723533	2.744501
C	-1.544128	-3.121984	3.787805
C	-1.642596	-3.676650	5.221378
C	-2.292859	-2.619920	6.133278
P	-0.569744	-1.611441	3.614516
C	-0.493282	1.230594	2.112739
C	-2.483246	-4.956602	5.300556
C	-0.229431	-3.999229	5.740963
H	-1.730141	-4.107204	0.702245
H	-2.715768	-4.630739	2.911306
H	-1.379938	-1.779995	-0.063980
H	-3.153654	-3.160637	0.970036
H	2.124170	0.741707	1.387787
H	1.735459	-0.250667	2.798696
H	2.000839	-1.020594	1.229116
H	0.506259	1.145950	-0.394205
H	0.375293	-0.585764	-0.705384
H	-1.085652	0.386224	-0.430260
H	0.022331	2.137977	1.783497
H	-1.559908	1.344750	1.899418
H	-0.373016	1.149902	3.195622
H	-2.381930	-3.006019	7.153370
H	-1.702094	-1.701721	6.171904
H	-3.293509	-2.362304	5.774705
H	-0.284812	-4.407351	6.754851
H	0.259519	-4.737823	5.099369
H	0.402359	-3.108426	5.771364
H	-2.517531	-5.302498	6.337260
H	-3.513763	-4.787802	4.975095
H	-2.055317	-5.762998	4.698086

$\{12tBu\}_2$

E= -1699.910771

C	-1.401790	-1.785800	0.463666
P	-0.000186	-1.029327	-0.479773
P	-0.000022	1.029430	0.479959
C	1.401806	1.785644	-0.463344
C	1.259843	1.976976	-1.774782
C	0.000229	1.660318	-2.540931
C	-1.259452	1.977121	-1.774955
C	-1.401641	1.785819	-0.463543
C	-1.259612	-1.977243	1.775063
C	0.000114	-1.660483	2.540993
C	1.259663	-1.977300	1.774791

C	1.401625	-1.785788	0.463376
H	0.000149	0.595234	-2.811731
C	2.582752	2.311656	0.360208
H	-2.061830	2.427873	-2.352245
C	-2.582609	2.312154	0.359737
H	2.062353	2.427630	-2.351953
H	0.000319	2.213997	-3.482938
H	0.000180	-2.213997	3.483092
C	-2.582769	-2.311955	-0.359779
H	2.062106	-2.428144	2.351899
C	2.582530	-2.311844	-0.360262
H	-2.061939	-2.428091	2.352336
H	0.000208	-0.595352	2.811609
C	-3.058888	-1.292711	-1.407834
C	-2.094166	-3.572279	-1.101491
C	-3.774624	-2.695432	0.525881
C	3.058086	-1.292647	-1.408644
C	3.774813	-2.694794	0.525080
C	2.094054	-3.572424	-1.101559
C	3.774884	2.694844	-0.525258
C	2.094288	3.572097	1.101765
C	3.058550	1.292381	1.408418
C	-3.774453	2.695435	-0.526045
C	-3.058800	1.293225	1.408081
C	-2.093977	3.572684	1.101138
H	-4.607087	-3.026937	-0.101044
H	-3.528952	-3.516853	1.204215
H	-4.118786	-1.847430	1.124856
H	-3.860380	-1.732165	-2.009453
H	-3.444574	-0.383465	-0.944810
H	-2.250885	-1.002922	-2.083230

H	-2.904520	-3.997561	-1.702362
H	-1.265693	-3.334518	-1.774763
H	-1.751429	-4.329816	-0.391718
H	3.859282	-1.732077	-2.010667
H	2.249674	-1.003010	-2.083623
H	3.443849	-0.383286	-0.945899
H	2.904424	-3.997867	-1.702299
H	1.751347	-4.329762	-0.391557
H	1.265551	-3.334949	-1.774904
H	4.607029	-3.026452	-0.102094
H	4.119098	-1.846489	1.123535
H	3.529573	-3.515980	1.203846
H	4.607365	3.025926	0.101868
H	4.118829	1.846800	-1.124289
H	3.529612	3.516473	-1.203483
H	3.859352	1.732032	2.010803
H	2.250092	1.002190	2.083104
H	3.444923	0.383359	0.945510
H	2.904667	3.997400	1.702582
H	1.751592	4.329572	0.391904
H	1.265777	3.334496	1.775057
H	-3.859391	1.733334	2.010414
H	-3.445637	0.384329	0.945311
H	-2.250479	1.002701	2.082784
H	-2.904672	3.998798	1.700955
H	-1.266274	3.334849	1.775335
H	-1.750140	4.329589	0.391222
H	-4.607176	3.026384	0.100828
H	-3.528968	3.517188	-1.204053
H	-4.118144	1.847506	-1.125381

21tBu

E= -887.987209

C	0.128402	0.173226	-0.048119
C	-0.007313	-0.078722	1.463065
C	1.390329	0.114285	2.081893
C	-0.543274	-1.496213	1.829154
C	-0.638797	-1.765014	3.160154
C	-1.018147	-2.956115	3.873449
C	-1.625281	-4.085250	3.454506
C	-2.098252	-4.478539	2.153282
C	-1.872213	-3.966163	0.912677
C	-2.520728	-4.748331	-0.269760
C	-4.051176	-4.725605	-0.098571
P	-0.903862	-2.536344	0.405524
C	-2.207229	-4.166016	-1.658353
C	-2.009723	-6.201740	-0.262063
C	-0.981126	0.977709	2.017769
H	-1.823957	-4.820982	4.229562
H	-2.709609	-5.376752	2.195639
H	-0.336851	-0.965479	3.832384
H	-0.798552	-2.913905	4.937164
H	1.785466	1.097941	1.810553
H	1.374662	0.051739	3.171684
H	2.082681	-0.646195	1.710501
H	-0.625645	1.981797	1.766677
H	-1.975894	0.848110	1.582684
H	-1.079123	0.918697	3.103775
H	0.507029	1.188085	-0.200432
H	0.828443	-0.519658	-0.520650
H	-0.828679	0.090192	-0.567591
H	-2.705668	-4.779005	-2.414863
H	-2.567168	-3.140570	-1.766300
H	-1.136637	-4.170997	-1.875694
H	-2.441243	-6.752981	-1.103050
H	-0.920982	-6.226151	-0.360346
H	-2.276907	-6.731447	0.654358
H	-4.527313	-5.265634	-0.922754
H	-4.368759	-5.192704	0.836018
H	-4.422298	-3.697063	-0.103715

{21tBu}$_2$

E= -1776.075607

P	0.002305	-1.057507	0.402562
C	1.443771	-1.776627	-0.514403
C	1.583133	-1.727462	-1.851533
C	0.675380	-1.228584	-2.861668
C	-0.669982	-1.231459	-2.861615
C	-1.575537	-1.734439	-1.851449
C	-1.435944	-1.782988	-0.514330
C	-2.498599	-2.449975	0.379362
C	-3.715883	-2.940702	-0.414918
P	-0.002362	1.057497	-0.402570
C	1.435871	1.782877	0.514388
C	2.498635	2.449789	-0.379246
C	3.715974	2.940347	0.415064
C	2.509363	-2.438866	0.379328
C	3.728601	-2.924755	-0.414934
C	-1.443740	1.776689	0.514325
C	-2.509266	2.439051	-0.379422
C	-3.004137	1.447769	-1.447397
C	-1.583272	1.727439	1.851448
C	-0.675630	1.228619	2.861620

C	0.669749	1.231441	2.861609
C	1.575364	1.734309	1.851531
C	1.866415	3.662925	-1.085121
C	2.997500	1.460767	-1.447484
C	-3.728522	2.924960	0.414781
C	-1.881917	3.654539	-1.085631
C	3.004251	-1.447414	1.447139
C	1.882136	-3.654297	1.085699
C	-1.866216	-3.663009	1.085291
C	-2.997556	-1.460975	1.447569
H	2.513523	-2.096082	-2.275160
H	-2.504218	-2.107278	-2.275130
H	1.157846	-0.898624	-3.778554
H	-2.513760	2.095978	2.274927
H	2.504118	2.106929	2.275242
H	1.153627	0.903614	3.778515
H	4.435671	3.392514	-0.272637
H	3.443954	3.699321	1.153624
H	4.222920	2.118195	0.929060
H	3.742054	1.948183	-2.084560
H	3.460328	0.583082	-0.988825
H	2.180691	1.114630	-2.084227
H	2.620571	4.183459	-1.683707
H	1.064597	3.351586	-1.756798
H	1.454702	4.368254	-0.358043
H	-3.750727	1.932025	-2.084505
H	-2.185965	1.104878	-2.084133
H	-3.463416	0.568329	-0.988506
H	-2.638101	4.171839	-1.684452
H	-1.473094	4.361760	-0.358759
H	-1.078781	3.346214	-1.757121
H	-4.449998	3.374200	-0.272974
H	-4.232230	2.100940	0.928967
H	-3.459445	3.685120	1.153204
H	-4.435525	-3.392931	0.272798
H	-4.222903	-2.118614	-0.928941
H	-3.443772	-3.699668	-1.153453
H	-3.742036	-1.948477	2.084669
H	-2.180776	-1.114738	2.084287
H	-3.460517	-0.583367	0.988890
H	-2.620298	-4.183626	1.683899
H	-1.454401	-4.368315	0.358252
H	-1.064454	-3.351518	1.756968
H	3.750975	-1.931520	2.084201
H	3.463345	-0.567960	0.988099
H	2.186127	-1.104528	2.083945
H	2.638356	-4.171429	1.684621
H	1.078937	-3.345967	1.757112
H	1.473419	-4.361668	0.358914
H	4.450170	-3.373876	0.272803
H	3.459542	-3.685017	-1.153259
H	4.232181	-2.100734	-0.929237
H	-1.158113	0.898778	3.778537
H	-1.153948	-0.903555	-3.778444

2TMS

E= -1236.041253

C	-0.153559	0.377156	-0.240243
Si	-0.276817	0.059824	1.604563
C	1.419392	-0.266169	2.304111
P	2.306083	-0.793450	3.821643
C	2.720423	-0.282787	2.108553

Si	4.142416	-0.007648	0.936105
C	3.517305	0.779979	-0.647580
C	-1.322463	-1.456327	1.948960
C	-0.963771	1.558649	2.496464
C	5.366577	1.118275	1.798833
C	4.907926	-1.683873	0.592288
H	-1.986620	1.769716	2.170267
H	-0.980474	1.394442	3.577253
H	-0.356602	2.446613	2.300668
H	-2.346754	-1.312374	1.592324
H	-0.913193	-2.340129	1.451969
H	-1.362978	-1.663476	3.021720
H	-1.141250	0.587270	-0.662061
H	0.493512	1.231594	-0.454837
H	0.254261	-0.493299	-0.761951
H	4.340244	0.933563	-1.352438
H	2.767791	0.151849	-1.135929
H	3.062023	1.754263	-0.448367
H	6.247075	1.289284	1.172280
H	4.918990	2.090120	2.024225
H	5.701669	0.676737	2.741231
H	5.787783	-1.584533	-0.050474
H	5.220863	-2.166717	1.521957
H	4.196913	-2.346892	0.091949

$\{2TMS\}_2$

E= -2472.155795

C	-2.113320	-0.550349	-0.403610
C	-2.271640	0.740916	-0.326222
P	-0.565904	0.328988	-0.954066
P	0.588158	0.329418	0.983819
C	2.295166	0.728732	0.349249
C	2.127949	-0.560924	0.428945
Si	-2.777434	-2.263261	-0.125921
Si	-3.327593	2.201010	0.129370
Si	2.741261	-2.287843	0.124714
Si	3.362282	2.172401	-0.131955
C	4.546893	-2.238092	-0.379916
C	2.513900	-3.261317	1.710032
C	1.672801	-2.993492	-1.246443
H	4.930903	-3.246432	-0.562199
H	4.684096	-1.654451	-1.294189
H	5.158647	-1.783592	0.404523
H	2.747709	-4.319103	1.556476
H	3.166469	-2.881773	2.501098
H	1.482486	-3.190041	2.066262
H	1.891334	-4.052593	-1.413223
H	0.612293	-2.900088	-0.997121
H	1.835456	-2.457804	-2.185551
C	4.903252	1.535701	-0.992400
C	2.338200	3.244667	-1.277614
C	3.802355	3.107057	1.432394
H	2.903768	4.121366	-1.607549
H	2.024750	2.686555	-2.164069
H	1.435231	3.596128	-0.770259
H	4.359257	4.019410	1.198276
H	2.899087	3.392627	1.978557
H	4.418095	2.495092	2.097064
H	5.566407	2.360028	-1.271960
H	5.464320	0.857514	-0.343550
H	4.642625	0.990636	-1.904022
C	-4.577298	-2.156431	0.390629

C	-2.595699	-3.213490	-1.731361
C	-1.732556	-3.044591	1.221825
H	-2.056771	-4.070725	1.420582
H	-0.679413	-3.075878	0.929699
H	-1.800818	-2.477269	2.153833
H	-5.000927	-3.153335	0.546100
H	-4.687502	-1.594163	1.321747
H	-5.173322	-1.655132	-0.377258
H	-2.882810	-4.260989	-1.598647
H	-3.226775	-2.786422	-2.515541
H	-1.561139	-3.188290	-2.084893
C	-2.304365	3.274426	1.274745
C	-3.735718	3.121814	-1.451881
C	-4.887788	1.596962	0.978482
H	-5.538125	2.435530	1.245737
H	-5.455220	0.924674	0.329169
H	-4.646495	1.053897	1.896557
H	-4.280979	4.045687	-1.236431
H	-2.821848	3.386072	-1.991122
H	-4.353066	2.510435	-2.115610
H	-2.860868	4.165458	1.580918
H	-2.015069	2.726256	2.175465
H	-1.387604	3.602789	0.776782

8TMS

E= -1313.477716

C	-0.205583	-0.040864	0.029850
Si	-0.050408	-0.021493	1.901712
C	1.760582	0.000999	2.375624
C	2.361850	0.921883	3.179728
C	3.790177	0.703063	3.400930
C	4.305948	-0.387253	2.767442
Si	6.092120	-0.944682	2.827850
C	6.841018	-0.823071	1.109277
C	-0.860311	-1.561369	2.610279
C	-0.872931	1.517038	2.599579
P	2.990416	-1.201406	1.850733
C	7.028422	0.167812	4.017975
C	6.170035	-2.726504	3.417269
H	4.373648	1.372582	4.027334
H	1.842579	1.762344	3.632566
H	7.892027	-1.128175	1.116473
H	6.310614	-1.467904	0.402252
H	6.788742	0.201447	0.730129
H	7.205255	-3.078719	3.462162
H	5.734718	-2.829833	4.415184
H	5.620761	-3.389891	2.742546
H	8.080654	-0.127261	4.070745
H	6.993607	1.214311	3.701037
H	6.614463	0.105715	5.028625
H	-1.937018	1.531202	2.345493
H	-0.792485	1.555739	3.689928
H	-0.421878	2.427298	2.193874
H	-1.256549	-0.054611	-0.275183
H	0.262865	0.842670	-0.412777
H	0.277423	-0.924828	-0.397173
H	-1.925846	-1.594112	2.362655
H	-0.396522	-2.468675	2.211882
H	-0.765073	-1.588829	3.699455

{8TMS}$_2$

E= -2627.048571

C	-0.587190	-3.747329	2.858894	H	-0.067283	-4.279916	2.057486
Si	-1.199102	-2.069086	2.275880	H	0.095505	-3.659780	3.709052
C	-2.398626	-2.284094	0.855397	H	-3.286071	-2.831743	1.187830
C	0.291858	-1.057179	1.751758	H	-2.724086	-1.318315	0.459913
C	1.571355	-1.298003	2.146147	H	-1.942282	-2.835363	0.030227
C	2.557507	-0.307937	1.753040	H	-2.905787	-1.754414	4.060753
C	2.079398	0.741313	1.034563	H	-1.367424	-1.004356	4.519320
Si	3.071532	2.212567	0.419523	H	-2.434784	-0.200509	3.361365
C	2.013062	3.755002	0.570804	H	5.232448	3.202088	1.139316
P	0.281083	0.591045	1.018325	H	4.376920	2.508550	2.525301
P	-0.222910	0.574619	-1.115387	H	5.239148	1.456291	1.393539
C	-0.276343	-1.054467	-1.878327	H	2.593032	4.650682	0.328261
Si	1.105313	-2.296354	-2.132375	H	1.172338	3.708087	-0.128247
C	0.926910	-3.044753	-3.847479	H	1.607709	3.873419	1.579336
C	-2.000174	0.822707	-1.224861	H	4.064630	2.879112	-1.750306
Si	-3.000163	2.228680	-0.495181	H	4.223862	1.131311	-1.511655
C	-3.442776	1.791599	1.278393	H	2.667865	1.818365	-1.999119
C	-2.478467	-0.154295	-2.042096	H	3.578780	-2.191864	-2.067816
C	-1.526597	-1.184515	-2.402765	H	2.910232	-0.685164	-2.714842
C	2.771600	-1.461108	-1.957364	H	2.870893	-0.993750	-0.973790
C	0.939974	-3.655286	-0.846245	H	1.712133	-3.785640	-4.027106
C	-1.983412	3.805007	-0.509659	H	-0.035581	-3.550725	-3.967841
C	-4.571099	2.431219	-1.504717	H	1.001460	-2.276751	-4.622218
C	4.621997	2.356542	1.470604	H	1.706250	-4.423542	-0.990132
C	3.553779	1.984618	-1.379903	H	1.054570	-3.240458	0.157765
C	-2.056574	-1.173507	3.687239	H	-0.039133	-4.139742	-0.906356
H	-1.812121	-2.004409	-3.056294	H	-4.103099	2.546371	1.716687
H	-3.500271	-0.179700	-2.409688	H	-3.951891	0.824682	1.332733
H	3.600587	-0.412049	2.038153	H	-2.539583	1.735010	1.892771
H	1.855099	-2.165923	2.735369	H	-2.577168	4.656599	-0.163970
H	-1.431916	-4.365937	3.177055	H	-1.122256	3.709889	0.158663

H	-1.610652	4.033470	-1.511834
H	-5.175607	3.254376	-1.112191
H	-4.347283	2.646039	-2.553266
H	-5.184984	1.526250	-1.468677

12TMS

E= -1352.784021

C	0.050761	-0.085112	-0.567693
Si	0.118956	-0.199896	1.307512
C	1.908168	-0.204998	1.878314
C	-0.727757	-1.776206	1.877961
C	-0.766611	1.285725	2.069831
P	-0.808347	1.353968	3.864836
C	-1.713390	2.871852	4.189438
Si	-1.980365	3.316628	6.006943
C	-2.945592	1.937273	6.839303
C	-1.328490	2.229745	1.283774
C	-2.058662	3.455272	1.733918
C	-2.188257	3.670137	3.208617
C	-0.309188	3.507675	6.842270
C	-2.940453	4.928168	6.131011
H	-3.064509	3.454532	1.285193
H	-2.729337	4.577484	3.477277
H	-1.265732	2.125436	0.200536
H	-1.577759	4.339620	1.286948
H	-0.234735	-2.658732	1.458873
H	-0.701305	-1.862289	2.967928
H	-1.776388	-1.794789	1.567750
H	0.563105	-0.945039	-1.009559
H	-0.978630	-0.088369	-0.937732
H	0.543721	0.818606	-0.937648
H	2.450014	-1.058464	1.459320
H	2.423385	0.708565	1.568220
H	1.971068	-0.269238	2.968295
H	-0.427406	3.759767	7.900586
H	0.267744	2.580629	6.780024
H	0.279648	4.298806	6.369420
H	-3.112519	2.160112	7.897621
H	-3.920981	1.796892	6.364843
H	-2.405174	0.988495	6.776799
H	-3.095276	5.186170	7.183000
H	-2.403105	5.758256	5.663256
H	-3.925523	4.851394	5.661621

{12TMS}$_2$

E= -2705.642920

C	-3.536236	-1.141931	-1.568611
Si	-2.886268	-2.378510	-0.312300
C	-4.247456	-2.850790	0.895971
C	-1.406610	-1.704442	0.630820
C	-1.259436	-1.863029	1.950584
C	0.000140	-1.537181	2.713946
C	1.259904	-1.862620	1.950725
C	1.407108	-1.704139	0.630957
Si	2.886930	-2.378056	-0.312033
C	2.301712	-3.907137	-1.236799
P	0.000217	-1.066929	-0.373910
P	-0.000115	1.066838	0.373793
C	-1.407149	1.704050	-0.630875
Si	-2.886863	2.377881	0.312330
C	-2.301579	3.907078	1.236880
C	1.406587	1.704470	-0.631048

Si	2.886254	2.378614	0.312023
C	3.536117	1.142226	1.568569
C	1.259266	1.863175	-1.950778
C	-0.000383	1.537306	-2.714013
C	-1.260057	1.862723	-1.950634
C	4.247525	2.850703	-0.896226
C	2.300698	3.907628	1.236694
C	-4.248369	2.849610	-0.895795
C	-3.536302	1.141403	1.569010
C	-2.300745	-3.907383	-1.237227
C	3.536752	-1.141521	-1.568459
C	4.248161	-2.849927	0.896351
H	-0.000218	0.460231	-2.935491
H	-2.077382	2.272171	-2.543585
H	2.076415	2.272824	-2.543833
H	-0.000528	2.051950	-3.677639
H	0.000162	-2.051846	3.677564
H	2.077177	-2.271991	2.543800
H	-2.076669	-2.272582	2.543587
H	-0.000033	-0.460116	2.935462
H	-5.123033	-3.221101	0.354300
H	-3.921020	-3.644045	1.574837
H	-4.566208	-1.998629	1.503258
H	-4.288019	-1.617822	-2.206313
H	-3.998681	-0.274718	-1.093239
H	-2.730076	-0.776098	-2.210234
H	-3.118941	-4.363589	-1.803141
H	-1.504896	-3.651770	-1.942948
H	-1.906566	-4.656260	-0.544479
H	4.288606	-1.617425	-2.206071
H	2.730580	-0.775853	-2.210146
H	3.999099	-0.274228	-1.093148
H	3.119978	-4.363228	-1.802701
H	1.907718	-4.656027	-0.543958
H	1.505775	-3.651762	-1.942507
H	5.123850	-3.220101	0.354769
H	4.566690	-1.997624	1.503557
H	3.921852	-3.643167	1.575294
H	5.123096	3.221022	-0.354552
H	4.566255	1.998468	-1.503418
H	3.921152	3.643897	-1.575192
H	4.288430	1.618054	2.205698
H	2.730069	0.777130	2.210743
H	3.997915	0.274577	1.093381
H	3.118826	4.363881	1.802666
H	1.906626	4.656430	0.543804
H	1.504746	3.652121	1.942338
H	-4.288589	1.617071	2.206287
H	-3.998023	0.273662	1.093919
H	-2.730062	0.776429	2.211020
H	-3.119784	4.363184	1.802860
H	-1.505546	3.651785	1.942510
H	-1.907702	4.655926	0.543930
H	-5.123947	3.219782	-0.354030
H	-3.922244	3.642845	-1.574833
H	-4.567004	1.997280	-1.502906

21TMS

E= -1390.851231

C	-0.107847	0.633825	-0.309026
Si	0.085423	0.188450	1.503183
C	1.913526	0.153095	1.949482

C	-0.669224	-1.497584	1.912179
C	-0.586374	-1.864368	3.300293
C	-1.129399	-2.894974	4.000932
C	-1.984383	-3.974910	3.576121
C	-2.341315	-4.387464	2.330982
C	-2.000893	-3.897582	1.022450
Si	-2.579943	-5.001998	-0.400403
C	-4.456468	-5.131892	-0.335563
P	-1.249140	-2.412211	0.569075
C	-2.056190	-4.337956	-2.075071
C	-1.837238	-6.712314	-0.148017
C	-0.779041	1.484105	2.558876
H	-2.383052	-4.570259	4.393122
H	-2.990273	-5.264682	2.337179
H	0.003076	-1.185814	3.919004
H	-0.910505	-2.897392	5.065335
H	2.373541	1.131665	1.780232
H	2.069073	-0.108216	3.000350
H	2.448658	-0.581620	1.341425
H	-0.348483	2.475585	2.387514
H	-1.845330	1.532861	2.320822
H	-0.686878	1.261473	3.625964
H	0.308938	1.631208	-0.482339
H	0.415650	-0.068715	-0.962206
H	-1.157780	0.651911	-0.613090
H	-2.380016	-5.032915	-2.856547
H	-2.500293	-3.362065	-2.286451
H	-0.970569	-4.232738	-2.149029
H	-2.176500	-7.402387	-0.926788
H	-0.744947	-6.672302	-0.184262
H	-2.122085	-7.136527	0.819273
H	-4.825666	-5.810411	-1.110914
H	-4.803324	-5.512536	0.629622
H	-4.920120	-4.154142	-0.493496

$\{21TMS\}_2$

E= -2781.798149

C	-1.807591	0.532386	1.306046
C	-1.730860	-0.187516	2.444060
C	-1.165786	-1.497631	2.690937
C	-1.052775	-2.545260	1.851925
C	-1.472260	-2.654051	0.468287
C	-1.580417	-1.712479	-0.489343
P	-1.105739	0.056679	-0.316036
P	1.105651	-0.056727	-0.315994
C	1.580483	1.712370	-0.489419
C	1.472185	2.654049	0.468094
C	1.052424	2.545410	1.851652
C	1.165240	1.497855	2.690796
C	1.730383	0.187736	2.444144
C	1.807427	-0.532226	1.306181
H	0.889446	1.681365	3.726853
H	2.148609	-0.263421	3.345697
Si	2.819487	-2.129451	1.261498
Si	2.300221	2.146041	-2.180062
H	1.741058	3.675628	0.194095
H	0.702374	3.475727	2.293138
H	-0.890181	-1.681029	3.727065
H	-2.149240	0.263736	3.345494
Si	-2.819531	2.129704	1.261016
Si	-2.299922	-2.146372	-2.180037
H	-1.741035	-3.675670	0.194337

H	-0.702749	-3.475515	2.293559
C	-3.102735	2.750749	3.014246
C	-1.972889	3.476288	0.260635
C	-4.463350	1.716801	0.447947
C	-2.662693	-3.989594	-2.268010
C	-3.886596	-1.170958	-2.430151
C	-1.069285	-1.681254	-3.518484
C	3.102846	-2.750063	3.014864
C	1.972857	-3.476302	0.261448
C	4.463212	-1.716639	0.448183
C	2.663128	3.989234	-2.268115
C	3.886836	1.170483	-2.429986
C	1.069632	1.680929	-3.518560
H	-3.624708	3.712146	2.991040
H	-3.709823	2.061289	3.607635
H	-2.150976	2.900594	3.533276
H	-2.690378	4.272641	0.038438
H	-1.134118	3.920452	0.800254
H	-1.593985	3.086710	-0.687656
H	-5.113915	2.595690	0.402128
H	-4.309786	1.356996	-0.573390
H	-4.986517	0.932221	1.001870
H	-4.350102	-1.422352	-3.389150
H	-4.611343	-1.376962	-1.637585
H	-3.680033	-0.097006	-2.430293
H	-3.076656	-4.238737	-3.249781
H	-1.755218	-4.585065	-2.130852
H	-3.392264	-4.299683	-1.514337
H	-1.423625	-2.008212	-4.501095
H	-0.933880	-0.597332	-3.554612
H	-0.091570	-2.136693	-3.337348
H	2.690411	-4.272552	0.039099
H	1.134289	-3.920567	0.801297
H	1.593647	-3.086836	-0.686769
H	3.624876	-3.711431	2.991866
H	3.709903	-2.060405	3.608055
H	2.151120	-2.899836	3.533978
H	5.113724	-2.595561	0.402278
H	4.309513	-1.356829	-0.573132
H	4.986511	-0.932085	1.002021
H	1.424090	2.007781	-4.501165
H	0.934126	0.597019	-3.554625
H	0.091958	2.136501	-3.337535
H	3.077226	4.238306	-3.249849
H	1.755667	4.584763	-2.131117
H	3.392629	4.299326	-1.514377
H	4.350501	1.421885	-3.388906
H	4.611492	1.376369	-1.637305
H	3.680173	0.096550	-2.430209

2TTMS

E= -3452.194697

C	-5.714551	-0.390654	1.037949
Si	-3.974040	-0.800301	1.639027
C	-3.831924	-2.658957	1.894713
C	-3.648282	0.075172	3.277098
Si	-2.431730	-0.031818	0.042983
Si	-2.845041	-0.897409	-2.098244
C	-1.366839	-0.490337	-3.191066
C	-0.765244	-0.759109	0.480602
C	0.553188	-0.726925	0.473127
Si	2.254584	-0.055056	0.095968

Si	3.228399	-1.868851	-1.057083	H	4.021261	-4.129925	-0.395551
C	3.595620	-3.275624	0.141218	H	4.312500	-2.974565	0.910299
Si	-2.315964	2.309295	0.072202	H	2.684763	-3.613319	0.642412
C	-1.143618	2.793487	1.466477	H	5.286048	-2.236520	-2.392043
P	-0.083201	-2.308645	1.175358	H	4.674747	-0.601816	-2.667503
Si	2.531972	1.911168	-1.172927	H	5.565760	-0.977285	-1.184069
C	1.989793	1.634187	-2.956588	H	2.082510	4.331383	-1.042380
Si	3.341150	0.210684	2.167522	H	0.632007	3.447683	-0.564759
C	2.990692	1.923725	2.874155	H	1.970496	3.611811	0.569318
C	1.717540	3.462865	-0.483422	H	2.154545	2.535847	-3.555244
C	4.390444	2.251508	-1.139865	H	2.559197	0.819260	-3.413578
C	-1.745917	2.969458	-1.601448	H	0.931160	1.371624	-3.027069
C	-4.023984	3.035038	0.411064	H	4.628746	3.069289	-1.828085
C	-3.060048	-2.764635	-1.995387	H	4.708455	2.560701	-0.139492
C	-4.398907	-0.106443	-2.816686	H	4.988045	1.385004	-1.430147
C	2.762935	-1.100055	3.385476	H	-3.980935	4.129043	0.412337
C	5.201336	0.038504	1.899070	H	-4.742672	2.730928	-0.355928
C	2.008429	-2.469417	-2.361813	H	-4.415308	2.716167	1.381117
C	4.837794	-1.365928	-1.902167	H	-1.571706	4.049712	-1.564691
H	3.283707	-0.988708	4.342293	H	-0.826384	2.491334	-1.946257
H	1.688314	-1.020236	3.570664	H	-2.516687	2.783492	-2.356219
H	2.955646	-2.107559	3.007191	H	-0.945111	3.869578	1.480669
H	3.447031	2.030547	3.863622	H	-1.583000	2.516296	2.429654
H	3.402718	2.708234	2.232616	H	-0.187114	2.271477	1.377868
H	1.916727	2.104034	2.978462	H	-4.605924	-0.499617	-3.817131
H	5.738224	0.195898	2.840100	H	-5.275646	-0.301403	-2.192727
H	5.458624	-0.959109	1.532150	H	-4.284067	0.978260	-2.902539
H	5.575136	0.766561	1.174131	H	-1.434756	-1.004392	-4.154953
H	2.405115	-3.342088	-2.890587	H	-1.311266	0.584174	-3.385959
H	1.060003	-2.760090	-1.899848	H	-0.433751	-0.797106	-2.710248
H	1.799394	-1.692871	-3.103605	H	-3.136765	-3.204214	-2.995123

H	-2.210845	-3.228680	-1.484890
H	-3.966960	-3.028765	-1.443983
H	-6.457448	-0.686117	1.785757
H	-5.838687	0.678805	0.847013
H	-5.946231	-0.925678	0.112084
H	-4.601359	-3.003012	2.593915
H	-3.956959	-3.206641	0.957077
H	-2.855891	-2.926030	2.307570
H	-4.332013	-0.281663	4.053999
H	-2.625551	-0.110353	3.618505
H	-3.781196	1.157161	3.186125

$\{2TTMS\}_2$

E= -6904.459545

C	4.876713	-4.665902	1.314969
Si	4.738924	-3.005341	0.425113
C	6.392101	-2.135080	0.678513
Si	2.925697	-1.881916	1.415258
Si	1.219651	-3.466660	1.856752
C	1.231716	-4.953212	0.679464
C	2.243722	-0.381878	0.520124
C	2.348459	0.744210	-0.141635
Si	3.222909	1.937974	-1.303309
Si	4.886722	2.947886	0.008113
C	6.044677	1.587419	0.601687
P	0.826612	0.771286	0.936103
P	-0.791096	0.854181	-0.653215
C	-2.365004	0.695921	0.323949
Si	-3.378443	1.707315	1.542183
Si	-4.818451	0.172143	2.586147
C	-3.754696	-1.321320	3.025085
C	-2.214231	-0.352415	-0.446917
Si	-2.850611	-1.705026	-1.590035
Si	-1.003715	-2.304676	-2.934631
C	0.482082	-2.629981	-1.833192
Si	-3.842447	-3.703948	-0.814642
C	-5.214316	-3.557679	0.463029
Si	-4.334156	-0.609534	-3.062362
C	-3.667893	1.124823	-3.372372
Si	-2.022127	2.652836	3.231910
C	-0.707317	3.818809	2.562647
Si	-4.355574	3.454126	0.292754
C	-5.811092	2.869242	-0.749813
Si	3.522065	-1.054524	3.553650
C	1.972086	-0.530853	4.487382
Si	4.191876	0.808281	-3.122203
C	2.952025	-0.445216	-3.776405
Si	1.764950	3.597646	-2.139162
C	2.850918	4.984565	-2.824876
C	4.418222	-3.315831	-1.406038
C	4.463325	-2.393788	4.493940
C	4.641317	0.455983	3.498659
C	1.477889	-4.238898	3.561781
C	-0.439697	-2.576574	1.876897
C	4.576927	2.059119	-4.481984
C	5.810067	-0.044918	-2.667583
C	0.674672	2.954476	-3.534126
C	0.691712	4.343885	-0.787116
C	5.924062	4.176729	-0.977102
C	4.057247	3.820176	1.458625
C	-4.644017	-4.464833	-2.347450
C	-2.536595	-4.901504	-0.174471

C	-4.412401	-1.546126	-4.700492
C	-6.099560	-0.532448	-2.406274
C	-1.361699	-3.876005	-3.917758
C	-0.591145	-0.942651	-4.167563
C	-3.119741	3.666297	4.389716
C	-1.246678	1.273793	4.254265
C	-4.986384	4.802209	1.451850
C	-3.040064	4.202916	-0.828157
C	-5.574795	0.861807	4.170086
C	-6.209511	-0.344687	1.430413
H	0.262604	-1.250736	-4.778928
H	-1.424106	-0.729551	-4.841486
H	-0.323995	-0.015330	-3.654883
H	1.330866	-3.002650	-2.415739
H	0.801388	-1.728564	-1.303625
H	0.225198	-3.385420	-1.090117
H	-0.490886	-4.121392	-4.534910
H	-1.560984	-4.735490	-3.271993
H	-2.216661	-3.750954	-4.588047
H	-5.022943	-5.462618	-2.101968
H	-5.493998	-3.864397	-2.684571
H	-3.949420	-4.566987	-3.183452
H	-2.987687	-5.879004	0.024909
H	-1.733601	-5.044301	-0.902889
H	-2.086867	-4.541133	0.752487
H	-5.591747	-4.562765	0.681119
H	-4.882582	-3.112072	1.400760
H	-6.049674	-2.964945	0.082694
H	-4.356416	-2.127463	3.457638
H	-3.239651	-1.705133	2.140532
H	-2.984920	-1.049841	3.751973
H	-6.314558	0.157655	4.564920
H	-4.813915	1.009583	4.941341
H	-6.079128	1.818133	4.004945
H	-6.813285	-1.142181	1.874785
H	-6.870957	0.495473	1.202476
H	-5.804252	-0.720826	0.489266
H	-4.372352	1.722892	-3.959368
H	-3.479944	1.647105	-2.431746
H	-2.719237	1.086815	-3.914615
H	-6.745089	-0.027786	-3.133151
H	-6.502355	-1.537574	-2.247480
H	-6.178625	0.010497	-1.463520
H	-5.062430	-1.005686	-5.396797
H	-3.432904	-1.647042	-5.173484
H	-4.827625	-2.548863	-4.570423
H	-0.492852	1.675426	4.938943
H	-2.006308	0.766522	4.856741
H	-0.757526	0.530248	3.621373
H	-0.213126	4.327693	3.397506
H	0.056332	3.283697	1.995416
H	-1.143256	4.584114	1.913959
H	-2.527540	3.966048	5.261362
H	-3.487168	4.577737	3.913411
H	-3.984670	3.108493	4.754555
H	-3.487274	4.943119	-1.500051
H	-2.268689	4.706262	-0.238894
H	-2.536036	3.449288	-1.439080
H	-5.564362	5.536270	0.880753
H	-5.633475	4.407278	2.240082
H	-4.158349	5.332083	1.929458
H	-6.241225	3.721596	-1.286520

H	-5.510788	2.129307	-1.492638
H	-6.600304	2.430217	-0.133803
H	2.221060	-0.270236	5.521708
H	1.546293	0.355575	4.009520
H	1.192166	-1.295147	4.512559
H	0.212621	-5.295660	0.478694
H	1.713249	-4.755366	-0.279011
H	1.762432	-5.783067	1.154960
H	0.681966	-4.971390	3.735946
H	2.430236	-4.774788	3.610312
H	1.449926	-3.516196	4.379474
H	-1.222826	-3.226768	2.279127
H	-0.388235	-1.690754	2.516938
H	-0.745339	-2.235992	0.884536
H	4.714221	-2.028667	5.495527
H	3.894539	-3.317960	4.608741
H	5.403218	-2.635248	3.987879
H	4.759687	0.846323	4.515330
H	5.634803	0.218726	3.112409
H	4.218883	1.253464	2.883703
H	7.200763	-2.767114	0.296260
H	6.460687	-1.168268	0.178781
H	6.577506	-1.968154	1.744200
H	5.755473	-5.210924	0.954889
H	4.989966	-4.531265	2.394676
H	4.000751	-5.294925	1.142487
H	5.284970	-3.789503	-1.878196
H	3.558884	-3.980002	-1.534984
H	4.204512	-2.391183	-1.946098
H	6.144171	-0.687765	-3.488583
H	6.596617	0.691174	-2.477548
H	5.712766	-0.666705	-1.776518
H	3.396126	-1.088695	-4.542818
H	2.564140	-1.080440	-2.976374
H	2.098422	0.073154	-4.220311
H	5.043060	1.555137	-5.335077
H	3.674460	2.559402	-4.843703
H	5.270655	2.829721	-4.132540
H	2.235482	5.670105	-3.416839
H	3.300224	5.564588	-2.014150
H	3.657047	4.621268	-3.466866
H	0.172765	5.228050	-1.173017
H	-0.065942	3.635289	-0.448480
H	1.283844	4.647506	0.080547
H	0.116995	3.783141	-3.983282
H	1.257878	2.474800	-4.325637
H	-0.048293	2.231724	-3.148743
H	6.757928	4.532042	-0.362391
H	6.349887	3.703288	-1.867087
H	5.353139	5.048276	-1.302839
H	4.787509	4.122680	2.215679
H	3.536342	4.719220	1.115210
H	3.313857	3.176373	1.938125
H	6.629038	1.907174	1.469714
H	5.484108	0.695410	0.885554
H	6.741550	1.308100	-0.192951

8TTMS

E= -3529.628862

C	-5.432827	-2.471303	0.175376
Si	-3.563287	-2.215720	0.217886
C	-2.774500	-3.264810	-1.134337

C	-2.910684	-2.770061	1.898127	H	-3.135579	-2.971794	-2.124147
Si	-3.139906	0.069488	-0.125493	H	-1.685797	-3.161960	-1.129922
Si	-3.659868	1.252444	1.832031	H	-3.100328	-3.837168	2.053401
C	-2.248607	0.997989	3.055749	H	-1.832390	-2.605156	1.978056
Si	-4.463764	0.837770	-1.904238	H	-3.395206	-2.222862	2.712388
C	-3.831176	2.490924	-2.555416	H	-5.681474	-3.511588	0.408712
C	-1.306690	0.399507	-0.406813	H	-5.944742	-1.833063	0.901155
P	0.002250	-0.755099	-0.003275	H	-5.841499	-2.247521	-0.814419
C	1.303373	0.427750	-0.397487	H	-2.439564	1.517091	4.000315
Si	3.134429	0.082845	-0.125763	H	-2.096293	-0.062591	3.275671
Si	3.567704	0.697343	2.097331	H	-1.314549	1.386466	2.638282
C	3.666093	2.577231	2.225655	H	-5.546512	1.233489	3.467345
C	-4.476843	-0.407139	-3.320831	H	-6.105128	0.731559	1.865039
C	-6.225069	1.069807	-1.267174	H	-5.209378	-0.400808	2.884864
C	-0.740607	1.579167	-0.796937	H	-3.982738	3.671092	2.361563
C	0.721034	1.594098	-0.791494	H	-2.877728	3.470569	0.996638
Si	3.591737	-2.209035	-0.381187	H	-4.621399	3.300396	0.754086
C	2.946138	-3.188668	1.095429	H	-6.894705	1.374654	-2.077802
Si	4.520891	1.300147	-1.578802	H	-6.620342	0.145928	-0.835454
C	4.610404	0.460954	-3.265467	H	-6.265447	1.842601	-0.493627
C	2.799131	-2.867466	-1.958993	H	-4.504066	2.879609	-3.326748
C	5.462777	-2.438663	-0.480010	H	-3.768960	3.237448	-1.758062
C	-3.801202	3.095244	1.448329	H	-2.838219	2.385269	-3.001508
C	-5.282256	0.647759	2.581103	H	-5.046633	-0.021236	-4.172334
C	3.934260	3.077653	-1.816962	H	-3.461078	-0.621344	-3.665599
C	6.248838	1.341242	-0.820908	H	-4.932867	-1.353469	-3.015728
C	2.153534	0.082570	3.181557	H	3.776919	2.896585	3.266864
C	5.191198	-0.058105	2.692077	H	4.517216	2.971748	1.662664
H	-1.311684	2.459278	-1.080142	H	2.757587	3.039691	1.827736
H	1.275008	2.485413	-1.069613	H	2.336472	0.314848	4.235680
H	-3.011654	-4.323554	-0.986879	H	1.209035	0.553677	2.893134

H	2.023663	-0.999826	3.091965
H	5.411614	0.252249	3.718433
H	5.143697	-1.151114	2.678395
H	6.030516	0.247502	2.060728
H	5.715156	-3.502800	-0.528798
H	5.881098	-1.957271	-1.368791
H	5.961911	-2.015537	0.396682
H	6.953242	1.840172	-1.494268
H	6.251044	1.887182	0.127200
H	6.627319	0.333800	-0.626537
H	5.231879	1.040613	-3.955668
H	5.044723	-0.539750	-3.184771
H	3.616899	0.358412	-3.711471
H	3.015600	-3.932993	-2.087288
H	1.712129	-2.747403	-1.931515
H	3.171600	-2.338045	-2.840547
H	4.691639	3.653530	-2.358908
H	3.010515	3.117758	-2.400750
H	3.755682	3.575475	-0.859590
H	3.142810	-4.257633	0.963259
H	3.430829	-2.871450	2.023582
H	1.867415	-3.057682	1.218027

{8TTMS}$_2$

E= -7059.336092

Si	1.258202	5.402891	-1.090790
Si	0.026405	3.430122	-1.538029
Si	0.118468	3.213750	-3.906415
Si	-2.207895	3.755280	-0.904183
C	0.879118	1.822640	-0.988265
P	0.218034	0.124046	-1.134725
P	-0.457110	-0.755371	0.788843
C	-2.212625	-0.260631	0.914097
C	-2.291915	0.606321	1.955049
C	-1.049962	0.938733	2.616611
C	0.076884	0.341344	2.145574
Si	1.635706	0.448152	3.237634
Si	2.265848	2.705417	3.433305
C	2.223156	1.715485	-1.171110
C	2.759700	0.384762	-1.344443
C	1.862656	-0.632601	-1.304646
Si	2.244183	-2.396147	-1.890291
Si	2.311270	-4.179430	-0.334184
Si	0.674774	-2.861077	-3.597914
Si	4.350168	-2.378466	-2.960328
Si	0.874555	-0.255037	5.369343
Si	3.622728	-0.795356	2.946338
Si	-3.751076	-1.218768	0.324747
Si	-5.612314	-0.371318	1.523686
Si	-4.371643	-1.255944	-1.968789
Si	-3.492730	-3.401369	1.220074
H	-1.057030	1.586400	3.486709
H	-3.226034	0.999844	2.339398
H	3.814003	0.245525	-1.552896
H	2.866977	2.578453	-1.312022
C	0.326884	6.868259	-1.838933
C	1.469702	5.853783	0.721545
C	2.975454	5.313530	-1.875859
C	-2.883334	5.215970	-1.891442
C	-3.258650	2.267200	-1.355510
C	-2.374900	4.117350	0.936594
C	0.180301	4.869411	-4.813366

C	1.677051	2.252411	-4.360803
C	-1.368224	2.273705	-4.576589
C	4.781130	-4.129480	-3.521417
C	4.255419	-1.258293	-4.476061
C	1.301414	-4.207899	-4.762421
C	-0.945987	-3.467473	-2.854061
C	0.363246	-1.317811	-4.631779
C	1.985372	-5.755354	-1.323309
C	4.036724	-4.365484	0.411917
C	1.058099	-4.092612	1.065922
C	4.880697	0.019195	4.100406
C	3.452559	-2.581546	3.529521
C	4.412474	-0.762085	1.244193
C	2.274576	-0.409229	6.628111
C	-0.373457	0.959134	6.108155
C	0.025174	-1.924464	5.189904
C	3.058510	3.034342	5.114487
C	3.511403	3.139312	2.082547
C	0.734584	3.797470	3.327229
C	-4.930684	-3.019155	-2.346956
C	-5.842609	-0.118277	-2.303083
C	-3.032691	-0.758737	-3.203147
C	-5.164163	-4.175464	1.639658
C	-2.621293	-4.627860	0.088284
C	-2.489749	-3.232698	2.804084
C	-7.186921	-1.307241	1.054902
C	-5.396125	-0.603642	3.388550
C	-5.907723	1.462889	1.179902
C	5.777787	-1.811624	-1.862274
H	6.720285	-1.941386	-2.405153
H	5.708358	-0.762424	-1.566724

H	5.833011	-2.406197	-0.947332
H	5.728685	-4.115048	-4.070295
H	4.906292	-4.804564	-2.670269
H	4.021111	-4.557391	-4.178425
H	5.241238	-1.138981	-4.936840
H	3.584332	-1.680888	-5.229737
H	3.877599	-0.264087	-4.219472
H	2.063384	-6.628604	-0.667276
H	0.986737	-5.761511	-1.767850
H	2.713852	-5.878810	-2.129856
H	4.037233	-5.196809	1.125051
H	4.780822	-4.587906	-0.357861
H	4.363185	-3.472239	0.946027
H	1.252765	-4.897945	1.782790
H	1.090973	-3.139346	1.595375
H	0.039800	-4.212200	0.693966
H	4.423531	-3.082377	3.451951
H	3.141226	-2.625967	4.576449
H	2.736283	-3.158377	2.943706
H	5.398583	-1.235954	1.294778
H	3.818227	-1.284094	0.495520
H	4.548837	0.265076	0.895685
H	5.779399	-0.606395	4.132254
H	5.182047	1.006512	3.740822
H	4.515999	0.128587	5.123060
H	3.875208	4.167293	2.176481
H	4.379339	2.475908	2.135873
H	3.062239	3.015175	1.093407
H	3.319125	4.094955	5.195957
H	2.367031	2.802385	5.929712
H	3.969954	2.453404	5.269140

H	1.000589	4.851735	3.444539
H	0.220568	3.680175	2.370492
H	0.028155	3.544298	4.123447
H	1.852360	-0.708855	7.593204
H	3.014647	-1.161418	6.345105
H	2.796580	0.540033	6.775977
H	-0.561547	0.697718	7.154829
H	-0.009650	1.990540	6.086318
H	-1.332829	0.924660	5.586098
H	-0.332260	-2.288049	6.158923
H	-0.836805	-1.841800	4.524324
H	0.694998	-2.680301	4.770721
H	-1.708052	-3.590535	-3.630107
H	-0.809673	-4.436834	-2.368283
H	-1.336981	-2.767474	-2.111024
H	-0.366395	-1.534356	-5.419177
H	-0.035745	-0.504001	-4.024165
H	1.281472	-0.962891	-5.107486
H	0.512194	-4.467984	-5.475736
H	2.166019	-3.868090	-5.339312
H	1.582938	-5.120225	-4.230339
H	3.436208	6.306907	-1.873619
H	3.638341	4.648257	-1.314737
H	2.941777	4.963866	-2.911516
H	1.019574	7.709896	-1.946600
H	-0.117837	6.673319	-2.814576
H	-0.469723	7.189204	-1.161831
H	1.984615	6.818454	0.789670
H	0.502568	5.955689	1.220505
H	2.054732	5.119404	1.271294
H	-3.425172	4.275271	1.203792
H	-1.993719	3.284101	1.531780
H	-1.816897	5.014473	1.220313
H	-3.958560	5.315969	-1.709425
H	-2.410125	6.163050	-1.626059
H	-2.743615	5.062170	-2.966035
H	-4.268725	2.396955	-0.956261
H	-3.329616	2.151611	-2.438993
H	-2.848865	1.346420	-0.938704
H	0.261385	4.682760	-5.889533
H	-0.726278	5.459043	-4.649350
H	1.038680	5.478037	-4.516517
H	1.741149	2.139255	-5.448099
H	2.583121	2.762592	-4.020636
H	1.674198	1.253738	-3.917865
H	-1.240336	2.082042	-5.647030
H	-1.487034	1.310907	-4.077456
H	-2.295244	2.839652	-4.444778
H	-6.120698	-0.217957	-3.358197
H	-6.724965	-0.355695	-1.705980
H	-5.583120	0.927650	-2.127600
H	-3.356038	0.134215	-3.743247
H	-2.074459	-0.534383	-2.731527
H	-2.867723	-1.550251	-3.938232
H	-5.417686	-3.055659	-3.327014
H	-4.075915	-3.699877	-2.371588
H	-5.642756	-3.395428	-1.606128
H	-5.007877	-5.219701	1.930420
H	-5.659589	-3.671341	2.472881
H	-5.847112	-4.168343	0.785644
H	-8.060681	-0.721328	1.359291
H	-7.275527	-1.497407	-0.016619

H	-7.241057	-2.270662	1.565646
H	-6.296726	-0.258775	3.907733
H	-5.253161	-1.658197	3.639591
H	-4.545313	-0.052659	3.795845
H	-2.478123	-4.175846	3.359969
H	-1.454019	-2.968811	2.573569
H	-2.897119	-2.456894	3.458427
H	-6.684899	1.845674	1.849845
H	-5.009389	2.068958	1.324339
H	-6.249652	1.613314	0.152168
H	-2.330062	-5.514032	0.662419
H	-3.274715	-4.953021	-0.724755
H	-1.722715	-4.202415	-0.356332

12TTMS

E= -3568.935621

C	4.483021	0.533565	-2.957999
Si	2.790212	0.289493	-2.158381
C	1.713707	1.792305	-2.524106
Si	3.032012	0.062652	0.176833
Si	4.713881	1.534877	0.915158
C	4.836934	1.658997	2.796723
C	1.346218	0.439400	0.975032
C	1.192825	1.320944	1.986706
C	-0.091220	1.671538	2.672816
C	-1.360233	1.262069	1.991186
C	-1.471541	0.387990	0.968672
Si	-3.119272	-0.006636	0.098015
Si	-4.934766	0.954142	1.247886
C	-6.560325	0.476789	0.419189
P	-0.045827	-0.431049	0.247265
Si	-2.917412	1.064999	-1.992763
C	-1.827617	0.109282	-3.196161
Si	-3.318022	-2.357238	0.022677
C	-2.609024	-3.043122	1.629194
C	-4.610178	1.326790	-2.786766
C	-2.129212	2.745558	-1.656661
Si	3.689252	-2.143305	0.651760
C	2.247285	-3.343810	0.487537
C	5.041058	-2.637252	-0.571020
C	4.384646	-2.254945	2.402585
C	2.003208	-1.244433	-2.924681
C	6.375486	0.882981	0.302820
C	4.417796	3.256564	0.203674
C	-5.128918	-2.874317	-0.102010
C	-2.388747	-3.103193	-1.437215
C	-4.980274	0.347845	3.034682
C	-4.819659	2.838964	1.216746
H	-0.076690	1.230583	3.684126
H	2.065399	1.830461	2.389645
H	-2.254597	1.729771	2.399133
H	-0.116941	2.754100	2.856048
H	1.593452	1.934372	-3.602934
H	0.717536	1.671084	-2.090821
H	2.149930	2.704624	-2.106749
H	1.828580	-1.088309	-3.994328
H	2.650491	-2.119558	-2.815936
H	1.044185	-1.478258	-2.455831
H	4.379385	0.602907	-4.045686
H	4.965807	1.451835	-2.611539
H	5.153495	-0.302788	-2.738728
H	5.435245	-3.630104	-0.331562

H	4.654119	-2.671675	-1.593542
H	5.876838	-1.932176	-0.555163
H	2.590272	-4.372115	0.643244
H	1.468012	-3.130997	1.224579
H	1.786849	-3.283114	-0.502248
H	4.618972	-3.292956	2.660188
H	5.304193	-1.670765	2.504163
H	3.665730	-1.881149	3.137651
H	5.171497	3.965376	0.561524
H	4.463293	3.242745	-0.889352
H	3.432951	3.638588	0.488433
H	5.773669	2.157382	3.068169
H	4.020667	2.237579	3.236595
H	4.836177	0.670943	3.264706
H	7.169806	1.597779	0.541547
H	6.626154	-0.064544	0.789169
H	6.384066	0.718742	-0.777290
H	-7.388926	1.014931	0.891540
H	-6.563822	0.726414	-0.644877
H	-6.762430	-0.592630	0.510882
H	-5.642181	3.279268	1.790101
H	-3.880997	3.213465	1.633155
H	-4.895985	3.211188	0.190540
H	-5.803312	0.816948	3.583618
H	-5.131579	-0.735483	3.069679
H	-4.050337	0.567181	3.566179
H	-4.501321	1.794581	-3.770528
H	-5.139989	0.379487	-2.924414
H	-5.241323	1.979506	-2.176585
H	-1.933300	3.279660	-2.591983
H	-2.778753	3.370959	-1.037509
H	-1.179401	2.633331	-1.126133
H	-1.644561	0.705226	-4.096516
H	-0.860196	-0.130711	-2.748550
H	-2.294368	-0.830597	-3.501669
H	-5.199507	-3.957916	-0.242327
H	-5.678312	-2.621906	0.809464
H	-5.635298	-2.394881	-0.944651
H	-2.356069	-4.193831	-1.343889
H	-2.879623	-2.863512	-2.384620
H	-1.360591	-2.735348	-1.486053
H	-2.756349	-4.126576	1.686256
H	-1.536135	-2.843251	1.700002
H	-3.090267	-2.588959	2.500445

{12TTMS}$_2$

E= -7137.940779

Si	-3.979664	-3.497490	-2.029364
Si	-2.312766	-1.836386	-2.367571
Si	-3.476297	-0.202756	-3.653897
Si	-0.559245	-2.846671	-3.650022
C	-1.800687	-1.192855	-0.630268
P	-0.988878	0.460581	-0.356162
P	1.144222	-0.304765	-0.071917
C	1.266311	-0.833009	1.696330
Si	1.611001	-2.639262	2.248174
Si	0.400928	-4.565417	1.560751
C	-2.148661	-1.925001	0.435966
C	-1.969923	-1.557322	1.880066
C	-2.007433	-0.091970	2.160276
C	-1.663257	0.887764	1.312994
Si	-2.314744	2.613492	1.833596

Si	-1.754015	3.191504	4.063632
Si	-1.843593	4.537486	0.574783
Si	-4.636495	2.260994	1.694322
C	2.072716	1.309307	-0.023746
C	2.110394	2.001001	1.120275
C	1.433532	1.595660	2.395800
C	1.378789	0.116989	2.635120
Si	3.064620	1.943051	-1.529164
Si	4.797747	0.483793	-2.216025
Si	1.689081	2.457305	-3.375891
Si	4.271298	3.924786	-1.016347
Si	3.849269	-3.071631	1.647146
Si	1.420251	-2.701897	4.614298
H	-1.026045	-1.975409	2.249626
H	-2.432922	0.163470	3.128634
H	-2.629328	-2.888552	0.288900
H	-2.745983	-2.050474	2.474887
H	1.922565	2.080032	3.245441
H	2.656050	2.939330	1.175323
H	1.488746	-0.162186	3.677939
H	0.408038	1.988734	2.376285
C	2.662972	3.459839	-4.644518
C	1.021747	0.905951	-4.210463
C	0.221053	3.495227	-2.834572
C	5.547782	4.222904	-2.378250
C	3.202800	5.475662	-0.946711
C	5.216912	3.802868	0.617215
C	5.323039	0.917009	-3.977565
C	6.305800	0.670415	-1.092330
C	4.296953	-1.326002	-2.220496
C	-0.824404	-4.712438	-3.792939

C	-0.488573	-2.222429	-5.429359
C	1.155108	-2.582347	-2.933003
C	-4.739230	-3.912722	-3.708797
C	-3.379548	-5.123162	-1.272944
C	-5.346129	-2.869584	-0.885027
C	-3.493092	-0.672110	-5.482626
C	-5.296235	-0.083089	-3.164949
C	-2.734367	1.514808	-3.489287
C	1.509404	-6.026523	2.025263
C	-1.228590	-4.859535	2.474235
C	0.076951	-4.722747	-0.280526
C	2.742683	-1.685203	5.508824
C	-0.287833	-2.065889	5.112720
C	4.700760	-4.137215	2.952721
C	3.986657	-3.987324	-0.000322
C	4.722794	-1.406512	1.549072
C	-2.554736	5.997175	1.546165
C	0.007986	4.801164	0.404288
C	-3.227033	4.098859	4.818271
C	-1.340925	1.687509	5.122991
C	-0.270179	4.357383	4.186752
C	-5.641973	3.852599	1.807237
C	-4.949638	1.439376	0.030118
C	-5.243766	1.074096	3.033323
H	-0.423934	3.726319	-3.688551
H	-0.373796	2.941259	-2.105053
H	0.534316	4.437231	-2.379215
H	0.432617	1.177162	-5.093571
H	1.816823	0.228066	-4.533217
H	0.367624	0.357685	-3.526127
H	2.032683	3.641420	-5.521599

H	2.947513	4.434786	-4.238470
H	3.570138	2.954939	-4.983569
H	6.175270	0.292997	-4.267405
H	4.514213	0.719207	-4.687344
H	5.620743	1.961918	-4.087144
H	5.183499	-1.966346	-2.174960
H	3.651033	-1.574049	-1.378326
H	3.749736	-1.575116	-3.131816
H	7.059858	-0.072495	-1.374111
H	6.767788	1.657093	-1.173311
H	6.052939	0.503585	-0.042824
H	3.844301	6.356256	-0.834074
H	2.615402	5.605653	-1.860328
H	2.509280	5.454724	-0.104664
H	5.996927	4.571261	0.641483
H	4.570571	3.968291	1.483168
H	5.699127	2.830843	0.741752
H	6.040264	5.183597	-2.193423
H	6.323144	3.454589	-2.403372
H	5.090166	4.270986	-3.368903
H	0.376684	-2.690148	-5.912523
H	-1.376901	-2.497255	-6.001901
H	-0.356367	-1.140432	-5.491533
H	1.864295	-3.209172	-3.485797
H	1.469954	-1.544423	-3.031319
H	1.226688	-2.827949	-1.875235
H	-0.082937	-5.109976	-4.494678
H	-0.681192	-5.217420	-2.836432
H	-1.814037	-4.973465	-4.176483
H	-3.317245	2.233970	-4.074762
H	-2.737153	1.834381	-2.445817

H	-1.699999	1.546921	-3.836441
H	-4.270886	-0.086761	-5.984655
H	-2.542180	-0.454283	-5.970870
H	-3.719648	-1.729796	-5.642463
H	-5.746365	0.749791	-3.716498
H	-5.849359	-0.988480	-3.427992
H	-5.439217	0.108459	-2.102051
H	-5.493841	-4.697949	-3.596033
H	-5.226792	-3.045706	-4.161169
H	-3.987456	-4.274762	-4.415888
H	-4.243668	-5.783049	-1.137982
H	-2.664690	-5.636964	-1.917584
H	-2.910536	-4.992912	-0.294091
H	-6.106452	-3.648950	-0.764874
H	-4.959099	-2.627148	0.108363
H	-5.838877	-1.977559	-1.273776
C	1.591684	-4.458782	5.279735
H	1.512604	-4.429756	6.371709
H	0.809136	-5.125899	4.910621
H	2.561293	-4.897738	5.029733
H	-0.366768	-1.974542	6.200770
H	-0.502386	-1.086673	4.679116
H	-1.070969	-2.751436	4.776418
H	2.680522	-1.882057	6.584470
H	3.748431	-1.961074	5.179952
H	2.635548	-0.607335	5.367400
H	0.976488	-6.954915	1.792832
H	2.439289	-6.031720	1.453346
H	1.766353	-6.050368	3.085445
H	-1.535457	-5.897218	2.305073
H	-1.127381	-4.717444	3.553219

H	-2.039648	-4.218453	2.126581
H	-0.465158	-5.655458	-0.467442
H	-0.521929	-3.897587	-0.668992
H	1.013816	-4.762353	-0.841061
H	5.738693	-4.331435	2.662708
H	4.716016	-3.650052	3.931360
H	4.201098	-5.103408	3.065719
H	4.988716	-3.859564	-0.421520
H	3.820642	-5.059039	0.135677
H	3.271347	-3.629592	-0.743871
H	5.758746	-1.524489	1.216416
H	4.213441	-0.731270	0.856565
H	4.729132	-0.917861	2.528346
H	-0.063434	4.561253	5.242938
H	0.634353	3.930900	3.748700
H	-0.455304	5.315949	3.696913
H	-1.048054	2.010837	6.127248
H	-2.183324	0.998697	5.228886
H	-0.503822	1.129929	4.694257
H	-3.001648	4.400283	5.846425
H	-3.465497	5.002687	4.250426
H	-4.123677	3.473847	4.841946
H	-6.326174	0.935609	2.942103
H	-4.777998	0.090731	2.925787
H	-5.038677	1.438230	4.043866
H	-6.015414	1.228591	-0.106566
H	-4.621692	2.068620	-0.802324
H	-4.405360	0.491306	-0.032372
H	-6.711320	3.627683	1.739079
H	-5.470071	4.374775	2.752470
H	-5.396841	4.539439	0.992413
C	-2.710508	4.568116	-1.095843
H	-2.513576	5.522121	-1.596450
H	-2.381539	3.771963	-1.762568
H	-3.793587	4.477683	-0.966976
H	0.215047	5.685487	-0.205872
H	0.460496	4.955966	1.388595
H	0.499790	3.944272	-0.059978
H	-2.409771	6.916651	0.968982
H	-3.627542	5.883433	1.720837
H	-2.071136	6.138749	2.515025

21TTMS

E= -3607.012511

C	-4.477303	-2.993082	-0.394736
Si	-2.999829	-2.293958	0.553212
C	-3.237521	-2.560167	2.404197
Si	-2.940914	0.020177	0.093664
Si	-3.137262	0.376283	-2.219422
C	-1.987831	-0.759095	-3.187768
Si	-4.817995	0.988394	1.158193
C	-5.331694	2.623537	0.358604
C	-6.314015	-0.156369	1.056857
C	-4.404876	1.295431	2.970846
C	-1.393717	0.980458	0.705196
P	0.034190	-0.033415	1.048701
C	1.508717	1.007999	0.870924
Si	3.010761	0.059213	0.160685
Si	3.563495	-1.946791	1.267660
C	2.090420	-3.048600	1.659009
C	-1.502673	-3.274298	-0.023135
C	-1.549541	2.331513	0.848268

C	-0.667284	3.321938	1.411657
C	0.673277	3.313234	1.566991
C	1.643030	2.332195	1.132616
Si	2.432055	-0.422487	-2.070146
C	1.422378	1.032148	-2.718169
Si	4.942014	1.419004	0.146365
C	5.316932	2.149301	1.846699
C	3.981102	-0.646015	-3.124998
C	1.412487	-2.002618	-2.170462
C	-4.918514	0.032645	-2.743831
C	-2.706657	2.170504	-2.600212
C	6.433175	0.378652	-0.356747
C	4.753556	2.822015	-1.103415
C	4.695549	-2.912683	0.103720
C	4.500183	-1.570651	2.861413
H	-1.172344	4.234637	1.720218
H	-2.519153	2.745692	0.578641
H	2.635041	2.764343	1.020585
H	1.109539	4.215425	1.988710
H	4.734794	-2.496260	3.397014
H	5.443896	-1.056178	2.657424
H	3.909407	-0.936568	3.528693
H	2.437434	-3.965053	2.148326
H	1.368711	-2.558645	2.316544
H	1.567626	-3.332878	0.742041
H	5.029900	-3.838239	0.583711
H	4.167149	-3.188018	-0.813758
H	5.583846	-2.344208	-0.181495
H	1.072353	0.855415	-3.740251
H	0.547708	1.207890	-2.084189
H	2.016453	1.951026	-2.716189

H	1.052208	-2.176738	-3.189608
H	2.000804	-2.873953	-1.867810
H	0.545480	-1.943939	-1.508653
H	3.707935	-0.881269	-4.158778
H	4.597461	0.257182	-3.141456
H	4.598635	-1.466978	-2.748194
H	5.650541	3.449987	-1.114303
H	4.609145	2.430047	-2.114346
H	3.898030	3.463703	-0.875355
H	6.272106	2.684273	1.818439
H	4.552118	2.853102	2.184081
H	5.395867	1.363273	2.602578
H	7.310583	1.023857	-0.470317
H	6.671093	-0.370696	0.403341
H	6.273787	-0.139662	-1.305638
H	-5.249971	1.760267	3.488867
H	-4.168688	0.357926	3.482345
H	-3.538202	1.954053	3.074047
H	-6.253376	2.987732	0.824532
H	-4.577267	3.406640	0.472198
H	-5.525681	2.502150	-0.710757
H	-7.179093	0.324113	1.525503
H	-6.578797	-0.379816	0.019313
H	-6.140061	-1.104067	1.573277
H	-2.823148	2.379872	-3.668340
H	-3.353648	2.858692	-2.048425
H	-1.672061	2.390777	-2.322339
H	-5.041771	0.199425	-3.818847
H	-5.203269	-1.001547	-2.531429
H	-5.625182	0.685253	-2.223256
H	-2.084977	-0.580511	-4.263753

H	-0.944656	-0.589147	-2.912215
H	-2.214711	-1.812815	-3.000515
H	-3.305241	-3.627846	2.636573
H	-2.393499	-2.146375	2.963846
H	-4.149854	-2.078889	2.768621
H	-4.621315	-4.045533	-0.128597
H	-5.411729	-2.465623	-0.195129
H	-4.291166	-2.951900	-1.472490
H	-1.673343	-4.340456	0.161458
H	-1.329990	-3.144367	-1.094907
H	-0.597956	-2.972974	0.505800

$\{21TTMS\}_2$

E= -7214.057645

Si	5.305077	-2.142348	-0.445717
Si	3.067312	-2.187734	-1.277027
Si	3.333934	-0.950158	-3.288194
C	2.056634	-1.809532	0.335326
C	2.470100	-2.664136	1.303642
C	2.305956	-2.589576	2.742812
C	2.397689	-1.469970	3.490973
C	2.512318	-0.097509	3.044830
C	1.942978	0.525054	1.973783
Si	2.300043	2.407025	1.911877
Si	0.556456	3.509181	3.049725
Si	2.689402	-4.456459	-1.902786
P	0.823014	-0.640070	1.085650
P	-1.025669	0.184842	0.135885
C	-1.975285	-1.384120	0.435258
Si	-3.208078	-1.672566	1.883829
Si	-1.930667	-2.776596	3.536268
Si	4.209190	2.987620	3.198356
Si	2.637920	3.303429	-0.222704
C	-1.225929	0.291594	-1.699437
C	-0.801653	-0.679452	-2.537410
C	-0.304849	-1.996301	-2.255445
C	-0.708626	-2.803426	-1.252766
C	-1.667602	-2.532147	-0.216415
Si	-2.293095	1.616999	-2.598404
Si	-4.266386	0.426781	-3.123267
Si	-2.982867	3.571656	-1.478921
Si	-1.402298	2.302287	-4.692582
Si	-4.286996	0.117418	2.975348
Si	-4.874273	-3.231636	1.243331
H	-0.361823	-3.832447	-1.282669
H	-2.167373	-3.439111	0.119823
H	-0.906180	-0.499412	-3.606005
H	0.318530	-2.440383	-3.022812
H	2.308824	-3.541747	3.267166
H	3.106037	-3.498064	1.015426
H	3.115494	0.508695	3.716506
H	2.474839	-1.603603	4.567665
C	-3.044545	-3.754730	4.710781
C	-0.779255	-4.015034	2.703479
C	-0.945034	-1.532020	4.549266
C	-4.908763	-0.524811	4.640877
C	-3.092589	1.517199	3.366523
C	-5.797285	0.745350	2.039772
C	-6.197690	-3.301009	2.587831
C	-5.717858	-2.777946	-0.376811
C	-4.198391	-4.989514	1.059902
C	-5.189007	1.156621	-4.599537

C	-3.858421	-1.371637	-3.513734
C	-5.412050	0.482420	-1.631321
C	-4.408308	4.339288	-2.454755
C	-3.709557	3.231217	0.226097
C	-1.570569	4.819626	-1.440075
C	-2.556178	3.607789	-5.423009
C	0.295480	3.121272	-4.652203
C	-1.335703	0.867042	-5.920960
C	4.247109	-2.138757	-4.441808
C	1.845358	-0.353470	-4.267801
C	4.427061	0.563686	-3.051450
C	4.335311	-5.234690	-2.406259
C	2.009198	-5.470963	-0.461177
C	1.534438	-4.720041	-3.380704
C	6.647558	-2.351209	-1.760169
C	5.559925	-0.479751	0.394366
C	5.649117	-3.473792	0.858670
C	4.620589	4.800335	2.856203
C	3.914753	2.835295	5.061600
C	5.738301	1.968552	2.790582
C	1.103687	5.194285	3.706901
C	-0.911101	3.860737	1.933184
C	0.082445	2.415063	4.507617
C	2.128277	5.118909	-0.217308
C	4.463986	3.258227	-0.699601
C	1.622260	2.324517	-1.465516
H	-3.611340	4.107815	0.872290
H	-4.775535	3.015274	0.118867
H	-3.242938	2.387491	0.733420
H	-1.837491	5.706005	-0.855755
H	-0.662904	4.392577	-1.011065

H	-1.334144	5.146444	-2.457823
H	-4.756201	5.231804	-1.923058
H	-4.140301	4.637943	-3.468489
H	-5.254106	3.648376	-2.517252
H	0.468698	3.608354	-5.618041
H	0.359203	3.885617	-3.873300
H	1.099893	2.402090	-4.493945
H	-2.279562	3.770258	-6.470345
H	-3.610160	3.326402	-5.395762
H	-2.443267	4.563866	-4.904334
H	-1.131795	1.255605	-6.924440
H	-0.543959	0.153042	-5.681067
H	-2.284061	0.323639	-5.962179
H	-6.112873	0.594181	-4.770343
H	-5.463237	2.201714	-4.432878
H	-4.595823	1.107569	-5.516820
H	-4.779743	-1.928822	-3.713387
H	-3.215420	-1.458597	-4.394660
H	-3.343056	-1.851872	-2.677351
H	-6.322908	-0.095901	-1.811874
H	-4.917337	0.062667	-0.751198
H	-5.706632	1.509059	-1.397812
H	4.599200	3.541534	-1.748410
H	5.039411	3.960492	-0.091185
H	4.902941	2.268988	-0.560375
H	1.876315	2.603402	-2.489936
H	1.786276	1.248998	-1.351303
H	0.551938	2.499269	-1.326131
H	2.245791	5.558320	-1.213253
H	1.089467	5.257018	0.088770
H	2.758415	5.687391	0.473644

H	-0.760567	2.842399	5.059334
H	-0.200959	1.413453	4.176571
H	0.923513	2.308195	5.198931
H	-1.808602	4.064806	2.523630
H	-0.711791	4.741565	1.317512
H	-1.129025	3.023304	1.267977
H	0.245200	5.681762	4.181660
H	1.901745	5.132848	4.449864
H	1.441870	5.843985	2.894153
H	4.800508	3.195912	5.595535
H	3.062324	3.432796	5.393558
H	3.738854	1.804118	5.379976
H	6.583791	2.322587	3.390094
H	5.591189	0.908016	3.007758
H	6.009563	2.055183	1.735816
H	5.489119	5.091571	3.456638
H	4.874162	4.973723	1.807903
H	3.797977	5.469286	3.116594
H	-3.616681	2.474595	3.446777
H	-2.599799	1.320654	4.321812
H	-2.312580	1.619558	2.612239
H	-0.304984	-2.039848	5.278369
H	-0.304389	-0.937329	3.893745
H	-1.606789	-0.854534	5.097336
H	-5.443125	0.285308	5.149510
H	-5.594547	-1.369654	4.550863
H	-4.079201	-0.824920	5.286660
H	-6.309796	1.506516	2.637770
H	-5.536145	1.192613	1.081462
H	-6.508944	-0.063094	1.847425
H	-2.433210	-4.209594	5.497730
H	-3.796081	-3.128056	5.196635
H	-3.566070	-4.564793	4.193108
H	-0.137000	-4.496801	3.447898
H	-1.344927	-4.799990	2.192671
H	-0.138259	-3.519278	1.971599
H	-5.035469	-5.680664	0.913368
H	-3.534963	-5.098760	0.197634
H	-3.652563	-5.315686	1.948841
H	-6.387497	-3.586812	-0.687439
H	-6.314427	-1.869576	-0.267250
H	-4.995980	-2.607787	-1.179256
H	-6.952687	-4.047067	2.318952
H	-5.778687	-3.583162	3.557121
H	-6.706764	-2.340812	2.706262
H	6.480607	-0.480191	0.986146
H	5.637476	0.326785	-0.337972
H	4.727063	-0.248346	1.063836
H	6.732113	-3.555637	0.999160
H	5.212047	-3.207147	1.824194
H	5.278853	-4.464760	0.580349
H	7.624073	-2.282146	-1.268756
H	6.594879	-3.325957	-2.251898
H	6.615649	-1.579785	-2.532612
H	1.651393	-6.436767	-0.833404
H	2.783818	-5.674455	0.284113
H	1.181829	-4.974788	0.052948
H	4.181004	-6.297984	-2.618468
H	4.744406	-4.772007	-3.307561
H	5.087468	-5.159400	-1.617511
H	1.716638	-5.720989	-3.786024
H	0.476117	-4.668026	-3.115622

H	1.711973	-4.005442	-4.188838
H	4.601440	1.033488	-4.025496
H	3.944496	1.300757	-2.411020
H	5.400299	0.328051	-2.617064
H	4.529278	-1.590743	-5.347388
H	5.159168	-2.545608	-4.003972
H	3.616769	-2.976975	-4.749239
H	2.215846	0.179235	-5.150434
H	1.217096	-1.174973	-4.620298
H	1.228517	0.333945	-3.689322

2DMP

E= -1037.988115

C	0.217631	-0.472761	0.267958
C	0.019709	0.343935	1.396947
C	1.031363	1.219527	1.838468
C	2.233021	1.257105	1.136327
C	2.439970	0.446183	0.031139
C	1.437738	-0.412826	-0.395888
C	-1.244786	0.289639	2.110956
P	-3.059109	0.271972	1.992947
C	-1.802139	0.322412	3.305752
C	-1.533941	0.301989	4.733826
C	-0.514100	-0.528660	5.238813
C	-0.277924	-0.534460	6.610882
C	-1.020234	0.264594	7.466655
C	-2.022296	1.079290	6.959854
C	-2.300479	1.106089	5.597928
C	0.303860	-1.412499	4.334800
C	-3.385690	2.003724	5.066555
C	0.832993	2.117029	3.031197
C	-0.853822	-1.418114	-0.205382
H	0.498531	-1.178474	7.010256
H	-0.820135	0.250179	8.532159
H	-2.601013	1.704696	7.631350
H	3.014235	1.935578	1.462695
H	3.383490	0.485871	-0.501532
H	1.602468	-1.047378	-1.260273
H	-1.171945	-2.094408	0.592400
H	-1.744057	-0.875962	-0.536144
H	-0.494057	-2.021245	-1.040481
H	1.631436	2.858180	3.089956
H	-0.123170	2.643995	2.980279
H	0.830822	1.550063	3.966926
H	0.888822	-2.123037	4.920644
H	-0.330434	-1.974637	3.644724
H	0.998004	-0.830273	3.721444
H	-3.004353	2.681135	4.297976
H	-4.191430	1.423965	4.607737
H	-3.815183	2.605585	5.868977

$\{2DMP\}_2$

E= -2076.055014

C	2.306891	1.023741	0.441729
C	1.727729	-0.136588	0.603809
P	0.554902	1.225360	0.993489
P	-0.554903	1.225346	-0.993223
C	-2.307005	1.023700	-0.441846
C	-1.727787	-0.136619	-0.603801
C	3.569090	1.688431	0.166249
C	1.823301	-1.570621	0.394612
C	-1.823226	-1.570675	-0.394627

C	-3.569244	1.688317	-0.166332
C	2.439213	-2.053321	-0.776469
C	2.484420	-3.429287	-0.985254
C	1.948721	-4.309582	-0.058338
C	1.346338	-3.823863	1.092506
C	1.265527	-2.458315	1.333048
C	3.026480	-1.119578	-1.801209
H	2.949028	-3.811459	-1.888254
H	1.996512	-5.378336	-0.235706
H	0.920271	-4.513676	1.812757
C	0.621034	-1.945241	2.589865
C	4.763859	1.112472	0.645451
C	5.969361	1.756510	0.381161
C	6.004353	2.939118	-0.341053
C	4.824080	3.501531	-0.803972
C	3.596758	2.896115	-0.556697
C	4.761589	-0.160525	1.449366
H	6.891542	1.322463	0.752958
H	6.952512	3.426569	-0.538383
H	4.852112	4.428414	-1.367133
C	2.334050	3.523403	-1.082149
C	-2.439028	-2.053459	0.776473
C	-2.484059	-3.429430	0.985257
C	-1.948328	-4.309656	0.058297
C	-1.346071	-3.823860	-1.092579
C	-1.265395	-2.458299	-1.333100
C	-3.026310	-1.119776	1.801262
H	-2.948573	-3.811659	1.888282
H	-1.995975	-5.378417	0.235657
H	-0.919982	-4.513620	-1.812868
C	-0.620909	-1.945154	-2.589895

C	-4.763966	1.112261	-0.645515
C	-5.969537	1.756155	-0.381170
C	-6.004628	2.938741	0.341066
C	-4.824392	3.501267	0.803955
C	-3.597012	2.895986	0.556641
C	-4.761588	-0.160720	-1.449466
H	-6.891678	1.322012	-0.752953
H	-6.952824	3.426102	0.538442
H	-4.852510	4.428140	1.367128
C	-2.334375	3.523416	1.082109
H	1.639378	3.758841	-0.270496
H	2.556548	4.449751	-1.614150
H	1.809609	2.855543	-1.771135
H	5.742911	-0.333627	1.893709
H	4.020434	-0.121990	2.251806
H	4.511806	-1.030953	0.835478
H	3.279856	-1.661733	-2.713721
H	2.329719	-0.317093	-2.059577
H	3.935751	-0.634597	-1.433546
H	1.331860	-1.377633	3.197652
H	-0.206280	-1.270420	2.359255
H	0.232745	-2.769045	3.190709
H	0.206267	-1.270162	-2.359254
H	-0.232378	-2.768913	-3.190648
H	-1.331801	-1.377761	-3.197799
H	-3.280284	-1.662102	2.713505
H	-2.329282	-0.317689	2.060180
H	-3.935202	-0.634261	1.433394
H	-5.742889	-0.333880	-1.893831
H	-4.020420	-0.122114	-2.251892
H	-4.511736	-1.031141	-0.835594

H	-1.809840	2.855559	1.771015
H	-1.639764	3.759069	0.270470
H	-2.557004	4.449647	1.614271

8DMP

E= -1115.419567

C	-0.234539	0.410144	0.035610
C	0.023188	0.014548	1.357250
C	1.342046	-0.097242	1.823888
C	2.391895	0.180627	0.951904
C	2.145041	0.566537	-0.356816
C	0.839264	0.680302	-0.809189
C	-1.108666	-0.280641	2.271472
C	-1.693887	-1.489769	2.445437
C	-2.790942	-1.492825	3.411944
C	-3.041057	-0.286035	3.973933
C	-4.091600	0.003118	4.982289
C	-3.794324	-0.110437	6.349178
C	-4.792565	0.161813	7.281451
C	-6.061206	0.543910	6.872580
C	-6.345995	0.659418	5.520479
C	-5.371729	0.394844	4.561031
C	-2.418206	-0.519295	6.807848
C	-5.693519	0.529811	3.094918
C	1.624804	-0.510172	3.245422
C	-1.648811	0.543301	-0.467737
P	-1.903627	0.951180	3.320962
H	-1.382098	-2.385293	1.917210
H	-3.351470	-2.390776	3.652141
H	-4.568195	0.073705	8.339452
H	-6.829264	0.752456	7.609049
H	-7.337819	0.960920	5.199773
H	3.413444	0.093934	1.307425
H	2.971825	0.779431	-1.025317
H	0.645227	0.984770	-1.832477
H	-2.370916	-0.569878	7.896949
H	-2.137136	-1.495715	6.404973
H	-1.660480	0.193708	6.468230
H	-5.056130	1.280923	2.618037
H	-5.528056	-0.410068	2.562030
H	-6.733362	0.828233	2.951390
H	2.699395	-0.557610	3.429651
H	1.189699	0.198724	3.956759
H	1.193138	-1.488940	3.469914
H	-2.196247	-0.397994	-0.372964
H	-2.203692	1.291815	0.106456
H	-1.661023	0.844367	-1.516613

$\{8DMP\}_2$

E= -2230.937967

C	1.287043	-1.935547	-0.402150
C	0.633454	-3.007260	-0.900493
C	-0.792927	-3.012562	-0.678044
C	-1.285855	-1.937729	-0.021019
P	0.115312	-0.948565	0.592996
P	0.114958	0.948531	-0.593311
C	1.286447	1.935984	0.401675
C	0.632648	3.007739	0.899625
C	-0.793724	3.012665	0.677105
C	-1.286448	1.937472	0.020500
H	-1.429381	-3.827463	-1.007835
C	-2.732144	-1.794754	0.277142

H	1.140384	-3.801097	-1.439195
C	2.734152	-1.697870	-0.624324
H	1.139362	3.801832	1.438150
C	2.733488	1.698317	0.624275
H	-1.430378	3.827524	1.006623
C	-2.732792	1.794240	-0.277309
C	-3.613538	1.557060	0.791780
C	-4.983511	1.558300	0.550846
C	-5.482085	1.775125	-0.722882
C	-4.607200	1.990658	-1.776518
C	-3.229961	2.009886	-1.572840
C	-3.109459	1.325020	2.193735
H	-5.664375	1.366630	1.373211
H	-6.552525	1.769666	-0.896822
H	-4.996649	2.165677	-2.774472
C	-2.308012	2.294424	-2.730145
C	3.170953	1.388188	1.923944
C	4.537044	1.256269	2.161544
C	5.457428	1.437883	1.143233
C	5.017782	1.739274	-0.135972
C	3.661739	1.867243	-0.415986
C	2.202133	1.213145	3.068470
H	4.878057	1.011773	3.162518
H	6.518242	1.333338	1.342435
H	5.737348	1.871946	-0.936791
C	3.212450	2.212197	-1.810719
C	3.172028	-1.387512	-1.923794
C	4.538196	-1.255630	-2.160965
C	5.458270	-1.437494	-1.142416
C	5.018216	-1.739100	0.136598
C	3.662079	-1.867060	0.416187

C	2.203582	-1.212293	-3.068608
H	4.879519	-1.010978	-3.161795
H	6.519151	-1.332991	-1.341288
H	5.737538	-1.872007	0.937601
C	3.212384	-2.212202	1.810746
C	-3.613224	-1.557488	-0.791654
C	-4.983116	-1.558683	-0.550293
C	-5.481304	-1.775572	0.723583
C	-4.606102	-1.991239	1.776917
C	-3.228922	-2.010518	1.572798
C	-3.109583	-1.325378	-2.193742
H	-5.664237	-1.366924	-1.372422
H	-6.551692	-1.770068	0.897842
H	-4.995227	-2.166321	2.774986
C	-2.306640	-2.295113	2.729830
H	-1.589240	-3.081392	2.480310
H	-1.725043	-1.411840	3.009023
H	-2.874148	-2.613325	3.606217
H	-3.841227	-0.757177	-2.770595
H	-2.167066	-0.775229	-2.196821
H	-2.926923	-2.272650	-2.710607
H	-1.591274	3.081505	-2.481206
H	-1.725642	1.411484	-3.008765
H	-2.875847	2.611532	-3.606720
H	-3.840901	0.756782	2.770819
H	-2.166918	0.774904	2.196556
H	-2.926683	2.272310	2.710518
H	2.682916	1.375669	-2.272785
H	2.528323	3.065190	-1.808018
H	4.068068	2.456484	-2.442746
H	2.690131	0.711523	3.906961

H	1.830763	2.176952	3.428001
H	1.328666	0.625968	2.775367
H	4.067715	-2.457845	2.442639
H	2.683879	-1.375239	2.273221
H	2.527218	-3.064347	1.807651
H	2.691604	-0.709746	-3.906533
H	1.833052	-2.176108	-3.429004
H	1.329572	-0.625952	-2.775481

12DMP

E= -1154.732335

C	0.452167	0.176347	0.110833
C	0.115935	0.311991	1.466194
C	0.810949	-0.405875	2.450844
C	1.847268	-1.251204	2.061635
C	2.189197	-1.387890	0.724972
C	1.494277	-0.677521	-0.242018
C	-1.001152	1.224440	1.862969
P	-2.635212	0.472553	1.902380
C	-3.645761	1.889167	2.359414
C	-5.115919	1.627212	2.448304
C	-5.678359	1.224982	3.668848
C	-7.048908	0.984685	3.731418
C	-7.849000	1.135531	2.609227
C	-7.284021	1.529705	1.405869
C	-5.917276	1.778097	1.306589
C	-4.821643	1.056345	4.897454
C	-5.317322	2.205258	-0.008485
C	0.450709	-0.268268	3.907949
C	-0.294611	0.941442	-0.951613
C	-0.743052	2.512804	2.154410
C	-1.738799	3.548046	2.559295
C	-3.168296	3.122417	2.609652
H	-1.647478	4.414115	1.886110
H	-3.880933	3.900991	2.878137
H	0.289985	2.852574	2.095219
H	-1.452415	3.952656	3.543145
H	-7.490234	0.674345	4.672970
H	-8.914173	0.942592	2.671302
H	-7.909844	1.646959	0.527205
H	2.389512	-1.808436	2.818647
H	2.996403	-2.051500	0.435645
H	1.761032	-0.785024	-1.288315
H	-0.200967	2.020755	-0.803717
H	0.085303	0.697600	-1.945196
H	-1.363548	0.709463	-0.929758
H	1.070988	-0.921170	4.524266
H	0.585701	0.759954	4.254648
H	-0.597507	-0.527459	4.083523
H	-5.418860	0.709008	5.742178
H	-4.019688	0.332437	4.725468
H	-4.343607	1.998298	5.180086
H	-4.849577	3.190473	0.069682
H	-4.538729	1.508942	-0.333844
H	-6.080410	2.249194	-0.787440

{12DMP}$_2$

E= -2309.536675

C	1.287289	-1.466701	1.557322
C	0.759536	-2.096356	2.610012
C	-0.494577	-1.734045	3.349731
C	-1.050082	-0.358964	3.112495

C	-0.721095	0.471081	2.123818
P	0.611025	0.110662	0.924755
P	-0.610569	0.110263	-0.923266
C	-1.285224	-1.467671	-1.556109
C	-0.755308	-2.097985	-2.607302
C	0.500113	-1.735678	-3.344802
C	1.053028	-0.359255	-3.109168
C	0.721788	0.471570	-2.121898
H	1.285684	-2.472603	-3.114749
C	1.392966	1.787648	-1.889117
H	-1.305644	-2.945090	-3.015935
C	-2.649372	-1.823276	-1.048164
H	1.818312	-0.042457	-3.814898
H	0.311966	-1.863810	-4.417384
H	-0.303649	-1.859531	4.422155
C	2.650389	-1.822740	1.046910
H	-1.815164	-0.042396	3.818554
C	-1.394166	1.786036	1.890192
H	1.310902	-2.943012	3.018193
H	-1.279596	-2.472677	3.123337
C	-2.762139	1.811872	1.554639
C	-3.351497	3.025626	1.209833
C	-2.618650	4.202158	1.202531
C	-1.281383	4.175493	1.560073
C	-0.651604	2.981567	1.906085
C	-3.614773	0.568932	1.548944
H	-4.402392	3.042170	0.939403
H	-3.091835	5.138051	0.926230
H	-0.705718	5.095433	1.573230
C	0.808581	3.032038	2.285154
C	2.830320	-2.827023	0.085077

C	4.113125	-3.095119	-0.388073
C	5.208938	-2.396865	0.092412
C	5.029241	-1.421229	1.060543
C	3.760423	-1.115036	1.544782
C	1.662036	-3.620612	-0.429022
H	4.249001	-3.866275	-1.139479
H	6.202203	-2.614484	-0.284093
H	5.886366	-0.878518	1.445821
C	3.602255	-0.019389	2.566529
C	2.760802	1.815600	-1.553185
C	3.348436	3.030415	-1.209091
C	2.614013	4.205956	-1.202854
C	1.276877	4.177213	-1.560760
C	0.648810	2.982196	-1.906083
C	3.615260	0.573928	-1.546484
H	4.399229	3.048569	-0.938366
H	3.085857	5.142684	-0.927090
H	0.699994	5.096378	-1.574782
C	-0.811305	3.030542	-2.285682
C	-2.831101	-2.826096	-0.085122
C	-4.114881	-3.094224	0.385301
C	-5.209867	-2.397101	-0.098692
C	-5.028303	-1.422513	-1.067515
C	-3.758500	-1.116501	-1.549358
C	-1.663601	-3.617795	0.433668
H	-4.252177	-3.864397	1.137454
H	-6.203939	-2.614725	0.275678
H	-5.884760	-0.880644	-1.455458
C	-3.598809	-0.021905	-2.572043
H	-2.961829	-0.336016	-3.402072
H	-4.568500	0.275885	-2.974449

H	-3.130350	0.859403	-2.122185
H	-1.962242	-4.250373	1.272482
H	-1.249912	-4.265086	-0.344524
H	-0.855928	-2.963424	0.763536
H	4.524861	0.738162	-0.967131
H	3.094884	-0.282818	-1.117121
H	3.915298	0.293859	-2.560765
H	-1.073001	2.249224	-3.002151
H	-1.447016	2.904330	-1.403974
H	-1.046740	3.997106	-2.737084
H	2.972613	-0.335347	3.401510
H	4.573115	0.284153	2.961756
H	3.126025	0.858593	2.118304
H	1.959125	-4.255566	-1.266592
H	1.250161	-4.265729	0.351929
H	0.853477	-2.967467	-0.759075
H	-4.525928	0.732233	0.971782
H	-3.094030	-0.286664	1.117712
H	-3.912034	0.287513	2.563651
H	1.071577	2.251189	3.001659
H	1.444125	2.906514	1.403222
H	1.042861	3.999023	2.736255

21DMP

E= -1192.807045

C	-0.041108	-0.404832	0.097891
P	0.758277	-1.275801	1.452011
C	2.328263	-0.441688	1.750133
C	2.771548	0.803377	1.442950
C	2.119696	1.895457	0.760913
C	1.018135	1.909563	-0.018718
C	0.131251	0.842580	-0.412866
H	0.770259	2.870439	-0.462036
H	-0.539089	1.106513	-1.229466
C	-1.118447	-1.246155	-0.526578
C	3.275317	-1.332848	2.501639
H	3.774379	1.040277	1.795757
H	2.636545	2.846250	0.860418
C	4.151128	-2.161455	1.783843
C	5.028391	-2.981118	2.488846
C	5.039255	-2.984666	3.875317
C	4.169726	-2.162345	4.575123
C	3.277276	-1.329210	3.904295
H	5.709133	-3.623913	1.940381
H	5.726647	-3.629001	4.411924
H	4.178994	-2.164112	5.660228
C	2.338531	-0.439559	4.678345
C	-2.450085	-1.082045	-0.113648
C	-3.438749	-1.860738	-0.710024
C	-3.120911	-2.783122	-1.695200
C	-1.802762	-2.939807	-2.093545
C	-0.785670	-2.184124	-1.515389
C	-2.816271	-0.081903	0.953524
H	-4.470154	-1.739369	-0.395438
H	-3.902036	-3.380141	-2.152368
H	-1.552152	-3.660384	-2.865246
C	0.643067	-2.396288	-1.945965
H	1.181608	-2.995588	-1.204722
H	0.686417	-2.926995	-2.898717
H	1.180133	-1.451204	-2.050393
H	-3.876742	-0.151600	1.200635
H	-2.240518	-0.247609	1.868491

H	-2.608878	0.941597	0.629570
C	4.156912	-2.161462	0.276981
H	3.169493	-2.409532	-0.121919
H	4.418773	-1.176181	-0.118360
H	4.874012	-2.887701	-0.109101
H	2.486907	0.612630	4.421252
H	1.294160	-0.677964	4.456074
H	2.492095	-0.554876	5.752376

{21DMP}$_2$

E= -2385.690891

C	2.524332	2.346955	1.823466
C	2.436515	2.333122	0.416980
C	2.920812	3.436177	-0.320761
C	3.498912	4.506847	0.358346
C	3.610618	4.506727	1.738347
C	3.124216	3.429922	2.460364
C	1.793766	1.200673	-0.314271
P	0.060334	0.818864	0.166393
P	-0.178418	-1.443261	0.156903
C	-1.427443	-1.529806	1.528664
C	-2.856236	-1.495786	1.053926
C	-3.644875	-0.351197	1.267398
C	-4.987328	-0.375697	0.885110
C	-5.552585	-1.499985	0.313270
C	-4.771324	-2.628468	0.110343
C	-3.428890	-2.643364	0.471734
C	2.443860	0.591342	-1.323851
C	1.919702	-0.282623	-2.345233
C	0.705594	-0.197499	-2.930584
C	-0.391772	0.688288	-2.625023
C	-0.775936	1.185784	-1.432138
C	-2.029680	1.991203	-1.314651
C	-3.263820	1.398568	-1.639880
C	-4.433662	2.134667	-1.452156
C	-4.397478	3.428762	-0.962574
C	-3.175661	4.013595	-0.662666
C	-1.986073	3.312702	-0.830193
C	1.384853	-2.211315	0.800435
C	2.343462	-2.680983	-0.262012
C	3.662856	-2.174081	-0.284754
C	4.526484	-2.569079	-1.305621
C	4.128571	-3.464611	-2.283459
C	2.853099	-3.999288	-2.228653
C	1.956348	-3.627629	-1.229795
C	1.745375	-2.441826	2.077645
C	1.188533	-2.012222	3.344694
C	-0.055504	-1.628552	3.670228
C	-1.253738	-1.583764	2.859368
H	0.545469	-0.830499	-3.799766
H	-1.029110	0.923867	-3.476053
H	3.505512	0.812710	-1.431163
H	2.631749	-0.977631	-2.780617
H	1.908005	-2.037628	4.159231
H	2.674313	-2.997237	2.196295
H	-2.172122	-1.591313	3.445687
H	-0.220074	-1.382722	4.715926
C	4.215441	-1.219487	0.747197
H	5.531478	-2.160128	-1.327042
H	4.814854	-3.758340	-3.069798
H	2.541551	-4.732025	-2.965955
C	0.607364	-4.294998	-1.213721

C	-3.128112	0.906187	1.921849
H	-5.586586	0.515487	1.039662
H	-6.599336	-1.501906	0.029183
H	-5.210478	-3.520344	-0.325089
C	-2.627722	-3.897117	0.247020
C	-3.381177	-0.007078	-2.175665
H	-5.386373	1.669655	-1.683412
H	-5.318461	3.983123	-0.818970
H	-3.139787	5.033147	-0.292531
C	-0.679287	3.989439	-0.508933
C	2.824672	3.535039	-1.826100
H	3.861374	5.356820	-0.210711
H	4.067914	5.346789	2.249111
H	3.204963	3.423866	3.542691
C	1.978459	1.237124	2.684525
H	-0.825393	5.060695	-0.360131
H	-0.228097	3.585951	0.402914
H	0.046404	3.854689	-1.313728
H	-4.381122	-0.398619	-1.987582
H	-3.208142	-0.035790	-3.255954
H	-2.664288	-0.686115	-1.712089
H	-3.610861	1.781590	1.480426
H	-3.354108	0.900811	2.993691
H	-2.051977	1.028509	1.812599
H	-3.285375	-4.763376	0.152870
H	-2.042268	-3.817582	-0.671552
H	-1.929070	-4.084265	1.065608
H	2.567800	1.135523	3.598942
H	0.943116	1.440839	2.973350
H	1.978322	0.271698	2.183633
H	2.785227	4.583989	-2.127533
H	3.696649	3.087733	-2.312588
H	1.943877	3.028463	-2.222075
H	5.052397	-0.657404	0.328038
H	3.473328	-0.506114	1.094148
H	4.591281	-1.757517	1.622808
H	0.665196	-5.273800	-1.694648
H	0.239616	-4.437718	-0.196597
H	-0.129345	-3.691941	-1.749966

2DTBP

E= -1509.689969

C	-2.988933	-0.171216	0.042146
C	-1.691282	-0.009344	0.627430
C	-1.415711	1.141501	1.410870
C	-2.292452	2.224487	1.307250
C	-3.437739	2.161877	0.545573
C	-3.806220	0.953003	-0.021510
C	-0.610423	-0.937191	0.262007
C	0.610355	-0.937014	-0.262449
C	1.691407	-0.009256	-0.627390
C	1.416137	1.141680	-1.410743
C	2.293103	2.224486	-1.306951
C	3.438330	2.161534	-0.545234
C	3.806506	0.952530	0.021828
C	2.988932	-0.171445	-0.041879
C	0.301643	1.299566	-2.472860
C	-0.487734	0.025716	-2.786693
C	3.585515	-1.530274	0.413477
C	5.103792	-1.419207	0.660364
C	-0.301240	1.299082	2.473049
C	0.487850	0.025074	2.786687

C	-3.586009	-1.529819	-0.413235
C	-5.104480	-1.418473	-0.658759
P	-0.000216	-2.618840	-0.001323
C	-0.691079	2.395746	-2.054721
C	0.984499	1.707167	-3.800310
C	2.993177	-2.073003	1.725081
C	3.430853	-2.545995	-0.737374
C	0.691676	2.395167	2.055167
C	-0.984195	1.706603	3.800492
C	-2.994839	-2.071859	-1.725668
C	-3.430571	-2.546160	0.736921
H	2.068625	3.143336	-1.833326
H	4.081179	3.028775	-0.446390
H	4.765901	0.893111	0.513463
H	-2.067769	3.143206	1.833763
H	-4.080364	3.029293	0.446816
H	-4.765643	0.893805	-0.513117
H	0.226690	1.797319	-4.583779
H	1.505059	2.663590	-3.738257
H	1.708260	0.948523	-4.111154
H	-1.449889	2.529610	-2.832266
H	-1.199948	2.127436	-1.128662
H	-0.193645	3.357327	-1.902494
H	-1.038683	0.171608	-3.719990
H	0.160842	-0.845336	-2.916917
H	-1.218010	-0.193811	-2.017186
H	1.039169	0.170825	3.719787
H	-0.160941	-0.845792	2.917088
H	1.217788	-0.194641	2.016935
H	1.450262	2.529034	2.832929
H	1.200825	2.126800	1.129265
H	0.194364	3.356770	1.902699
H	-1.503912	2.663505	3.738677
H	-1.708704	0.948432	4.110736
H	-3.496887	-3.008121	-1.987768
H	-3.159713	-1.361821	-2.540515
H	-1.928256	-2.282404	-1.667502
H	-5.498259	-2.419317	-0.853269
H	-5.633724	-1.017569	0.209350
H	-5.343235	-0.804696	-1.531459
H	-3.855896	-3.509858	0.441248
H	-2.394942	-2.712202	1.024621
H	-3.969240	-2.194132	1.621357
H	3.494941	-3.009439	1.987078
H	3.157355	-1.363448	2.540479
H	1.926620	-2.283448	1.665794
H	3.856154	-3.509807	-0.442050
H	2.395388	-2.712001	-1.025724
H	3.969916	-2.193368	-1.621331
H	5.497188	-2.420125	0.855261
H	5.633892	-1.018436	-0.207281
H	5.341845	-0.805450	1.533275
H	-0.226568	1.795743	4.584246

$\{2DTBP\}_2$

E= -3019.420573

C	2.310298	0.563308	0.713027
C	2.225885	-0.668687	0.254520
P	0.622392	-0.132884	0.957864
P	-0.622530	-0.132787	-0.958140
C	-2.310337	0.563502	-0.713023
C	-2.225992	-0.668519	-0.254599

C	3.049039	1.827322	0.776184
C	2.885733	-1.847348	-0.339204
C	-2.885698	-1.847249	0.339151
C	-3.049099	1.827493	-0.776042
C	3.323558	-1.852399	-1.694529
C	4.082126	-2.947103	-2.122585
C	4.414305	-3.987370	-1.288449
C	3.912516	-4.011825	-0.001987
C	3.110527	-2.990748	0.497938
C	2.954700	-0.859848	-2.840075
H	4.432273	-2.986162	-3.142897
H	5.036482	-4.799669	-1.647227
H	4.149695	-4.865008	0.613467
C	2.444169	-3.245098	1.882499
C	4.383471	1.943831	0.289527
C	4.741361	3.146282	-0.316750
C	3.898419	4.241663	-0.306489
C	2.769880	4.203606	0.494072
C	2.350429	3.028493	1.112624
C	5.520282	0.919550	0.518842
H	5.710219	3.235195	-0.792459
H	4.173413	5.153244	-0.824602
H	2.216493	5.119434	0.651490
C	1.281995	3.147987	2.234149
C	-3.322690	-1.852600	1.694732
C	-4.080277	-2.947815	2.123265
C	-4.412665	-3.988083	1.289233
C	-3.912038	-4.012005	0.002299
C	-3.110754	-2.990586	-0.498023
C	-2.954271	-0.859706	2.840145
H	-4.429464	-2.987246	3.143897

H	-5.034100	-4.800778	1.648401
H	-4.149426	-4.865129	-0.613155
C	-2.445049	-3.244697	-1.882952
C	-4.383633	1.943849	-0.289646
C	-4.741750	3.146272	0.316557
C	-3.898905	4.241732	0.306482
C	-2.770192	4.203795	-0.493842
C	-2.350520	3.028729	-1.112331
C	-5.520204	0.919323	-0.519030
H	-5.710698	3.235100	0.792093
H	-4.174108	5.153282	0.824539
H	-2.216878	5.119681	-0.651165
C	-1.281974	3.148324	-2.233752
C	-0.160963	3.117595	1.711479
C	1.512322	2.097266	3.338988
C	1.458282	4.509850	2.947793
C	5.973523	0.249429	-0.783353
C	6.718699	1.698425	1.114092
C	5.172106	-0.152425	1.556977
C	4.100248	-0.733879	-3.873792
C	1.733971	-1.466172	-3.562503
C	2.614320	0.577929	-2.435715
C	0.944008	-3.514806	1.640151
C	2.991341	-4.520211	2.558326
C	2.660844	-2.135973	2.929851
C	-2.661949	-2.135422	-2.930074
C	-0.944829	-3.514637	-1.641204
C	-2.992624	-4.519624	-2.558811
C	-4.100224	-0.733760	3.873446
C	-1.733596	-1.465569	3.563063
C	-2.614073	0.578072	2.435651

C	-5.972638	0.248278	0.782948
C	-6.719247	1.697961	-1.113304
C	-5.171918	-0.151850	-1.557998
C	0.160994	3.117508	-1.711114
C	-1.512582	2.097938	-3.338858
C	-1.457891	4.510406	-2.947077
H	-1.457486	-0.839930	4.418680
H	-1.955400	-2.471724	3.930013
H	-0.870746	-1.520883	2.898338
H	-3.852472	0.067758	4.573394
H	-5.052233	-0.482855	3.400368
H	-4.239984	-1.633867	4.474085
H	-2.415588	1.153972	3.344165
H	-1.718409	0.641160	1.831148
H	-3.432841	1.067047	1.908222
H	-6.082935	-0.686248	-1.842195
H	-4.744445	0.293678	-2.460655
H	-4.477695	-0.891359	-1.178252
H	-6.863318	-0.360973	0.600817
H	-5.198836	-0.411120	1.171092
H	-6.219480	0.988782	1.549294
H	-7.507171	0.988730	-1.381897
H	-7.151715	2.415181	-0.414014
H	-6.423964	2.239350	-2.016239
H	0.868377	3.158560	-2.544589
H	0.355547	3.979646	-1.070245
H	0.388016	2.228890	-1.127049
H	-0.819575	4.527011	-3.834788
H	-2.491433	4.665591	-3.267237
H	-1.157508	5.356945	-2.326534
H	-0.822518	2.290629	-4.165680
H	-1.344113	1.073829	-3.016685
H	-2.532945	2.173654	-3.726248
H	0.819959	4.526441	3.835499
H	2.491869	4.664651	3.267995
H	1.158175	5.356627	2.327445
H	0.822655	2.290253	4.166071
H	1.343070	1.073330	3.016673
H	2.532857	2.172249	3.726065
H	-0.868363	3.158811	2.544931
H	-0.355267	3.979783	1.070603
H	-0.388172	2.229024	1.127407
H	7.506976	0.989464	1.382370
H	7.150893	2.416500	0.415515
H	6.422781	2.238899	2.017369
H	6.083318	-0.686395	1.841348
H	4.743854	0.292261	2.459675
H	4.478625	-0.892164	1.176322
H	6.864153	-0.359865	-0.601117
H	5.199973	-0.409794	-1.172324
H	6.220719	0.990403	-1.549123
H	-2.168595	-2.428152	-3.861300
H	-3.727491	-2.012254	-3.139927
H	-2.260631	-1.168790	-2.647662
H	-0.439428	-3.708513	-2.592194
H	-0.434866	-2.681811	-1.161807
H	-0.822316	-4.393626	-1.002566
H	-2.499169	-4.634278	-3.527038
H	-2.783031	-5.425832	-1.986067
H	-4.068958	-4.457069	-2.743241
H	2.416035	1.153831	-3.344271
H	1.718494	0.640970	-1.831432

H	3.432909	1.066960	-1.908074
H	1.457446	-0.840791	-4.418177
H	1.955960	-2.472351	-3.929281
H	0.871317	-1.521578	-2.897542
H	3.851812	0.067101	-4.574107
H	5.052278	-0.482172	-3.401168
H	4.240283	-1.634256	-4.473966
H	2.497269	-4.635181	3.526200
H	2.782150	-5.426249	1.985170
H	4.067555	-4.457674	2.743464
H	2.168206	-2.429340	3.861255
H	3.726405	-2.012051	3.139144
H	2.258570	-1.169585	2.647937
H	0.438139	-3.708325	2.590967
H	0.434386	-2.682050	1.160271
H	0.821613	-4.393964	1.001721

8DTBP

E= -1587.131749

C	3.399881	-1.330725	-0.106417
C	2.766817	-0.064340	0.051280
C	3.530574	1.138717	0.031108
C	4.914012	1.028562	-0.118315
C	5.538543	-0.193483	-0.251618
C	4.788439	-1.349966	-0.250891
C	1.294494	-0.008031	0.297911
C	0.727300	-0.001362	1.527553
C	-0.727395	0.001609	1.527581
C	-1.294582	0.008038	0.297980
C	-2.766892	0.064330	0.051344
C	-3.399968	1.330727	-0.106321
C	-4.788532	1.349962	-0.250554
C	-5.538643	0.193465	-0.251121
C	-4.914097	-1.028575	-0.117953
C	-3.530612	-1.138716	0.031244
C	-2.670829	2.704969	-0.205636
C	-2.028617	2.832737	-1.599556
C	-2.948751	-2.577174	0.170855
C	-4.037724	-3.652174	-0.029983
C	2.948840	2.577203	0.170983
C	2.373179	2.822876	1.579464
C	2.670767	-2.705005	-0.205436
C	3.662298	-3.881950	-0.077780
P	0.000005	-0.000124	-0.960538
C	-3.662297	3.881973	-0.078068
C	-1.611459	2.952455	0.888827
C	-1.883364	-2.872845	-0.899138
C	-2.373022	-2.823056	1.579259
C	4.037915	3.652143	-0.029625
C	1.883498	2.873271	-0.898911
C	1.611573	-2.952354	0.889212
C	2.028439	-2.833060	-1.599288
H	-1.304375	0.025935	2.445956
H	1.304311	-0.025525	2.445911
H	-5.311758	2.286441	-0.362856
H	-6.616199	0.244985	-0.361479
H	-5.534152	-1.911137	-0.130528
H	5.534083	1.911115	-0.130959
H	6.616082	-0.244978	-0.362161
H	5.311634	-2.286455	-0.363238
H	-1.526433	3.800943	-1.696035
H	-1.287024	2.056167	-1.792306

H	-2.791449	2.762185	-2.379847
H	-1.357337	4.016166	0.906137
H	-1.994400	2.682396	1.876883
H	-0.687678	2.408342	0.724864
H	-3.099990	4.818140	-0.111215
H	-4.382213	3.920168	-0.897939
H	-4.208447	3.854144	0.869197
H	-1.614486	-3.933194	-0.869652
H	-2.258670	-2.643201	-1.900117
H	-0.964008	-2.313329	-0.745250
H	-2.136595	-3.884984	1.699595
H	-1.460384	-2.261143	1.759668
H	-3.104327	-2.551220	2.345837
H	-3.568348	-4.636724	0.035285
H	-4.809891	-3.612928	0.742504
H	-4.514985	-3.579099	-1.010787
H	1.613588	3.933318	-0.868022
H	2.259375	2.645372	-1.900075
H	0.964662	2.312609	-0.746137
H	2.136944	3.884824	1.700012
H	1.460428	2.261121	1.759782
H	3.104435	2.550753	2.345982
H	3.568594	4.636724	0.035607
H	4.809969	3.612785	0.742968
H	4.515312	3.579112	-1.010366
H	1.526748	-3.801525	-1.695744
H	1.286410	-2.056878	-1.791935
H	2.791140	-2.762052	-2.379663
H	3.100017	-4.818146	-0.110524
H	4.382023	-3.920360	-0.897814
H	4.208683	-3.853842	0.869342
H	1.357188	-4.016002	0.906448
H	1.994840	-2.682518	1.877207
H	0.687903	-2.407976	0.725584

{8DTBP}$_2$

E= -3174.310817

C	0.419261	-0.594129	-2.371483
P	-0.607402	-0.267221	-0.935893
P	0.581221	-0.193296	0.869473
C	1.846930	0.796459	1.747718
C	1.534394	0.639306	3.062692
C	0.298645	0.003976	3.385082
C	-0.429567	-0.447331	2.332574
C	-0.305408	-0.230376	-3.457780
C	-1.557002	0.405558	-3.176169
C	-1.883289	0.640069	-1.876105
H	-2.268130	0.616009	-3.966127
C	-3.245082	1.149014	-1.487275
H	0.001175	-0.456234	-4.470995
C	1.744730	-1.274756	-2.387239
H	-0.012109	-0.157953	4.409199
C	-1.767082	-1.087313	2.530260
H	2.236250	0.930450	3.834372
C	3.219393	1.263698	1.349259
C	-2.897181	-0.236999	2.703503
C	-4.138014	-0.839505	2.928441
C	-4.280116	-2.205938	3.019914
C	-3.168015	-3.016201	2.918518
C	-1.891680	-2.501123	2.690615
C	-2.878555	1.318095	2.816923
H	-5.022070	-0.232823	3.044988

H	-5.258095	-2.643658	3.187032
H	-3.315178	-4.078267	3.030625
C	-0.720227	-3.529264	2.685634
C	3.471568	2.665183	1.228515
C	4.746794	3.077783	0.842681
C	5.761353	2.177518	0.604234
C	5.538543	0.835492	0.816309
C	4.300953	0.334042	1.230829
C	2.468794	3.795270	1.596865
H	4.965565	4.126637	0.722086
H	6.735173	2.524301	0.276287
H	6.370543	0.166504	0.668936
C	4.329425	-1.155697	1.708943
C	1.830053	-2.694244	-2.365350
C	3.098544	-3.260875	-2.213128
C	4.238513	-2.491434	-2.133786
C	4.151742	-1.121339	-2.278262
C	2.925483	-0.478546	-2.442487
C	0.694663	-3.698755	-2.726343
H	3.210920	-4.332924	-2.164422
H	5.203750	-2.965187	-1.992903
H	5.066789	-0.549276	-2.263302
C	2.964769	1.023951	-2.841957
C	-4.333343	0.252635	-1.229190
C	-5.540546	0.812329	-0.802342
C	-5.735139	2.171877	-0.702042
C	-4.723335	3.027820	-1.073547
C	-3.474167	2.560244	-1.481859
C	-4.366940	-1.291636	-1.477856
H	-6.372076	0.174887	-0.550762
H	-6.686129	2.563946	-0.358675
H	-4.924152	4.086507	-1.039755
C	-2.469362	3.640005	-1.976805
C	3.031600	5.195985	1.280324
C	1.158831	3.702317	0.817943
C	2.181930	3.791984	3.113712
C	4.427441	-1.149265	3.248658
C	3.141479	-2.033872	1.295222
C	5.589688	-1.893409	1.203192
C	0.973700	-5.099415	-2.137931
C	-0.732747	-3.350014	-2.287764
C	0.704744	-3.809861	-4.265007
C	2.663872	1.130849	-4.350574
C	1.998199	1.922261	-2.059169
C	4.365358	1.628881	-2.636436
C	-3.474198	-2.079973	-0.513469
C	-5.783060	-1.874338	-1.268354
C	-4.017279	-1.617861	-2.942603
C	-1.141536	3.587617	-1.224171
C	-2.228582	3.513556	-3.495973
C	-3.002239	5.071218	-1.757223
C	-0.112740	-3.681673	1.284035
C	0.366291	-3.182039	3.722820
C	-1.200055	-4.944151	3.076143
C	-4.280459	1.911465	2.561619
C	-2.490535	1.697510	4.260163
C	-1.945332	2.044504	1.840045
H	-3.608787	-3.153626	-0.681451
H	-2.421358	-1.858209	-0.634636
H	-3.747766	-1.861979	0.517503
H	-4.219773	-2.675214	-3.137154
H	-4.627990	-1.022931	-3.628668

H	-2.972095	-1.441558	-3.171823	H	-0.035661	-4.545990	-4.594830
H	-5.746001	-2.941450	-1.499762	H	0.457991	-2.849811	-4.725456
H	-6.119435	-1.785723	-0.232019	H	1.686908	-4.118895	-4.632734
H	-6.528513	-1.420918	-1.927302	H	-1.387630	-4.196681	-2.514015
H	-1.765496	4.432282	-3.868724	H	-0.789553	-3.162609	-1.219631
H	-1.559195	2.692309	-3.738526	H	-1.136142	-2.489169	-2.808929
H	-3.171605	3.369587	-4.031118	H	0.108915	-5.741477	-2.320823
H	-2.226038	5.776101	-2.065552	H	1.830853	-5.595850	-2.596093
H	-3.891274	5.287505	-2.355644	H	1.133879	-5.050062	-1.057680
H	-3.225050	5.271734	-0.705792	H	2.806089	2.165189	-4.680241
H	-0.445311	4.312718	-1.655946	H	3.340534	0.493527	-4.926670
H	-1.294367	3.849298	-0.177208	H	1.639660	0.846358	-4.583954
H	-0.666173	2.612696	-1.260403	H	2.235887	2.971220	-2.260789
H	-2.152021	3.117782	1.885706	H	0.960237	1.770190	-2.336555
H	-0.895187	1.915505	2.078070	H	2.107368	1.764963	-0.986783
H	-2.124009	1.725631	0.813214	H	4.324658	2.693757	-2.878529
H	-2.615795	2.775837	4.402674	H	4.697243	1.537174	-1.602549
H	-3.128938	1.181853	4.982863	H	5.114892	1.181814	-3.294378
H	-1.451668	1.452198	4.479162	H	0.457212	4.454279	1.191240
H	-4.200691	3.000319	2.521041	H	1.332028	3.900952	-0.239766
H	-4.690468	1.574060	1.608985	H	0.677993	2.732979	0.904086
H	-4.991305	1.679062	3.357857	H	1.712548	4.739277	3.396150
H	0.709262	-4.404725	1.311904	H	1.501017	2.994504	3.400328
H	-0.870695	-4.061821	0.595176	H	3.107676	3.689059	3.687221
H	0.285191	-2.753487	0.879834	H	2.257011	5.934028	1.503497
H	1.066372	-4.018546	3.806054	H	3.903360	5.448449	1.889866
H	0.941414	-2.300991	3.461552	H	3.292540	5.307584	0.224393
H	-0.082540	-3.017249	4.707099	H	2.252776	-1.816920	1.877147
H	-0.334754	-5.611544	3.081129	H	2.898037	-1.926487	0.238680
H	-1.643236	-4.971098	4.075336	H	3.387001	-3.083917	1.479279
H	-1.916199	-5.356177	2.360856	H	5.516555	-2.943122	1.495627

H	5.673415	-1.856976	0.115478
H	6.511611	-1.508132	1.644275
H	4.578236	-2.171051	3.611933
H	5.269955	-0.538524	3.585070
H	3.517637	-0.766861	3.709320

12DTBP

E= -1626.437145

C	3.243920	-1.329575	-0.387453
C	2.767454	-0.036105	-0.050736
C	3.537374	1.124125	-0.372623
C	4.719400	0.943339	-1.087946
C	5.150004	-0.305757	-1.490553
C	4.425669	-1.417366	-1.131991
C	1.408699	0.118089	0.579072
P	0.026773	0.072928	-0.590525
C	-1.399377	0.169796	0.527053
C	-1.279675	0.328700	1.855573
C	-0.018601	0.394956	2.650039
C	1.264712	0.232048	1.908203
C	3.228003	2.580887	0.084851
C	1.764616	3.014485	-0.095769
C	2.598067	-2.715415	-0.080546
C	1.983441	-3.250568	-1.388210
C	-2.774025	0.052152	-0.089482
C	-3.370933	-1.234313	-0.253431
C	-4.615708	-1.299511	-0.883243
C	-5.273086	-0.174066	-1.328620
C	-4.712124	1.066864	-1.121856
C	-3.473949	1.226915	-0.497038
C	-2.754435	-2.603907	0.171324
C	-3.810086	-3.732290	0.160934
C	-3.038014	2.697253	-0.219308
C	-3.263229	3.016493	1.271621
C	3.620444	2.729675	1.567374
C	4.063731	3.613562	-0.701398
C	3.693383	-3.697112	0.401422
C	1.524110	-2.776068	1.016690
C	-1.581310	3.006508	-0.591192
C	-3.888666	3.709151	-1.016342
C	-1.675639	-3.018441	-0.842507
C	-2.184258	-2.621867	1.604738
H	0.006485	1.346482	3.205859
H	-2.192072	0.357227	2.447359
H	2.156651	0.185741	2.528853
H	-0.070239	-0.368682	3.441949
H	5.326801	1.792846	-1.358095
H	6.059306	-0.408221	-2.072212
H	4.788289	-2.384255	-1.451465
H	-5.266807	1.928232	-1.458403
H	-6.233769	-0.263809	-1.823408
H	-5.098067	-2.251451	-1.036585
H	3.228351	-4.646672	0.679969
H	4.444062	-3.924314	-0.355592
H	4.207959	-3.300466	1.281039
H	1.253204	-3.824649	1.171576
H	1.890597	-2.379621	1.966236
H	0.614875	-2.248411	0.753314
H	1.521251	-4.228789	-1.220537
H	1.216263	-2.570118	-1.765040
H	2.742488	-3.361052	-2.166842
H	3.492561	3.769893	1.884782

H	3.000804	2.105657	2.211229
H	4.666385	2.450973	1.722239
H	3.763114	4.616977	-0.390483
H	5.134695	3.531768	-0.504358
H	3.897921	3.532924	-1.779265
H	1.688871	4.099874	0.016248
H	1.383636	2.753027	-1.086262
H	1.112519	2.571970	0.650677
H	-1.224477	-3.972618	-0.551431
H	-2.113328	-3.132847	-1.838136
H	-0.877860	-2.281309	-0.918886
H	-3.340413	-4.648578	0.526499
H	-4.657143	-3.506880	0.815276
H	-4.187974	-3.952391	-0.839466
H	-1.983022	-3.656756	1.895722
H	-1.254034	-2.074662	1.704146
H	-2.905330	-2.207341	2.315525
H	-3.065323	4.076895	1.459160
H	-4.296775	2.806212	1.560723
H	-2.601925	2.434866	1.912962
H	-1.415480	4.087265	-0.553728
H	-0.879805	2.555364	0.100519
H	-1.342912	2.662547	-1.601010
H	-3.496290	4.712109	-0.832323
H	-3.838303	3.525889	-2.093260
H	-4.936815	3.719408	-0.709101

{12DTBP}$_2$

E= -3252.857537

C	-3.555823	-2.706845	-1.035884
C	-2.924125	-2.337769	0.185376
C	-3.721970	-2.257125	1.378405
C	-5.092848	-2.488191	1.278014
C	-5.717986	-2.718854	0.071285
C	-4.949934	-2.818936	-1.060586
C	-1.428528	-2.069792	0.248552
C	-0.646138	-3.004974	0.781599
C	0.821639	-2.876664	0.982948
C	1.565503	-2.145841	-0.081159
C	1.061350	-1.359840	-1.046005
C	2.036396	-1.032661	-2.146749
C	3.171605	-1.910003	-2.268795
C	4.402858	-1.339953	-2.597738
C	4.504389	-0.030923	-3.016949
C	3.331204	0.643593	-3.305295
C	2.075774	0.147358	-2.963362
C	3.161743	-3.473893	-2.321311
C	1.776692	-4.129206	-2.287084
C	0.865131	0.790527	-3.694808
C	1.332338	1.433524	-5.021082
C	-3.225707	-1.911993	2.814792
C	-2.451536	-0.589762	2.861465
C	-2.917124	-2.992314	-2.425897
C	-3.121558	-1.752236	-3.314157
P	-0.624074	-0.613365	-0.653239
P	0.289323	1.155994	0.511887
C	-1.104636	2.361683	0.904256
C	-1.696896	3.201986	-0.231879
C	-2.945448	2.888267	-0.867050
C	-3.227537	3.458880	-2.105346
C	-2.379051	4.365205	-2.707106
C	-1.306946	4.828058	-1.988073

C	-0.974480	4.330453	-0.721979	C	-4.395189	-1.693812	3.803635
C	-4.079697	2.041092	-0.240530	C	-2.415156	-3.074209	3.423753
C	-4.504768	2.625737	1.120836	C	-1.443014	-3.400555	-2.418940
C	0.159969	5.160499	-0.043958	C	-3.628803	-4.193981	-3.100275
C	-0.096000	6.670843	-0.294832	H	1.008926	-2.402402	1.949795
C	-1.374540	2.680072	2.169705	H	-1.103210	-3.933237	1.112443
C	-0.686800	2.223138	3.415496	H	2.625984	-2.349163	-0.092643
C	0.607443	1.490602	3.258887	H	1.279064	-3.868856	1.076873
C	1.089812	0.862604	2.187281	H	-0.492872	3.121492	4.016249
C	2.422048	0.122158	2.217163	H	-2.087929	3.482272	2.337364
C	2.617672	-1.087909	2.964763	H	1.223843	1.497042	4.153325
C	3.667614	-1.928872	2.582006	H	-1.372672	1.625969	4.033894
C	4.520341	-1.627357	1.538918	H	-4.135545	3.198059	-2.624974
C	4.466621	-0.364442	0.997836	H	-2.590712	4.751129	-3.697924
C	3.507315	0.563255	1.402644	H	-0.700193	5.606547	-2.426756
C	1.899654	-1.501313	4.289924	H	3.812933	-2.871390	3.084813
C	0.379645	-1.283932	4.343797	H	5.262618	-2.344585	1.207501
C	3.879656	2.027415	1.058573	H	5.209608	-0.073505	0.266776
C	5.318541	2.263761	1.583219	H	5.295889	-1.953713	-2.576240
C	-5.357179	2.023037	-1.103350	H	5.468353	0.415304	-3.233652
C	-3.667073	0.589831	-0.082652	H	3.399494	1.591434	-3.820519
C	0.259923	5.074053	1.487355	H	-5.715092	-2.454548	2.156887
C	1.495200	4.782605	-0.698010	H	-6.794566	-2.837434	0.021583
C	2.109634	-2.996527	4.623619	H	-5.450688	-3.012321	-1.997453
C	2.575700	-0.702607	5.423927	H	5.593325	3.309526	1.414821
C	3.867713	2.260366	-0.455289	H	5.379603	2.063218	2.656756
C	3.031005	3.079584	1.765159	H	6.062693	1.643964	1.081857
C	3.727563	-3.840819	-3.719155	H	4.104227	3.304708	-0.681895
C	4.062413	-4.127835	-1.259329	H	4.605981	1.633041	-0.956235
C	-0.109963	-0.321701	-4.104272	H	2.896328	2.015399	-0.885782
C	0.144785	1.898580	-2.922111	H	3.452773	4.069874	1.573561

H	2.013719	3.085649	1.402718
H	3.012010	2.919746	2.846105
H	2.122015	-0.958178	6.387281
H	3.643774	-0.930356	5.473929
H	2.476219	0.374190	5.275741
H	1.560844	-3.228199	5.539243
H	1.729330	-3.650583	3.833267
H	3.152757	-3.250509	4.816259
H	-0.066236	-1.975072	5.062648
H	0.105729	-0.280627	4.655349
H	-0.089572	-1.461024	3.381259
H	4.158867	-5.198644	-1.464007
H	5.067227	-3.697960	-1.259348
H	3.654067	-4.021363	-0.251654
H	1.874633	-5.175887	-2.589539
H	1.301640	-4.117895	-1.309709
H	1.104534	-3.636603	-2.990516
H	3.681892	-4.924212	-3.864348
H	3.136805	-3.364154	-4.506170
H	4.765517	-3.532642	-3.848968
H	-0.967787	0.107237	-4.631130
H	0.381024	-1.039537	-4.768845
H	-0.494198	-0.859983	-3.244903
H	0.445864	1.665840	-5.617949
H	1.864679	2.375601	-4.867819
H	1.968757	0.762254	-5.603306
H	-0.524537	2.441557	-3.594395
H	-0.468090	1.517771	-2.110934
H	0.858685	2.614195	-2.509136
H	2.326704	5.321768	-0.233657
H	1.682114	3.715466	-0.606151
H	1.485968	5.024473	-1.764638
H	1.033283	5.770734	1.823074
H	-0.683994	5.375159	1.950755
H	0.524202	4.097214	1.865452
H	0.611494	7.255229	0.299541
H	0.044162	6.973966	-1.332182
H	-1.107963	6.952814	0.008431
H	-5.434880	2.151487	1.448720
H	-3.759732	2.444870	1.891015
H	-4.678758	3.703365	1.048859
H	-6.105844	1.416946	-0.586649
H	-5.781077	3.020530	-1.249362
H	-5.192848	1.559505	-2.079410
H	-4.467631	0.018016	0.390299
H	-3.463120	0.143818	-1.055525
H	-2.777021	0.494044	0.531240
H	-3.083373	-4.466343	-4.007581
H	-3.640878	-5.063891	-2.437751
H	-4.653994	-3.984833	-3.406191
H	-2.687732	-1.916508	-4.305658
H	-4.186798	-1.538230	-3.439169
H	-2.648675	-0.870189	-2.880100
H	-1.153752	-3.673532	-3.437587
H	-0.763752	-2.620275	-2.102513
H	-1.270729	-4.266513	-1.774862
H	-2.285427	-2.911221	4.497932
H	-2.949167	-4.018913	3.286597
H	-1.425031	-3.175675	2.992268
H	-2.087362	-0.430642	3.878174
H	-1.594799	-0.549519	2.192640
H	-3.114159	0.240879	2.612729

H	-3.974966	-1.362140	4.756632
H	-5.088426	-0.920133	3.463759
H	-4.955740	-2.610321	4.004467

21DTBP

E= -1664.512869

C	-3.076267	1.459955	-0.745782
C	-2.700549	0.239193	-0.124709
C	-3.436282	-0.960830	-0.371148
C	-4.472736	-0.902470	-1.301782
C	-4.793044	0.260366	-1.973330
C	-4.113059	1.419933	-1.683022
C	-1.462707	0.169996	0.739415
C	-1.614903	0.262344	2.084499
C	-0.664495	0.125585	3.158179
C	0.664690	-0.124530	3.158106
C	1.614948	-0.261621	2.084351
C	1.462703	-0.169628	0.739229
C	2.700580	-0.239235	-0.124844
C	3.436759	0.960520	-0.371202
C	4.473584	0.901705	-1.301410
C	4.793720	-0.261282	-1.972741
C	4.113185	-1.420581	-1.682627
C	3.076085	-1.460210	-0.745734
C	3.260633	2.319904	0.374747
C	2.436423	-2.868166	-0.550155
C	-3.260171	-2.319947	0.375195
C	-2.437173	2.868142	-0.550294
P	-0.000014	0.000286	-0.306994
H	-1.114071	0.214856	4.143544
H	-2.629202	0.455582	2.423982
H	2.629241	-0.454975	2.423795
H	1.114406	-0.213572	4.143427
H	5.050510	1.784340	-1.527873
H	5.586048	-0.262677	-2.712833
H	4.395259	-2.320147	-2.209683
H	-5.049167	-1.785380	-1.528456
H	-5.585060	0.261401	-2.713757
H	-4.395235	2.319363	-2.210276
C	-1.441085	3.116263	-1.698062
C	-3.531417	3.960799	-0.614506
C	-1.727277	3.109336	0.791782
C	-3.979344	-3.468473	-0.365460
C	-1.806694	-2.794455	0.539263
C	-3.919898	-2.211113	1.764077
C	1.807063	2.794495	0.538156
C	3.919984	2.211502	1.763822
C	3.980218	3.468115	-0.366023
C	1.727522	-3.109208	0.792487
C	1.439425	-3.115581	-1.697263
C	3.529979	-3.961472	-0.615493
H	0.985787	-4.107262	-1.597347
H	0.642876	-2.368766	-1.695303
H	1.941762	-3.063937	-2.667109
H	1.451913	-4.165950	0.854001
H	2.382873	-2.884393	1.637822
H	0.814849	-2.536351	0.904976
H	3.084157	-4.925304	-0.356975
H	3.970774	-4.080193	-1.605936
H	4.334744	-3.758217	0.096521
H	-1.804429	-3.827402	0.899346
H	-1.271193	-2.772330	-0.412570

H	-1.251116	-2.206255	1.263993
H	-3.921797	-3.190547	2.253343
H	-3.384759	-1.518280	2.412628
H	-4.955379	-1.871053	1.675249
H	-3.766029	-4.406124	0.153112
H	-5.065481	-3.355095	-0.372017
H	-3.629478	-3.569874	-1.396511
H	-1.451599	4.166082	0.852931
H	-2.381948	2.884642	1.637676
H	-0.814525	2.536461	0.903656
H	-0.987709	4.108057	-1.598124
H	-0.644301	2.369698	-1.696813
H	-1.944056	3.064767	-2.667583
H	-3.085957	4.924870	-0.356227
H	-3.973209	4.079425	-1.604513
H	-4.335405	3.756943	0.098212
H	3.921488	3.191025	2.252919
H	3.384909	1.518626	2.412367
H	4.955599	1.871742	1.675332
H	3.766766	4.405971	0.152112
H	5.066345	3.354604	-0.371986
H	3.630889	3.569187	-1.397285
H	1.804565	3.828411	0.895397
H	1.271128	2.769621	-0.413346
H	1.252028	2.208030	1.264759

{21DTBP}$_2$

E= -3329.013055

C	0.847450	-3.834590	-1.841472
C	1.411698	-3.236728	-0.672663
C	2.850799	-3.169876	-0.585467
C	3.591921	-3.465076	-1.733214
C	3.015298	-3.826078	-2.931002
C	1.658760	-4.038773	-2.959862
C	0.555717	-2.714384	0.491872
C	0.632580	-3.469814	1.601962
C	0.252067	-3.277994	2.988586
C	-0.666155	-2.490136	3.558676
C	-1.692989	-1.689790	2.932925
C	-1.843744	-1.183952	1.702687
C	-3.199001	-0.523053	1.452869
C	-3.441726	0.848497	1.778465
C	-4.589037	1.453803	1.258092
C	-5.552628	0.751990	0.577447
C	-5.415490	-0.613149	0.468256
C	-4.282060	-1.291290	0.919376
C	-2.736308	1.783823	2.811136
C	-4.416511	-2.837890	0.764473
C	3.775613	-2.959432	0.666210
C	-0.587550	-4.388202	-2.042249
P	-0.693794	-1.308232	0.189871
P	0.164510	0.888614	0.180770
C	-0.421127	1.636461	-1.455104
C	-1.489090	2.734161	-1.439427
C	-2.837410	2.407326	-1.812362
C	-3.842995	3.316454	-1.465806
C	-3.581112	4.535643	-0.886039
C	-2.269778	4.934713	-0.752358
C	-1.208925	4.085491	-1.055492
C	-3.323424	1.227255	-2.723564
C	0.189351	4.751610	-1.116131
C	2.019254	0.712424	-0.104975

C	2.404171	-0.128979	-1.093359
C	1.697574	-0.641453	-2.240793
C	0.741046	-0.018332	-2.949230
C	0.031001	1.196309	-2.641743
C	3.217233	1.260857	0.675537
C	3.398751	1.226098	2.105392
C	4.694908	1.107338	2.601134
C	5.813696	1.117024	1.794359
C	5.646767	1.410625	0.465237
C	4.382843	1.548064	-0.117619
C	2.304198	1.365036	3.196923
C	4.493115	2.138568	-1.564271
H	0.477458	-0.459518	-3.908056
H	-0.295190	1.750275	-3.514965
H	3.446462	-0.428329	-1.098054
H	2.132386	-1.525413	-2.686999
H	0.843290	-3.885932	3.669825
H	1.212902	-4.379839	1.471075
H	-2.542685	-1.524342	3.592598
H	-0.730992	-2.531460	4.642535
H	4.667381	-3.406398	-1.701125
H	3.624939	-3.991183	-3.812125
H	1.214302	-4.403199	-3.874561
H	-4.747455	2.510622	1.421449
H	-6.430926	1.253585	0.186475
H	-6.218552	-1.165656	0.002832
H	-4.874570	3.070428	-1.655682
H	-4.392399	5.196904	-0.601718
H	-2.076475	5.937133	-0.403928
H	6.529965	1.547732	-0.143920
H	6.801529	0.969117	2.215637
H	4.848837	0.986190	3.661183
C	0.138676	6.271676	-0.858387
C	1.148744	4.204867	-0.059134
C	0.719273	4.606584	-2.555871
C	-4.865028	1.163945	-2.824933
C	-2.864635	1.485157	-4.177942
C	-2.896224	-0.164633	-2.261404
C	-2.271030	3.089257	2.147047
C	-3.787858	2.108724	3.900385
C	-1.534226	1.228578	3.559239
C	-5.836831	-3.275700	1.209170
C	-4.250888	-3.176815	-0.724273
C	-3.489660	-3.715039	1.620845
C	2.899717	1.658426	4.592811
C	1.452195	2.601015	2.872921
C	1.499805	0.072512	3.375915
C	5.177154	3.519908	-1.398407
C	5.357943	1.247239	-2.477032
C	3.198082	2.425230	-2.320793
C	3.220737	-2.108802	1.811717
C	4.114510	-4.365227	1.206320
C	5.125034	-2.292610	0.292265
C	-0.535673	-5.729383	-2.816682
C	-1.292203	-4.727386	-0.731885
C	-1.407280	-3.388591	-2.869908
H	-2.235250	-5.236127	-0.941695
H	-0.679157	-5.386065	-0.111132
H	-1.524021	-3.842092	-0.159690
H	-2.413220	-3.776489	-3.058453
H	-1.491286	-2.434171	-2.352169
H	-0.928577	-3.208091	-3.837122

H	-1.528475	-6.186442	-2.790662
H	-0.272119	-5.618161	-3.869424
H	0.170503	-6.426588	-2.357548
H	-3.719580	-4.764020	1.412442
H	-2.433351	-3.574448	1.438851
H	-3.665892	-3.540791	2.685753
H	-4.353092	-4.253608	-0.891083
H	-5.009012	-2.665691	-1.323888
H	-3.277259	-2.858289	-1.089520
H	-5.878879	-4.367743	1.243246
H	-6.067821	-2.896054	2.208047
H	-6.627537	-2.954083	0.531108
H	-1.143120	2.016437	4.208665
H	-1.788123	0.382250	4.196218
H	-0.750052	0.936562	2.875271
H	-3.319232	2.703316	4.690921
H	-4.638416	2.675411	3.518804
H	-4.173446	1.188752	4.350030
H	-1.883254	3.772490	2.909897
H	-1.472350	2.888756	1.433392
H	-3.067186	3.606163	1.612736
H	0.807465	2.856047	3.716183
H	2.108459	3.453867	2.678475
H	0.815095	2.449565	2.007782
H	0.754201	0.192789	4.163014
H	0.983637	-0.257542	2.475623
H	2.168230	-0.733067	3.682515
H	2.070232	1.864180	5.274298
H	3.447718	0.810816	5.011893
H	3.552564	2.535288	4.589106
H	5.683341	-2.106665	1.211984
H	5.767022	-2.912344	-0.334537
H	4.988762	-1.324838	-0.193577
H	4.046398	-1.677725	2.380291
H	2.616622	-1.289461	1.441167
H	2.616178	-2.679715	2.508681
H	4.787083	-4.278983	2.066031
H	3.219577	-4.900249	1.531657
H	4.608983	-4.973367	0.444003
H	5.442057	1.714012	-3.463253
H	6.370533	1.094439	-2.099768
H	4.896408	0.265357	-2.615192
H	3.406487	3.152810	-3.110234
H	2.780851	1.545302	-2.799663
H	2.444233	2.850362	-1.671735
H	5.314057	3.985197	-2.379016
H	4.547292	4.178533	-0.793163
H	6.153999	3.460916	-0.917390
H	1.701478	5.079742	-2.648706
H	0.818468	3.571464	-2.867738
H	0.033063	5.104072	-3.247381
H	2.133426	4.674350	-0.148556
H	0.756068	4.425225	0.935022
H	1.293977	3.132893	-0.123424
H	1.148493	6.671864	-0.983611
H	-0.510683	6.794797	-1.564632
H	-0.179317	6.512138	0.159717
H	-3.434190	0.841462	-4.855215
H	-3.049311	2.524134	-4.464757
H	-1.813790	1.265291	-4.344006
H	-3.350571	-0.921348	-2.908523
H	-1.825055	-0.316065	-2.283573

H	-3.240551	-0.347426	-1.244381
H	-5.126555	0.292141	-3.429747
H	-5.337423	1.037564	-1.852235
H	-5.291281	2.037347	-3.324901

2BTMSP

E= -2515.439857

C	3.031071	0.186453	0.261808
C	1.716058	-0.126817	0.698037
C	1.459606	-1.270127	1.489960
C	2.512624	-2.171652	1.685687
C	3.763887	-1.967070	1.133771
C	4.020886	-0.780105	0.464968
C	0.609305	0.723927	0.258681
C	-0.609339	0.723988	-0.258674
C	-1.716051	-0.126827	-0.698004
C	-1.459534	-1.270200	-1.489809
C	-2.512519	-2.171756	-1.685553
C	-3.763794	-1.967195	-1.133650
C	-4.020848	-0.780206	-0.464927
C	-3.031084	0.186434	-0.261851
Si	0.098766	-1.675067	-2.508285
C	-0.522151	-2.396765	-4.137074
Si	-3.669652	1.887481	0.334675
C	-5.537110	1.759944	0.552530
Si	-0.098736	-1.674963	2.508428
C	0.521796	-2.396331	4.137415
Si	3.669552	1.887560	-0.334773
C	3.376107	3.125709	1.049817
P	0.000062	2.411059	0.000039
C	1.109472	-0.156495	-2.931733
C	1.158712	-2.976563	-1.672753
C	-3.019159	2.519056	1.979235
C	-3.376290	3.125741	-1.049830
C	-1.109560	-0.156307	2.931224
C	-1.158170	-2.976753	1.672846
C	5.537012	1.760116	-0.552618
C	3.019096	2.518998	-1.979449
H	-2.350042	-3.065380	-2.278706
H	-4.548901	-2.703622	-1.264067
H	-5.028558	-0.599697	-0.110582
H	2.350190	-3.065270	2.278852
H	4.549020	-2.703466	1.264201
H	5.028584	-0.599572	0.110600
H	5.913975	2.740845	-0.860025
H	6.052230	1.486717	0.372166
H	5.821977	1.045606	-1.330449
H	3.814671	4.093607	0.787313
H	2.319455	3.284727	1.272233
H	3.860264	2.776026	1.966594
H	3.462765	3.503756	-2.162159
H	3.321706	1.860743	-2.798242
H	1.934458	2.629863	-2.016380
H	-0.323801	-2.512393	4.822903
H	0.981992	-3.381303	4.022522
H	1.251599	-1.736777	4.615539
H	-1.679447	-0.346546	3.846114
H	-0.484480	0.723800	3.104670
H	-1.821725	0.083179	2.143168
H	-1.905810	-3.354038	2.377949
H	-1.683863	-2.580462	0.803739
H	-0.548983	-3.823848	1.343127

H	1.680750	-0.347829	-3.845527
H	0.484243	0.723097	-3.107269
H	1.820441	0.084409	-2.143057
H	0.322847	-2.510439	-4.823696
H	-0.980084	-3.382811	-4.022323
H	-1.253939	-1.738404	-4.613809
H	1.904187	-3.355855	-2.379095
H	1.687053	-2.579368	-0.805673
H	0.549474	-3.822482	-1.340154
H	-5.914088	2.740706	0.859820
H	-6.052323	1.486465	-0.372236
H	-5.822077	1.045515	1.330431
H	-3.462126	3.504222	2.161450
H	-3.322377	1.861351	2.798246
H	-1.934450	2.629158	2.016325
H	-3.816234	4.093154	-0.787854
H	-2.319586	3.285970	-1.271142
H	-3.859068	2.775415	-1.967085

{2BTMSP}$_2$

E= -5030.942340

C	-2.662654	3.086469	-0.977423
C	-3.219159	1.828109	-0.642207
C	-4.564124	1.739662	-0.192051
C	-5.170221	2.921723	0.240246
C	-4.525815	4.148173	0.170990
C	-3.328452	4.228465	-0.517752
C	-2.347685	0.653959	-0.639032
C	-2.166601	-0.581367	-0.247121
C	-2.669608	-1.809038	0.379867
C	-3.030098	-1.825356	1.745328
C	-3.509426	-3.035584	2.267272
C	-3.644699	-4.171773	1.494221
C	-3.247382	-4.137251	0.166979
C	-2.727966	-2.980440	-0.416940
Si	-2.798733	-0.527442	3.129076
C	-2.541808	1.256819	2.644780
Si	-1.978878	-3.236936	-2.155543
C	-2.726694	-4.806292	-2.885635
Si	-5.794383	0.311837	-0.483469
C	-5.219209	-0.820047	-1.858415
Si	-1.327692	3.414776	-2.306457
C	-1.883790	4.989878	-3.186023
P	-0.609505	0.093449	-0.969960
P	0.609608	0.093378	0.970259
C	2.166638	-0.581433	0.247235
C	2.669535	-1.808993	-0.380042
C	3.029945	-1.824997	-1.745530
C	3.509007	-3.035182	-2.267827
C	3.644263	-4.171563	-1.495063
C	3.247114	-4.137310	-0.167764
C	2.727811	-2.980604	0.416464
Si	2.798790	-0.526750	-3.128955
C	2.541626	1.257402	-2.644399
Si	1.978630	-3.237509	2.154940
C	2.315134	-1.899689	3.430809
C	2.347788	0.653856	0.639221
C	3.219357	1.827929	0.642429
C	4.564333	1.739378	0.192299
C	5.170564	2.921418	-0.239862
C	4.526281	4.147928	-0.170548
C	3.328870	4.228291	0.518102

C	2.662920	3.086327	0.977629
Si	5.794362	0.311390	0.483773
C	5.218932	-0.820336	1.858749
Si	1.327870	3.414607	2.306616
C	1.883723	4.989883	3.186052
C	4.317524	-0.568435	-4.244703
C	1.323027	-1.102895	-4.140002
C	0.144523	-3.536933	1.904517
C	2.726301	-4.807121	2.884658
C	6.282799	-0.641612	-1.053785
C	7.362412	1.148602	1.118969
C	-0.384138	3.769833	1.633394
C	1.321203	2.073331	3.629191
C	-4.317197	-0.569572	4.245169
C	-1.322803	-1.103712	4.139786
C	-2.315411	-1.898729	-3.431006
C	-0.144776	-3.536481	-1.905352
C	-6.282771	-0.640775	1.054324
C	-7.362296	1.149283	-1.118661
C	0.384253	3.770355	-1.633220
C	-1.320983	2.073451	-3.628964
H	3.786091	-3.092469	-3.315602
H	4.036747	-5.085390	-1.927161
H	3.331598	-5.045338	0.418054
H	6.184130	2.892182	-0.626873
H	4.998186	5.041400	-0.563703
H	2.908331	5.208625	0.712967
H	-3.786656	-3.093074	3.314994
H	-4.037336	-5.085660	1.926056
H	-3.331896	-5.045130	-0.419066
H	-6.183771	2.892557	0.627306

H	-4.997596	5.041666	0.564246
H	-2.907844	5.208776	-0.712586
H	-1.165580	-0.440111	4.996332
H	-1.472650	-2.118630	4.519983
H	-0.413717	-1.097480	3.535338
H	-4.192840	0.184618	5.029278
H	-5.231954	-0.332117	3.698060
H	-4.462699	-1.531778	4.742450
H	-2.343835	1.818897	3.564103
H	-1.683671	1.390107	1.990407
H	-3.414250	1.697609	2.160706
H	-6.083690	-1.355940	-2.262918
H	-4.769144	-0.245279	-2.672228
H	-4.496537	-1.560565	-1.522091
H	-7.130856	-1.290914	0.815697
H	-5.478963	-1.268392	1.438041
H	-6.603440	0.043112	1.845940
H	-8.060110	0.382193	-1.469969
H	-7.879516	1.729443	-0.349777
H	-7.147694	1.818434	-1.956552
H	1.103896	3.848849	-2.453176
H	0.384961	4.724294	-1.098862
H	0.754531	3.012572	-0.943684
H	-1.223167	5.164009	-4.041835
H	-2.905901	4.902077	-3.565079
H	-1.832078	5.879549	-2.552731
H	-1.116097	2.535101	-4.600099
H	-0.567475	1.303982	-3.456321
H	-2.294748	1.579503	-3.698557
H	1.223051	5.163942	4.041842
H	2.905834	4.902282	3.565152

H	1.831846	5.879494	2.552691
H	1.116822	2.535110	4.600375
H	0.567253	1.304218	3.456832
H	2.294742	1.578913	3.698498
H	-1.103847	3.847946	2.453326
H	-0.385143	4.723868	1.099205
H	-0.754146	3.012084	0.943675
H	8.060222	0.381391	1.470016
H	7.879563	1.728928	0.350165
H	7.147941	1.817564	1.957047
H	6.083367	-1.356113	2.263507
H	4.768619	-0.245543	2.672405
H	4.496417	-1.560962	1.522316
H	7.130076	-1.292579	-0.814531
H	5.478676	-1.268479	-1.438057
H	6.604713	0.041950	-1.845174
H	-1.702854	-2.110709	-4.313695
H	-3.363297	-1.923487	-3.739643
H	-2.079801	-0.886579	-3.102882
H	0.357777	-3.725738	-2.858053
H	0.352396	-2.688054	-1.433952
H	0.010672	-4.407091	-1.261534
H	-2.383482	-4.907354	-3.920254
H	-2.424084	-5.712958	-2.355026
H	-3.820005	-4.766773	-2.904298
H	2.344144	1.819663	-3.563716
H	1.683128	1.390563	-1.990480
H	3.413813	1.698106	-2.159801
H	1.165727	-0.438948	-4.996266
H	1.472992	-2.117644	-4.520601
H	0.413923	-1.096993	-3.535567
H	4.193045	0.185588	-5.028953
H	5.232010	-0.330459	-3.697361
H	4.463615	-1.530653	-4.741782
H	2.383066	-4.908394	3.919249
H	2.423595	-5.713627	2.353832
H	3.819616	-4.767727	2.903355
H	1.702724	-2.112082	4.313502
H	3.363063	-1.924355	3.739295
H	2.079277	-0.887472	3.103055
H	-0.358228	-3.725945	2.857157
H	-0.352489	-2.688564	1.432845
H	-0.010882	-4.407661	1.260847

8BTMSP

E= -2592.886045

C	3.582286	0.959732	0.103711
C	2.765441	-0.191780	0.016929
C	3.325886	-1.457335	-0.270712
C	4.709840	-1.536259	-0.468752
C	5.524605	-0.420533	-0.390784
C	4.958671	0.809574	-0.105143
C	1.295720	-0.075445	0.273073
P	-0.000010	0.000012	-0.986036
C	-1.295748	0.075449	0.273068
C	-0.728791	0.043065	1.504747
C	0.728758	-0.043071	1.504750
Si	2.385160	-3.101854	-0.429125
C	1.158539	-3.370250	0.966567
Si	2.983816	2.728958	0.454855
C	2.277253	2.922553	2.187650
C	-2.765469	0.191784	0.016924

C	-3.325908	1.457339	-0.270709
C	-4.709866	1.536273	-0.468716
C	-5.524633	0.420547	-0.390751
C	-4.958698	-0.809567	-0.105137
C	-3.582311	-0.959725	0.103708
Si	-2.385146	3.101835	-0.429138
C	-1.586046	3.215987	-2.127701
Si	-2.983808	-2.728948	0.454843
C	-1.788142	-3.290550	-0.878711
C	1.586057	-3.216039	-2.127684
C	3.626451	-4.515164	-0.315173
C	4.462676	3.893316	0.370504
C	1.788152	3.290582	-0.878693
C	-2.277229	-2.922531	2.187632
C	-4.462641	-3.893339	0.370508
C	-3.626402	4.515176	-0.315203
C	-1.158532	3.370218	0.966563
H	1.303688	-0.094410	2.423438
H	-1.303728	0.094381	2.423434
H	5.168899	-2.495432	-0.680770
H	6.593898	-0.510547	-0.547446
H	5.609620	1.674269	-0.043866
H	-5.609650	-1.674259	-0.043852
H	-6.593928	0.510566	-0.547396
H	-5.168923	2.495447	-0.680730
H	1.014890	-4.144282	-2.226345
H	0.913738	-2.384632	-2.349668
H	2.368422	-3.217704	-2.893007
H	0.745866	-4.382392	0.909993
H	1.659090	-3.263254	1.933853
H	0.326972	-2.669881	0.943203

H	3.081812	-5.464766	-0.318836
H	4.322978	-4.539090	-1.157725
H	4.209839	-4.472695	0.609237
H	1.436290	4.308017	-0.682760
H	2.289083	3.285676	-1.851318
H	0.911084	2.649038	-0.957853
H	2.086715	3.981722	2.388789
H	1.343254	2.383062	2.342304
H	3.002171	2.568038	2.926981
H	4.111930	4.916304	0.540400
H	5.212837	3.674515	1.135914
H	4.953496	3.873614	-0.606608
H	-1.436186	-4.307942	-0.682720
H	-2.289107	-3.285756	-1.851319
H	-0.911130	-2.648935	-0.957947
H	-2.086661	-3.981697	2.388760
H	-1.343242	-2.383018	2.342284
H	-3.002152	-2.568043	2.926971
H	-4.111863	-4.916317	0.540400
H	-5.212795	-3.674562	1.135931
H	-4.953476	-3.873654	-0.606596
H	-1.014873	4.144224	-2.226384
H	-0.913739	2.384570	-2.349683
H	-2.368422	3.217649	-2.893013
H	-3.081738	5.464764	-0.318863
H	-4.322921	4.539120	-1.157761
H	-4.209799	4.472726	0.609203
H	-0.745846	4.382355	0.909979
H	-1.659099	3.263250	1.933843
H	-0.326971	2.669839	0.943219

{8BTMSP}$_2$

E= -5185.817422

C	-3.579427	2.138396	-1.584545
C	-3.204051	0.778121	-1.451050
C	-4.147238	-0.188657	-1.015296
C	-5.367712	0.270981	-0.508744
C	-5.701337	1.613007	-0.503357
C	-4.834776	2.516918	-1.087801
C	-1.829238	0.360536	-1.852703
C	-1.460145	0.216596	-3.152348
C	-0.152070	-0.312166	-3.407830
C	0.553259	-0.682403	-2.310053
C	1.996614	-1.055371	-2.364022
C	2.430387	-2.394093	-2.391603
C	3.813728	-2.629879	-2.405360
C	4.734720	-1.600231	-2.418670
C	4.282188	-0.291597	-2.486942
C	2.920040	0.013471	-2.477741
Si	1.429424	-3.978672	-2.709183
C	1.853447	-4.477949	-4.474825
Si	2.466269	1.827646	-2.837319
C	4.022039	2.876024	-2.716523
Si	-4.157770	-2.044215	-1.470871
C	-3.689904	-2.218870	-3.284228
Si	-2.731933	3.604872	-2.482263
C	-4.122166	4.603041	-3.282493
P	-0.578949	-0.586923	-0.912414
P	0.555432	-0.401028	0.953816
C	-0.544601	-0.459126	2.367818
C	-1.932521	-0.979088	2.525911
C	-2.966579	-0.017807	2.597548
C	-4.277223	-0.477112	2.758804
C	-4.559116	-1.824788	2.911128
C	-3.518290	-2.737498	2.934245
C	-2.187812	-2.349434	2.733288
Si	-2.707814	1.863082	2.744185
C	-1.553087	2.588518	1.462192
Si	-0.876774	-3.719042	2.865564
C	-1.698776	-5.246663	3.601135
C	1.741967	0.713937	1.777713
C	3.089333	1.184949	1.345936
C	3.377795	2.573766	1.361246
C	4.632296	2.978392	0.883382
C	5.571712	2.076815	0.418371
C	5.311841	0.723424	0.529229
C	4.098607	0.240338	1.032206
Si	2.389515	4.052049	2.082127
C	1.296237	3.732339	3.575951
Si	4.205138	-1.556208	1.673714
C	5.887513	-2.253330	1.184379
C	1.394648	0.666982	3.093606
C	0.136437	0.073650	3.415971
C	-4.377185	2.714564	2.587708
C	-2.131294	2.293570	4.488934
C	-0.209413	-4.235993	1.188773
C	0.515790	-3.248605	4.039331
C	3.665151	5.308670	2.684189
C	1.345574	4.904744	0.781118
C	4.177597	-1.448660	3.554198
C	2.944380	-2.829225	1.136808
C	2.032402	-5.324687	-1.543122
C	-0.428523	-3.829331	-2.594576

C	1.920227	2.018338	-4.632903
C	1.200961	2.534041	-1.654786
C	-3.163938	-3.221384	-0.410261
C	-5.941761	-2.645580	-1.345321
C	-1.861646	4.723627	-1.256526
C	-1.559196	3.233117	-3.901510
H	-2.153495	0.435144	-3.955613
H	0.247154	-0.414054	-4.409387
H	-0.241771	0.023457	4.429126
H	2.077913	1.013693	3.859414
H	-5.095247	0.234590	2.784615
H	-5.582388	-2.159197	3.045413
H	-3.750834	-3.782020	3.110197
H	4.887430	4.031516	0.867339
H	6.516353	2.426940	0.016435
H	6.088303	0.025403	0.241312
H	4.182428	-3.651591	-2.416657
H	5.798417	-1.813518	-2.415867
H	5.011402	0.508121	-2.547890
H	-6.096407	-0.440868	-0.140196
H	-6.650164	1.944977	-0.096024
H	-5.150245	3.551860	-1.158383
H	-2.963143	-4.138157	-0.972818
H	-2.216015	-2.798452	-0.084910
H	-3.732601	-3.484438	0.482838
H	-3.914978	-3.237388	-3.616573
H	-4.269469	-1.525654	-3.901240
H	-2.632082	-2.035581	-3.466216
H	-5.980254	-3.673577	-1.720948
H	-6.304949	-2.666264	-0.313475
H	-6.633497	-2.046344	-1.943398
H	-1.245498	4.197303	-4.317057
H	-0.663647	2.692798	-3.606076
H	-2.052235	2.679430	-4.705091
H	-3.679574	5.329031	-3.972073
H	-4.796306	3.964179	-3.860305
H	-4.724817	5.166629	-2.565869
H	-1.545059	5.646694	-1.752501
H	-2.519039	4.990215	-0.424265
H	-0.975933	4.243120	-0.843775
H	-1.524284	3.675858	1.583300
H	-0.531048	2.215940	1.542716
H	-1.917825	2.376011	0.455650
H	-2.295911	3.362240	4.663073
H	-2.722656	1.742998	5.227163
H	-1.077542	2.089392	4.677102
H	-4.217976	3.797656	2.565731
H	-4.901474	2.436281	1.673742
H	-5.024230	2.497789	3.442989
H	0.625613	-4.928383	1.331521
H	-0.982692	-4.753320	0.615498
H	0.150510	-3.396317	0.591795
H	1.183559	-4.106025	4.172309
H	1.114061	-2.407903	3.689897
H	0.109871	-2.984216	5.020582
H	-0.937989	-6.019136	3.752356
H	-2.163256	-5.044378	4.570425
H	-2.459760	-5.667632	2.937531
H	1.351613	-5.412347	-4.745711
H	1.534375	-3.707200	-5.182800
H	2.929500	-4.622716	-4.605563
H	-0.874147	-4.819825	-2.734722

H	-0.763464	-3.441988	-1.634434
H	-0.814444	-3.173659	-3.376916
H	1.480369	-6.251256	-1.729544
H	3.095158	-5.542288	-1.680082
H	1.877825	-5.044397	-0.500145
H	1.926506	3.081760	-4.894442
H	2.628871	1.510314	-5.294413
H	0.922974	1.634576	-4.845437
H	1.058136	3.596529	-1.871950
H	0.226802	2.047738	-1.709770
H	1.565540	2.450039	-0.629778
H	3.746775	3.927180	-2.851843
H	4.513227	2.781793	-1.748031
H	4.743817	2.626519	-3.500058
H	0.957010	5.845156	1.185246
H	1.920607	5.130222	-0.120793
H	0.495235	4.287399	0.495751
H	0.910314	4.703707	3.904561
H	0.442736	3.094693	3.355576
H	1.848234	3.300190	4.414554
H	3.147283	6.067707	3.279193
H	4.427173	4.849616	3.320373
H	4.173389	5.831577	1.869734
H	2.038654	-2.777542	1.735685
H	2.664931	-2.719560	0.090008
H	3.383201	-3.824042	1.271434
H	5.965159	-3.265650	1.593744
H	5.999065	-2.330372	0.099105
H	6.726097	-1.675512	1.581256
H	4.423125	-2.423838	3.987024
H	4.908616	-0.721276	3.918820
H	3.193820	-1.160045	3.930624

12BTMSP

E= -2632.194213

C	-3.638393	0.723669	-0.066271
C	-2.769262	-0.391615	-0.093738
C	-3.190108	-1.627961	-0.636311
C	-4.475311	-1.699721	-1.188326
C	-5.330530	-0.612356	-1.196654
C	-4.914515	0.576680	-0.625400
C	-1.387332	-0.257703	0.494201
P	-0.023148	0.042281	-0.649713
C	1.380088	0.154599	0.481173
C	1.283395	0.040242	1.817623
C	0.041715	-0.237690	2.595893
C	-1.230456	-0.364949	1.827372
Si	-2.229668	-3.271814	-0.618336
C	-3.417242	-4.661115	-1.080919
Si	-3.251029	2.465298	0.597731
C	-2.528310	2.509607	2.336073
C	2.720701	0.380123	-0.159179
C	3.047345	1.651802	-0.678051
C	4.281139	1.802078	-1.322665
C	5.172868	0.751767	-1.449699
C	4.851889	-0.474644	-0.894501
C	3.634304	-0.694588	-0.237704
Si	2.025582	3.251593	-0.560038
C	1.059683	3.475688	-2.155968
Si	3.496110	-2.404796	0.582953
C	4.509548	-3.627954	-0.431307
C	-0.868145	-3.305849	-1.913564

C	-1.622362	-3.680462	1.111905
C	-2.140303	3.345823	-0.629728
C	-4.864654	3.434143	0.703726
C	4.265222	-2.283500	2.297593
C	1.770294	-3.115686	0.746545
C	0.947388	3.336965	0.977020
C	3.235772	4.691278	-0.420623
H	-0.086202	0.541967	3.363140
H	-2.115483	-0.561961	2.429090
H	2.191372	0.148676	2.407985
H	0.190529	-1.157074	3.186011
H	-4.826977	-2.632426	-1.614042
H	-6.319846	-0.696113	-1.632873
H	-5.606884	1.410566	-0.615968
H	5.572944	-1.280881	-0.977350
H	6.118417	0.892066	-1.961752
H	4.560034	2.767723	-1.731724
H	2.682776	5.609014	-0.196295
H	3.960630	4.532892	0.382993
H	3.790316	4.867821	-1.346152
H	0.427531	4.367924	-2.121760
H	1.764618	3.590621	-2.985671
H	0.425582	2.615166	-2.379788
H	0.502832	4.334024	1.056174
H	0.139189	2.608575	0.975137
H	1.553110	3.163720	1.871764
H	4.349563	-3.273321	2.757349
H	5.267251	-1.848125	2.245513
H	3.664365	-1.656603	2.962433
H	4.347576	-4.637680	-0.041315
H	4.209883	-3.624271	-1.483261
H	5.584303	-3.433642	-0.385072
H	1.839776	-4.190436	0.942397
H	1.228106	-2.661467	1.575717
H	1.175222	-2.979171	-0.157214
H	-1.823940	4.324132	-0.255594
H	-1.245373	2.764084	-0.851405
H	-2.673616	3.495843	-1.573159
H	-2.612185	3.529948	2.724572
H	-3.086228	1.852189	3.009376
H	-1.476467	2.228361	2.375122
H	-4.655785	4.414193	1.144227
H	-5.319218	3.609220	-0.275052
H	-5.603717	2.938399	1.340137
H	-1.127797	-4.656637	1.117101
H	-0.919658	-2.944858	1.497391
H	-2.475195	-3.729436	1.796318
H	-2.889453	-5.615952	-0.989497
H	-4.285977	-4.700447	-0.417449
H	-3.775883	-4.586913	-2.111407
H	-0.309947	-4.245748	-1.854034
H	-1.320716	-3.246881	-2.908367
H	-0.162651	-2.477252	-1.831472

{12BTMSP}$_2$

E= -5264.387167

C	2.200059	-3.258103	1.308074
C	2.507094	-1.888864	1.115736
C	3.470061	-1.510658	0.154810
C	3.857975	-2.452915	-0.802920
C	3.380993	-3.750214	-0.770559
C	2.637840	-4.157704	0.327120

C	1.808912	-0.819613	1.901389
C	1.885084	-0.796973	3.231844
C	1.273329	0.173280	4.181772
C	0.549697	1.322978	3.573306
C	0.343351	1.588477	2.282642
C	-0.369541	2.859322	1.926913
C	0.359259	4.013078	1.545360
C	-0.367954	5.091768	1.025290
C	-1.751773	5.071841	0.938817
C	-2.442560	4.007366	1.491636
C	-1.775386	2.889091	2.007403
Si	2.169426	4.410826	2.010629
C	2.594090	3.786609	3.735444
Si	-2.857257	1.696106	3.017989
C	-2.930960	2.433462	4.751931
Si	4.674442	-0.057368	0.357393
C	4.624027	1.125913	-1.088019
Si	1.679336	-4.113035	2.932214
C	0.324731	-3.356411	4.015988
P	1.070343	0.570987	0.913222
P	-0.420703	-0.542222	-0.540973
C	-2.151473	0.193876	-0.465032
C	-3.486830	-0.487422	-0.269208
C	-4.581361	0.094622	-0.993003
C	-5.889812	-0.174190	-0.575955
C	-6.166120	-1.037331	0.464100
C	-5.115787	-1.761046	0.988540
C	-3.775681	-1.556624	0.623104
Si	-4.624544	1.157615	-2.587125
C	-4.791495	3.001899	-2.207387
Si	-2.856072	-3.119983	1.219294
C	-3.203577	-3.465497	3.041876
C	-2.224227	1.513777	-0.696390
C	-1.153557	2.337907	-1.326326
C	-0.332643	1.563544	-2.308735
C	0.135798	0.337490	-2.085449
C	1.146163	-0.319201	-2.975098
C	1.080596	-1.705250	-3.254259
C	2.182939	-2.316240	-3.864680
C	3.299457	-1.602765	-4.250065
C	3.282962	-0.225966	-4.110652
C	2.226985	0.453609	-3.491742
Si	-0.386313	-2.920225	-3.279051
C	-2.063371	-2.351243	-2.671009
Si	2.376548	2.348536	-3.729079
C	4.145303	2.791941	-4.212850
C	-3.269091	0.865979	-3.852213
C	-6.207601	0.701475	-3.514085
C	-3.644075	-4.506165	0.210617
C	-1.023882	-3.316167	1.000041
C	1.351954	2.807103	-5.249756
C	1.936157	3.474594	-2.277542
C	-0.634037	-3.293823	-5.112791
C	0.112370	-4.509778	-2.403084
C	2.306841	6.289713	2.099792
C	3.477795	3.830068	0.805943
C	-4.604994	1.723979	2.328969
C	-2.270792	-0.079452	3.181436
C	4.549054	0.808898	2.022238
C	6.382712	-0.859942	0.361320
C	1.202038	-5.902723	2.564894
C	3.264671	-4.226959	3.951816

H	-0.480327	2.757519	-0.566014
H	-0.114984	2.065037	-3.241503
H	-3.157860	2.026818	-0.506151
H	-1.611370	3.204673	-1.808807
H	2.062088	0.569166	4.837475
H	0.181838	2.054463	4.290283
H	2.494703	-1.557409	3.711104
H	0.599034	-0.367696	4.863850
H	0.154205	5.984897	0.701357
H	-2.285879	5.910974	0.506557
H	-3.527036	4.046823	1.517577
H	4.561696	-2.169918	-1.579789
H	3.646218	-4.458488	-1.547644
H	2.391185	-5.210086	0.415644
H	-6.723623	0.295940	-1.084280
H	-7.182479	-1.202218	0.802883
H	-5.356293	-2.540116	1.704475
H	4.132368	0.331255	-4.487314
H	4.153315	-2.102242	-4.694906
H	2.162382	-3.385493	-4.056364
H	-2.958888	-4.515590	3.235734
H	-2.572622	-2.856689	3.691384
H	-4.242753	-3.312968	3.340779
H	-3.165080	-5.461567	0.449067
H	-4.714914	-4.605613	0.405776
H	-3.518730	-4.327272	-0.861005
H	-0.785244	-4.264280	1.489615
H	-0.716556	-3.391320	-0.038823
H	-0.430320	-2.532143	1.463293
H	1.003795	4.017832	-2.452547
H	1.832954	2.939511	-1.332227
H	2.730047	4.216397	-2.158163
H	4.195202	3.881108	-4.316204
H	4.876801	2.499094	-3.456738
H	4.446554	2.366150	-5.173406
H	1.473605	3.872938	-5.469712
H	1.717194	2.245171	-6.114667
H	0.282010	2.603718	-5.161584
H	-1.419354	-4.045610	-5.243781
H	-0.942700	-2.389776	-5.647125
H	0.274279	-3.666897	-5.592521
H	-0.765340	-5.141254	-2.231852
H	0.823158	-5.087461	-3.001349
H	0.583332	-4.305206	-1.439601
H	-2.830149	-2.933251	-3.193611
H	-2.201468	-2.510253	-1.604532
H	-2.251655	-1.299282	-2.880778
H	-3.342006	1.652841	-4.611036
H	-2.255096	0.871066	-3.457447
H	-3.430800	-0.089218	-4.359327
H	-6.140081	1.113960	-4.526325
H	-6.329696	-0.381587	-3.604115
H	-7.115322	1.109562	-3.061779
H	-5.247091	3.504733	-3.066470
H	-5.450017	3.157864	-1.346706
H	-3.844080	3.503686	-1.998482
H	4.461341	3.882516	1.283032
H	3.312024	2.803616	0.486635
H	3.498729	4.461606	-0.084061
H	3.295915	6.547862	2.491969
H	2.213762	6.768614	1.121019
H	1.558778	6.727797	2.766426

H	3.500138	4.301310	4.072099
H	1.797927	4.008923	4.451461
H	2.792146	2.714721	3.763907
H	-3.589923	1.838881	5.393181
H	-1.944624	2.462938	5.223386
H	-3.318144	3.456232	4.726346
H	-5.206571	0.966896	2.840900
H	-5.091413	2.690598	2.488049
H	-4.640215	1.490604	1.263584
H	-3.148817	-0.713147	3.321597
H	-1.728487	-0.440388	2.306802
H	-1.621728	-0.197323	4.051906
H	0.797478	-6.351457	3.477732
H	0.445754	-6.002114	1.782437
H	2.069805	-6.498780	2.269514
H	3.087484	-4.791924	4.872986
H	4.039582	-4.746813	3.380804
H	3.669720	-3.251024	4.232727
H	-0.469653	-4.088727	4.183333
H	0.738971	-3.090019	4.993310
H	-0.133163	-2.462500	3.594136
H	5.305221	1.968496	-0.936911
H	4.919496	0.597731	-1.997968
H	3.617666	1.511868	-1.244716
H	5.357583	1.543716	2.092422
H	3.607228	1.328700	2.186926
H	4.680427	0.082293	2.829087
H	7.153660	-0.103587	0.541890
H	6.459103	-1.615683	1.148445
H	6.611803	-1.347323	-0.590091

21BTMSP

E= -2670.269297

C	2.939928	-1.621587	0.907784
C	2.674182	-0.383484	0.284168
C	3.520385	0.732440	0.465806
C	4.597897	0.597168	1.349841
C	4.850175	-0.591122	2.013083
C	4.041996	-1.688557	1.769776
C	1.454450	-0.195907	-0.583831
P	0.013140	0.166558	0.432228
C	-1.420057	0.347599	-0.644001
C	-1.549589	0.427023	-1.993226
C	-0.570204	0.328350	-3.048308
C	0.741513	0.004784	-3.023440
C	1.635923	-0.279484	-1.927872
Si	3.467674	2.368571	-0.509221
C	4.335511	2.102192	-2.159291
Si	2.026315	-3.263641	0.597935
C	0.365304	-3.345419	1.471609
C	-2.696159	0.401083	0.158639
C	-3.460502	-0.778537	0.264296
C	-4.633203	-0.731198	1.030853
C	-5.043830	0.424120	1.668439
C	-4.289018	1.577405	1.527802
C	-3.108225	1.603903	0.776849
Si	-3.201724	-2.485836	-0.540883
C	-4.647202	-2.740439	-1.719780
Si	-2.263819	3.307763	0.653854
C	-0.875171	3.462296	1.911248
C	1.767788	3.086008	-0.838387
C	4.437512	3.659782	0.460646

C	1.885005	-3.622949	-1.243197
C	3.069233	-4.660086	1.314188
C	-1.708933	3.706381	-1.095437
C	-3.538502	4.622948	1.101810
C	-1.621495	-2.776472	-1.508371
C	-3.297488	-3.765574	0.836576
H	1.224169	-0.040546	-3.996017
H	2.639324	-0.557719	-2.242970
H	-2.564877	0.581793	-2.353973
H	-0.982568	0.506783	-4.037691
H	5.259851	1.438062	1.525378
H	5.683433	-0.667372	2.702971
H	4.280626	-2.623117	2.264559
H	-5.246280	-1.622620	1.129935
H	-5.953016	0.432400	2.259482
H	-4.639896	2.483311	2.009178
H	-0.375842	4.431933	1.815045
H	-1.293842	3.399003	2.920526
H	-0.126850	2.673168	1.820137
H	-1.347064	4.739018	-1.133948
H	-0.913055	3.059511	-1.458196
H	-2.555272	3.622324	-1.784498
H	-3.100571	5.610269	0.923298
H	-4.443566	4.545939	0.492489
H	-3.831394	4.586665	2.154808
H	-4.597213	-3.729829	-2.185126
H	-5.610030	-2.656283	-1.208358
H	-4.630502	-1.992700	-2.518455
H	-1.504794	-3.853701	-1.666900
H	-1.643760	-2.293227	-2.487173
H	-0.735462	-2.421558	-0.984741
H	-3.142778	-4.769874	0.429468
H	-2.529713	-3.586775	1.593471
H	-4.268738	-3.758331	1.338408
H	1.469423	-4.625891	-1.385890
H	1.252546	-2.920701	-1.784996
H	2.879344	-3.605714	-1.700376
H	-0.160368	-4.263829	1.191654
H	0.521055	-3.360790	2.554663
H	-0.282241	-2.496011	1.249893
H	4.073633	-4.689638	0.882196
H	3.165432	-4.599560	2.401879
H	4.496215	3.061696	-2.661104
H	5.309974	1.625183	-2.021250
H	3.742859	1.472523	-2.827815
H	1.885492	4.079510	-1.283879
H	1.194004	2.475470	-1.537140
H	1.183794	3.199160	0.076575
H	4.334878	4.629437	-0.036410
H	4.056096	3.768542	1.480166
H	5.506322	3.437245	0.519010
H	2.582547	-5.613548	1.085070

$\{21BTMSP\}_2$

E= -5340.536092

C	-4.635802	0.786002	0.053120
C	-3.480633	0.333507	0.771877
C	-3.624355	-0.009263	2.147768
C	-4.808165	0.379686	2.791738
C	-5.843937	1.027936	2.146768
C	-5.770182	1.165838	0.776929
C	-2.190154	0.317356	-0.007428

C	-2.119340	1.223914	-1.013778
C	-1.279493	1.283517	-2.177061
C	-0.723986	0.245365	-2.833763
C	-0.671871	-1.138436	-2.448924
C	-0.588991	-1.695059	-1.222441
C	-0.317198	-3.186791	-1.156536
C	0.913456	-3.671736	-1.680360
C	1.225901	-5.025650	-1.488937
C	0.378593	-5.895574	-0.832780
C	-0.871026	-5.440666	-0.460905
C	-1.270105	-4.109859	-0.650766
Si	2.071846	-2.878903	-2.983268
C	2.659631	-1.132136	-2.722505
Si	-3.160115	-4.026434	-0.863219
C	-3.367113	-4.087313	-2.734097
Si	-2.531351	-1.013790	3.362148
C	-1.071762	0.000201	3.945312
Si	-5.029178	0.809312	-1.830324
C	-4.190986	-0.507477	-2.878248
P	-0.670469	-0.693013	0.361842
P	1.152754	0.804413	0.060783
C	2.368090	0.247908	1.386174
C	3.431027	-0.693507	0.864091
C	3.257128	-2.094894	0.938210
C	4.138406	-2.901941	0.206605
C	5.202161	-2.372906	-0.503985
C	5.483509	-1.025884	-0.360972
C	4.633630	-0.160717	0.340479
Si	2.501732	-3.056357	2.399143
C	1.192747	-2.229875	3.434297
Si	5.560402	1.406681	0.912031
C	4.593117	2.748677	1.788181
C	2.483991	0.610827	2.671122
C	1.788639	1.604305	3.455628
C	1.193356	2.723071	3.022140
C	0.937758	3.201330	1.679980
C	0.739569	2.578814	0.498678
C	0.193060	3.465068	-0.607192
C	0.911734	3.751279	-1.798383
C	0.243357	4.419775	-2.831400
C	-1.063818	4.856670	-2.706333
C	-1.690654	4.712723	-1.484198
C	-1.091915	4.036725	-0.410584
Si	2.799427	3.732242	-2.044051
C	3.571646	2.047484	-2.253457
Si	-2.132517	4.250283	1.183501
C	-3.948707	4.400613	0.703367
C	1.799910	-4.710287	1.885838
C	4.008857	-3.333343	3.499823
C	6.801565	0.716087	2.151886
C	6.534924	2.197578	-0.492879
C	-2.041360	2.925701	2.504749
C	-1.626706	5.911683	1.919027
C	3.211763	4.628717	-3.653351
C	3.497550	4.820721	-0.676180
C	3.593285	-3.981859	-3.182435
C	1.210163	-2.982022	-4.669027
C	-3.955030	-5.558258	-0.105513
C	-4.115644	-2.574353	-0.211129
C	-3.508296	-1.318699	4.954657
C	-2.186106	-2.774938	2.789148
C	-6.871731	0.429908	-2.008096

C	-4.796419	2.512504	-2.612608
H	-0.303753	0.456740	-3.813993
H	-0.611361	-1.827413	-3.287067
H	-2.854732	2.018577	-0.994628
H	-1.255091	2.243865	-2.677307
H	0.854150	3.413189	3.790354
H	0.783021	4.278737	1.649788
H	3.289031	0.121244	3.221280
H	1.869969	1.477633	4.531362
H	-2.682930	5.132326	-1.367294
H	-1.566168	5.347208	-3.532758
H	0.761305	4.624011	-3.760285
H	4.009593	-3.980475	0.226579
H	5.848839	-3.012436	-1.094054
H	6.406250	-0.643515	-0.786830
H	2.161852	-5.417368	-1.866954
H	0.669460	-6.924619	-0.653499
H	-1.570853	-6.153001	-0.041857
H	-6.634167	1.562075	0.255593
H	-6.722511	1.350004	2.694383
H	-4.932308	0.179773	3.846905
H	4.581454	4.723699	-0.572549
H	3.277893	5.862681	-0.930120
H	3.045657	4.619368	0.294825
H	4.589933	2.143453	-2.639858
H	3.592851	1.424916	-1.361629
H	2.987306	1.507111	-3.001065
H	4.302443	4.701317	-3.724136
H	2.867647	4.088436	-4.540479
H	2.813173	5.645907	-3.691054
H	5.160138	3.683745	1.742600
H	3.614455	2.924367	1.345966
H	4.438770	2.501283	2.840166
H	7.377782	2.749132	-0.063748
H	6.940861	1.475778	-1.205559
H	5.924151	2.908023	-1.051223
H	7.399670	1.520707	2.591756
H	6.286539	0.195094	2.965058
H	7.483236	0.000725	1.683390
H	0.772434	-2.979975	4.112784
H	1.581121	-1.409957	4.038240
H	0.387528	-1.845873	2.811693
H	3.729045	-3.859693	4.418092
H	4.776075	-3.921679	2.988661
H	4.464230	-2.378956	3.783270
H	1.630469	-5.307276	2.788299
H	0.836105	-4.571369	1.392941
H	2.439125	-5.290041	1.217918
H	-1.578847	-3.276014	3.548959
H	-3.136483	-3.315455	2.729609
H	-1.673581	-2.870694	1.833603
H	-0.527676	-0.533704	4.728011
H	-0.365075	0.262022	3.160811
H	-1.457154	0.930391	4.372152
H	-2.887458	-1.963105	5.586872
H	-3.708571	-0.407585	5.525526
H	-4.454947	-1.840986	4.790061
H	-4.537319	4.438708	1.625681
H	-4.185055	5.299240	0.128329
H	-4.291480	3.526093	0.146724
H	-3.049529	2.695783	2.858070
H	-1.604645	2.001029	2.137076

H	-1.449706	3.255361	3.360961
H	-2.313726	6.186473	2.726127
H	-0.615636	5.899185	2.335440
H	-1.664517	6.700448	1.162235
H	-5.324785	2.527467	-3.571701
H	-5.231423	3.298217	-1.987446
H	-3.754094	2.774980	-2.807544
H	-4.757156	-1.440196	-2.824093
H	-4.198228	-0.174599	-3.921543
H	-3.160601	-0.723377	-2.607406
H	-7.074683	0.212296	-3.062107
H	-7.158177	-0.450390	-1.425230
H	-7.525590	1.256590	-1.718286
H	-4.423687	-4.116136	-3.018991
H	-2.917289	-3.206009	-3.199963
H	-2.876418	-4.973379	-3.146999
H	-5.149814	-2.648700	-0.563971
H	-4.135469	-2.513993	0.876012
H	-3.706883	-1.646914	-0.593171
H	-5.041236	-5.442702	-0.177529
H	-3.690866	-6.488116	-0.614761
H	-3.708024	-5.662891	0.955583
H	1.963410	-3.152737	-5.444984
H	0.498863	-3.812078	-4.710143
H	0.678548	-2.065152	-4.936685
H	3.358085	-0.881069	-3.528174
H	1.849805	-0.404844	-2.731885
H	3.188794	-1.009604	-1.779337
H	4.323460	-3.436110	-3.788985
H	4.076945	-4.237737	-2.239036
H	3.358294	-4.909233	-3.712724
